# Multi-Component Synthesis of New Fluorinated-Pyrrolo[3,4-*b*]pyridin-5-ones Containing the 4-Amino-7-chloroquinoline Moiety and In Vitro–In Silico Studies Against Human SARS-CoV-2

**DOI:** 10.3390/ijms26157651

**Published:** 2025-08-07

**Authors:** Roberto E. Blanco-Carapia, Ricardo Hernández-López, Sofía L. Alcaraz-Estrada, Rosa Elena Sarmiento-Silva, Montserrat Elemi García-Hernández, Nancy Viridiana Estrada-Toledo, Gerardo Padilla-Bernal, Leonardo D. Herrera-Zúñiga, Jorge Garza, Rubicelia Vargas, Eduardo González-Zamora, Alejandro Islas-Jácome

**Affiliations:** 1 Departamento de Química, Universidad Autónoma Metropolitana–Iztapalapa, Av. Ferrocarril San Rafael Atlixco 186, Col. Leyes de Reforma 1A Sección, Iztapalapa, Ciudad de México C.P. 09310, Mexico; edreyblanco@gmail.com (R.E.B.-C.); richi.quimera72@gmail.com (R.H.-L.); requiem900909@gmail.com (G.P.-B.); lherrera@cua.uam.mx (L.D.H.-Z.); jgarza@izt.uam.mx (J.G.); egz@xanum.uam.mx (E.G.-Z.); 2 División de Medicina Genómica, Centro Médico Nacional 20 de Noviembre, Instituto de Seguridad y Servicios Sociales de los Trabajadores del Estado (ISSSTE), Félix Cuevas 540, Col. Del Valle Sur, Benito Juárez, Ciudad de México C.P. 03100, Mexico; sofializeth@gmail.com; 3 Departamento de Microbiología e Inmunología, Facultad de Medicina, Veterinaria y Zootecnia, Universidad Nacional Autónoma de México, Av. Universidad 3000, Ciudad Universitaria, Coyoacán, Ciudad de México C.P. 04510, Mexico; rosass@unam.mx (R.E.S.-S.); elemi.gh@gmail.com (M.E.G.-H.); 4 Health Pharma Professional Research S.A de C.V., Av. Insurgentes Sur 662-Piso 3, Col. Del Valle, Benito Juárez, Ciudad de México C.P. 03100, Mexico; nefrtty-n_ancy@hotmail.com; 5 Departamento de Ciencias Naturales, Universidad Autónoma Metropolitana–Cuajimalpa, Vasco de Quiroga 4871, Col. Contadero, Cuajimalpa, Ciudad de México C.P. 05348, Mexico

**Keywords:** multi-component reactions, polyheterocycles, fluorinated-compounds, pyrrolo[3,4-*b*]pyridin-5-ones, quinoline, SARS-CoV-2, antiviral activity, drug design

## Abstract

A one-pot synthetic methodology that combines an Ugi-Zhu three-component reaction (UZ-3CR) with a cascade sequence (intermolecular *aza* Diels–Alder cycloaddition/intramolecular *N*-acylation/decarboxylation/dehydration) using microwave-heating conditions, ytterbium (III) triflate (Yb(OTf)_3_) as the catalyst, and chlorobenzene (for the first time in a multi-component reaction (MCR)) as the solvent, was developed to synthesize twelve new fluorinated-pyrrolo[3,4-*b*]pyridin-5-ones containing a 4-amino-7-chloroquinoline moiety, yielding 50–77% in 95 min per product, with associated atom economies around 88%, also per product. Additionally, by in vitro tests, compounds **19d** and **19i** were found to effectively stop early SARS-CoV-2 replication, IC_50_ = 6.74 µM and 5.29 µM, at 0 h and 1 h respectively, while cell viability remained above 90% relative to the control vehicle at 10 µM. Additional computer-based studies revealed that the most active compounds formed strong favorable interactions with important viral proteins (M_pro_, NTDα and NTDo) of coronavirus, supporting a two-pronged approach that affects both how the virus infects the cells and how it replicates its genetic material. Finally, quantum chemistry analyses of non-covalent interactions were performed from Density-Functional Theory (DFT) to better understand how the active compounds hit the virus.

## 1. Introduction

In medicinal chemistry, the concept of “privileged structure”, first introduced by Evans, refers to molecular fragments that enhance ligand binding across a wide range of biological receptors [1,2,3,4]. In the more recent literature, such a term describes distinct chemical entities found in natural, semi-synthetic, and synthetic bioactive compounds and current drugs that are pivotal for their pharmacological activity. These fragments act as ligands, mediating interactions with their respective receptors. Various heterocycles and polyheterocycles are privileged structures in medicinal chemistry due to their special stereoelectronic characteristics [5]. The isoindolin-2-one is a heterocycle with a remarkable broad-spectrum of biological activity, which exemplifies the potential of these privileged structures [6]. Similarly, quinoline is also a highly significant pharmacophoric heterocycle for developing pharmaceuticals, owing to its well-established antibacterial [7], antioxidant [8], anticancer [9], antiviral [10], anti-inflammatory [11], and other notable properties [12]. An innovative strategy for designing molecules with pharmacological potential involves the integration of one or more privileged structures into their main structural framework. In this context, multi-component reactions (MCRs) have emerged as very valuable synthetic tools enabling the rapid assembly of complex molecules, including bioactive polyheterocycles, from three or more reactants that undergo sequential transformations under uniform reaction conditions [13]. The one-pot approach of MCRs streamlines the synthetic process and offers considerable advantages over stepwise methodologies, including high atom economy, operational simplicity, and minimal purification requirements.

Moreover, as a part of our ongoing efforts to design and synthesize novel biologically active molecules, various methodologies for preparing discrete chemical libraries based on the Ugi-Zhu-three component reaction (UZ-3CR) have been reported [14]. This variant of the Ugi three-component reaction (U-3CR) consists of sequential combinations of aldehydes, amines, and α-isocyanoacetamides, enabling the efficient and rapid assembly of trisubstituted 5-aminooxazoles [15]. These heterocycles are highly versatile synthetic platforms for subsequent transformations such as *N*-acylations and cycloadditions [16]. This approach allows the synthesis of polyheterocycles containing the pyrrolo[3,4-*b*]pyridin-5-one core, a nitrogen-containing analog of the isoindolin-2-one core, demonstrating its remarkable pharmacological properties [17,18]. Building upon these observations, we describe the synthesis of twelve new fluorinated-pyrrolo[3,4-*b*]pyridin-5-ones containing a chloroquine fragment (4-amino-7-chloroquinoline) using an UZ-3CR coupled to a cascade-type post-Ugi sequence (intermolecular *aza*-Diels-Alder cycloaddition/intramolecular *N*-acylation/decarboxylation/dehydration). This method incorporates that specific chloroquine fragment, in addition to fluorinated aldehydes. Although chloroquine and its analogues demonstrate activity against SARS-CoV-2, their use is limited due to their high toxicity. Therefore, it would be of interest to synthesize derivatives incorporating a chloroquine fragment into the pyrrolo[3,4-*b*]pyridine-5-one nucleus behind trying to overcome this limitation [19]. It is worth highlighting that smart incorporations of fluorine atoms into the structure of bioactive molecules are well-documented to significantly enhance their biological activity by improving parameters like metabolic stability and lipophilicity, while strengthening non-covalent interactions to biological targets [20]. This study aims to determine whether polyheterocyclic compounds exhibit antiviral effects by inhibiting in vitro human coronavirus replication.

Because of the COVID-19 pandemic by human SARS-CoV-2, new ways of treating people are still needed. This one has led medicinal chemistry to focus on molecules that have been shown to be effective against other viruses or that could be used in other ways, like the antimalarial drug chloroquine and/or its derivatives [21,22]. Introducing additional functional groups into bioactive molecules can enhance their pharmacokinetic and pharmacodynamic properties, leading to more potent and selective drug candidates [23,24].

Computer-aided drug design (CADD) has emerged as an efficient strategy that supports the rational design and discovery of new drugs. CADD encompasses two main approaches: ligand-based drug design (LBDD), which builds predictive models from known ligands when solved protein structures are unavailable; and structure-based drug design (SBDD), which leverages the resulting 3D structures of targets to model their recognition and quantify ligand-protein interactions [25]. The latter is particularly valuable in the context of SARS-CoV-2, due to the availability of numerous solved structures of viral proteins. It enables the detailed study of interactions at specific binding sites and the assessment of safety profiles, helping to identify promising candidates with higher potency and lower toxicity in a shorter timeframe [26,27].

In this context, in silico studies are very important in drug discovery because they make it easier to quickly and cheaply check how potential compounds interact with important molecular targets [28]. Thus, molecular docking, molecular dynamics, and quantum chemistry simulations help figure out how the bioactive compounds bind to important viral proteins and give information about how stable but flexible complexes are formed [29].

This article aims to describe how to synthesize twelve new fluorinated-pyrrolo[3,4-*b*]pyridin-5-ones containing 4-amino-7-chloroquinoline moiety and how effective they are to hit human SARS-CoV-2. Consequently, both in vitro and in silico tests were utilized to thoroughly examine their interactions with essential viral proteins [30]. The main objective is then to enhance the efficacy and safety of compounds by combining the benefits of selective fluorination and the adaptability of the pyrrolo[3,4-*b*]pyridin-5-one framework with the established potential of an antimalarial chloroquine fragment for combating new viral infections. This article is especially relevant today as we seek alternatives to address the effects of SARS-CoV-2.

## 2. Results and Discussion

### 2.1. Synthesis

#### 2.1.1. Synthesis of Precursors

**Scheme 1:**
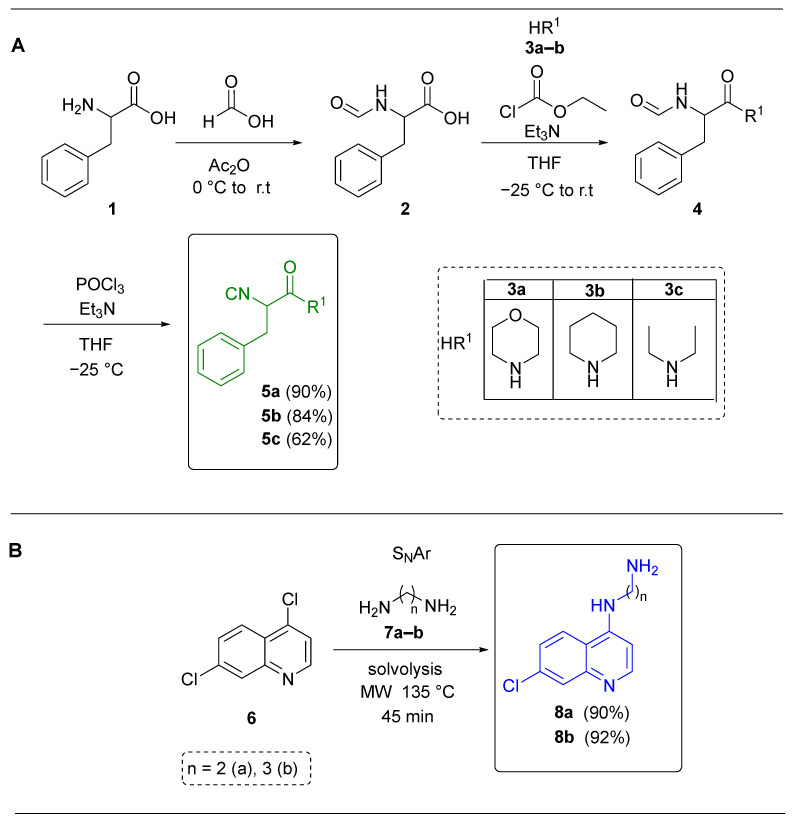
(**A**) Synthesis of α-isocyanoacetamides. (**B**) Synthesis of 4-amino-7-chloroquinolines. Color key: green (isocyanide moiety) and blue (amine moiety).

The synthesis of α-isocyanoacetamides **5a–c** and amines **8a–b** was mandatory for the present study. The α-isocyanoacetamides **5a–c** were prepared from racemic phenylalanine **1** (Scheme 1A) following the three-step protocol previously reported by Bienaymé and Zhu with a slight variation [31]. In contrast, the 4-amino-7-quinolines **8a–b** were obtained through a S_N_Ar from diamines **7a–b** and 4,7-dichloroquinoline **6**, enhanced by microwave (μw) irradiation (Scheme 1B).

#### 2.1.2. Optimization of Reaction Conditions

Once the components **5a–c** and **8a–b** were prepared, the reaction conditions for the UZ-3CR were optimized. This stage started determining the appropriate conditions for the formation of imine **10a** (Table 1, Conditions 1) and the subsequent nucleophilic addition of the α-isocyanoacetamide **5a** to imine **10a** (Table 1, Conditions 2), leading to the formation of the trisubstituted 5-aminooxazole **14a**. Accordingly, aldehyde **9a** was reacted with aminoquinoline **8a** in the presence of anhydrous Na_2_SO_4_, employed as a dehydrating agent, under constant stirring in dry toluene (PhMe) as the solvent (Table 1, Entry 1, Conditions 1). Next, InCl_3_ was added as an imine activator, followed by α-isocyanoacetamide **5a**, with the entire process assisted by μw irradiation (Table 1, Entry 1, Conditions 2).

However, the reaction did not proceed, likely due to the poor solubility of aminoquinoline **8a** in PhMe, even at 80 °C (Table 1, Entry 2, Conditions 1). To address this issue, the solvent was replaced by MeOH (Table 1, Entry 3), yielding the product with 8% yield. However, MeOH was not enough for synthesizing the target product in a good way, as it may affect the reactivity of α-isocyanoacetamide by stabilizing it through hydrogen bonding or promoting its self-tautomerization to 2-hydroxyoxazole. Consequently, a mixture of PhMe and MeOH (9:2 *v*/*v*) was employed, resulting in the formation of the expected product with a yield of 14% (Table 1, Entry 4) and 19% (Table 1, Entry 5). Subsequently, the solvent was replaced with an aprotic polar medium, specifically chlorobenzene (PhCl), which improved the yield to 41% (Table 1, Entry 6). Others Lewis acids were also tested as activators of the imine, particularly Sc(OTf)_3_ and Yb(OTf)_3_, resulting in 52% and 75% yields, respectively (Table 1, Entry 7 and 8). It is noteworthy that to the best of our knowledge, this is the first time in which PhCl is used successfully in an UZ-3CR, even in any MCR.

The observed differences in the yields using Lewis acids can be explained through Pearson’s hard and soft acids and bases (HSAB) theory [32]. Imines, like **10a**, possess two basic sites: the nitrogen of the quinoline and the nitrogen of the imine. The latter is more polarizable and thus, a softer base. Using a softer acid, such as Yb(OTf)_3_, the formation of the iminium ion is enhanced regioselectively at the nitrogen atom of the imine rather than that one of the 4-amino-7-chloroquinoline moiety. In contrast, acids with intermediate hardness, such as Sc(OTf)_3_ or indium (III) salts, tend to preferentially coordinate with the nitrogen of the quinoline, leading to lower yields.

Then, conditions for the cascade process (intermolecular *aza*-Diels-Alder cycloaddition/intramolecular *N*-acylation/decarboxylation/dehydration) between the 5-aminooxazole **14a** and maleic anhydride (**15**) were screened. The reaction was performed under μw irradiation using a mixture of PhMe and MeOH (9:2 *v*/*v*) as the solvent at temperatures of 70 °C and 80 °C (Table 2, Entries 1 and 2). However, the highest yield, 94%, was achieved when PhCl was used as the solvent at 80 °C (Table 2, Entry 3). It demonstrates that both procedures, UZ-3CR and cascade sequence, can be coupled into a full domino-type one-pot process without solvent-shifting.

#### 2.1.3. Synthesis of the Assayed Polyheterocycles

**Figure 1:**
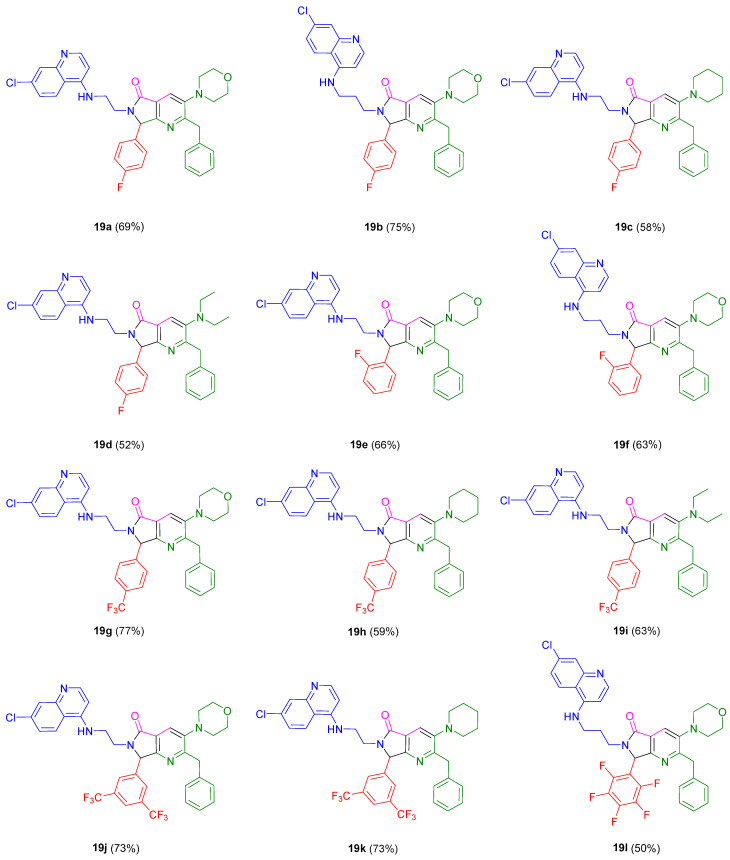
Synthesized and assayed fluorine-containing 4-amino-7-chloroquinoline-pyrrolo[3,4-*b*]pyridin-5-ones. Color key: red (aldehyde moiety), blue (amine moiety), green (isocyanide moiety) and pink (anhydride moiety).

Based on the previously obtained results, the synthesis of eleven additional fluorinated-pyrrolo[3,4-*b*]pyridin-5-ones **19b–l** containing the 4-amino-7-chloroquinoline moiety was carried out using the one-pot (MCR/cascade) approach. This one involved the use of three different α-isocyanoacetamides **5a–c**, two variants of aminoquinolines **8a–b** (varying the chain length), and five distinct fluorinated aromatic aldehydes **9a–e** (Figure 1). The yields obtained ranged from 50 to 77%, with atom economy values between 88 and 90%, typical of multi-component reactions, where most of the atoms from the reactants are present in the final products. Notably, during the synthesis of the pyrrolo[3,4-*b*]pyridin-5-ones **19a–l**, only two molecules of H_2_O and one of CO_2_ were released as by-products.

**Scheme 2:**
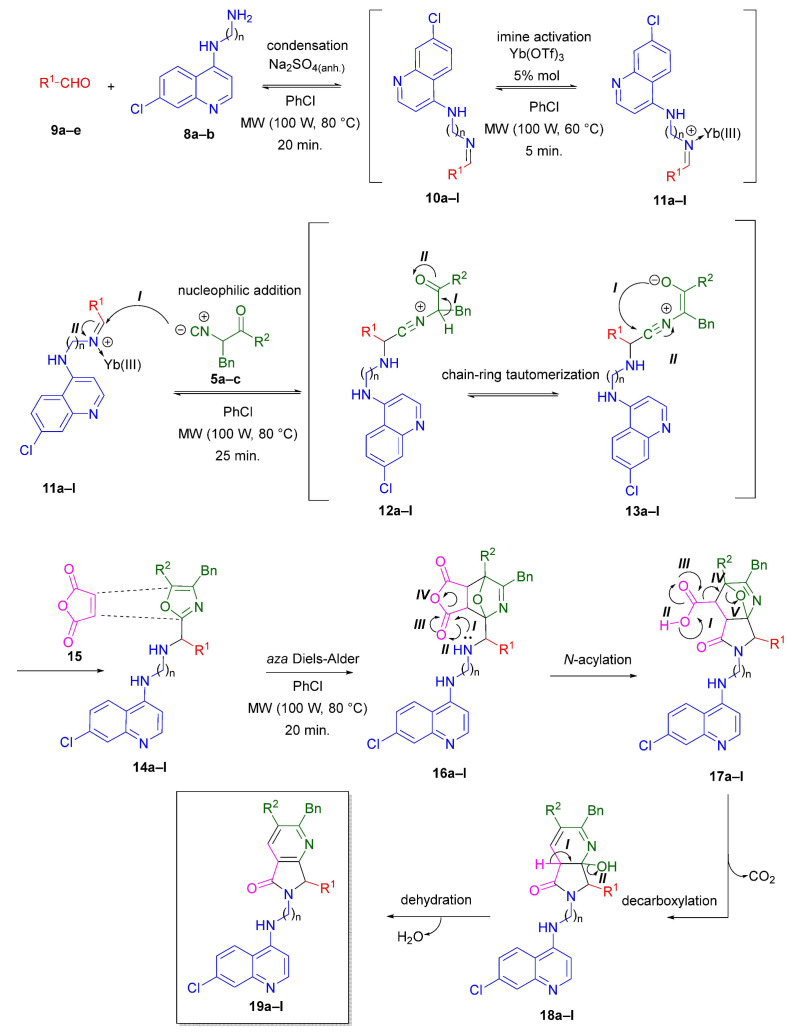
Plausible reaction mechanism to access to the polyheterocycles **19a–l**. Color key: red (aldehyde moiety), blue (amine moiety), green (isocyanide moiety) and pink (anhydride moiety).

The most plausible reaction mechanism that explains the synthesis of the fluorine-containing 4-amino-7-chloroquinoline-pyrrolo[3,4-*b*]pyridin-5-ones begins with the formation of the UZ-3CR product **14a** (Scheme 2). This process starts with the condensation between the aldehydes **9a–e** and amines **8a–b**, leading to the formation of imines **10a–l**. These imines are then converted into iminium cations **11a–l**, a crucial step facilitated by the Lewis acid Yb(OTf)_3_. Then, intermediates **11a–l** undergo a nucleophilic addition by the divalent carbon of the α-isocyanoacetamides **5a–c** forming nitrilium cations **12a–l**. These intermediates then quickly undergo a non-prototropic chain-ring tautomerization, yielding the 5-aminooxazoles **14a–l** as the Ugi-Zhu products. In the presence of maleic anhydride (**15**), the 5-aminooxazoles **14a–l** undergo an intermolecular [4+2] *aza*-Diels-Alder cycloaddition, forming the oxa-bridged intermediates **16a–l**. These intermediates undergo intramolecular *N*-acylations, followed by decarboxylation and dehydration, for ultimately forming the pyrrolo[3,4-*b*]pyridin-5-one polyheterocyclic core in the polyheterocycles **19a–l**. These compounds were characterized by spectroscopic techniques; see the ESM for further details (Figures S1–S72).

### 2.2. In Vitro Studies

#### 2.2.1. Cytotoxicity Assay

**Figure 2:**
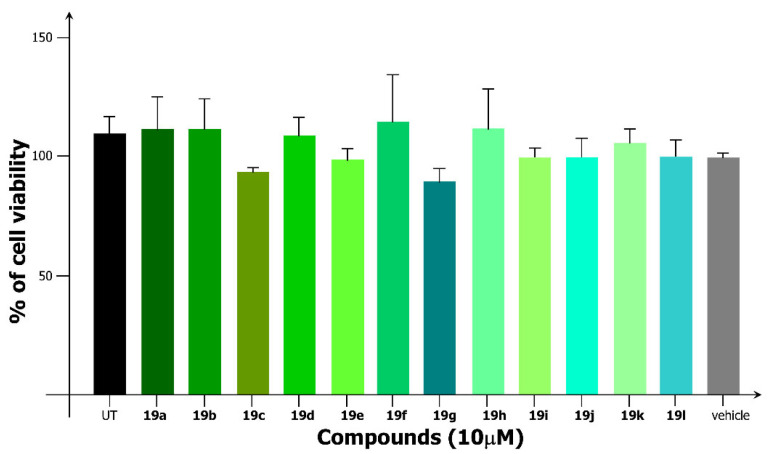
Cell viability assay. The experiment was conducted twice independently, each time in triplicate. Bars represent the standard deviation of the mean (n = 6); UT: Untreated; Vehicle: DMSO.

To evaluate the cytotoxic effects of polyheterocyclic products **19a–l**, Vero E6 cells were treated with increasing concentrations of the compounds to measure cell viability and to determine the optimal working concentration for subsequent studies. At 10 μM, cell viability remained above 90%, relative to vehicle control, except for compound **19g**, which maintained viability above 80% (Figure 2). The variability observed for some compounds, particularly **19f**, may be attributed to solubility limitations. Nevertheless, despite this variability, **19f** exhibited cell viability levels above 80%, indicating that its biological activity remained robust under the tested conditions. Based on these results, 10 μM was selected for the subsequent infection inhibition assays.

#### 2.2.2. Evaluation of Antiviral Activity

To evaluate the antiviral potential of the synthesized polyheterocyclic compounds, a plaque reduction assay was performed to quantify the number of infectious viral particles (plaque-forming units/mL, PFU/mL) in the presence of each compound. This classical virological method remains one of the most reliable tools for assessing compound-mediated inhibition of viral replication [33]. A time-of-addition assay was employed to explore the potential mechanism of action, testing compound efficacy when administered at two time points, at the moment of infection, which is referred to as 0 h (0 h), and 1 h (1 h) postinfection (pi).

**Figure 3:**
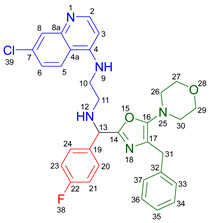
Inhibition of SARS CoV-2 infection. (**A**) Addition of compounds at the time of infection (0 h). (**B**) Addition of compounds 1 h postinfection. Plaque assay technique was conducted twice independently, each time in triplicate. Bars represent the standard deviation of the mean (n = 6). **** *p*-value < 0.0001, determined using ordinary one-way ANOVA; PFU/mL: Plaque-forming units/mL; UT: Untreated; Vehicle: DMSO.

At 0 h, a slight reduction in viral replication was observed for the compounds **19d**, **19g**, and **19l** compared to vehicle and untreated controls, with IC_50_ values of 6.74 µM, 7.50 µM, and 7.06 µM, respectively. In contrast, when administered at 1 h postinfection, compounds **19g** and **19i** exhibited a statistically significant decrease in viral titers relative to both control groups, with IC_50_ values of 6.98 µM and 5.29 µM, respectively (Figure 3 and Table 3). Notably, **19i** exhibited a plaque reduction of approximately one logarithmic unit in plaque formation. Thus, compounds **19d** and **19i** achieved the lowest IC_50_ values at 0 h and 1 h postinfection, respectively, indicating that **19d** may function as a potential prophylactic agent, while **19i** as a therapeutic agent.

Chloroquine, as a pharmacologically active compound, has been reported to exhibit antiviral activity against both SARS-CoV, with an IC_50_ of 8.8 µM [34], and SARS-CoV-2, with an IC_50_ of 1.13 µM [35]. It has also demonstrated efficacy when administered 3–5 h postinfection. The proposed mechanisms underlying its antiviral activity include the elevation of endosomal pH, thereby disrupting pH-dependent steps in viral endocytosis [36,37], and the inhibition of post-translational modifications of viral proteins via interference with viral protease activity, which requires an acidic environment [38]. Notably, in the case of viruses belonging to the *Flaviviridae* family, chloroquine has been shown to impair the proteolytic processing of viral proteins [39]. In the study, the conjugation of fluorinated-pyrrolo[3,4-*b*]pyridin-5-one with a 4-amino-7-chloroquinoline moiety preserved antiviral activity comparable to that of unmodified chloroquine fragment. Among the twelve synthesized compounds (**19a–l**), compounds **19d** and **19i** are distinguished by the presence of a diethylamine-derived fragment. Their diethylamine-derived side chain may contribute to enhanced intracellular distribution or improved interaction with viral or host targets. To gain further insight into the molecular basis of their selectivity and activity, in silico analyses, including molecular docking and theoretical calculations, were performed and described in the following sections.

### 2.3. In Silico Studies: (Docking and Molecular Dynamics)

#### 2.3.1. Introduction

This section systematically and succinctly delineates the principal findings derived from the assessment of compounds **19d** and **19i** as potential antiviral agents against SARS-CoV-2 infection at the time of infection 0 h or 1 h postinfection, respectively. The research concentrates on four critical viral proteins: M_pro_, helicase, replicase, and the spike protein, particularly its NTDα, NTDο, RBDα, and RBDο domains. To associate the affinity of these ligands with a potential mechanism of action, ADMETox characterization techniques were utilized to assess pharmacokinetic feasibility and toxicity, molecular docking methods were employed to investigate binding modes, and molecular dynamics (MDs) simulations were conducted to evaluate the progression of parameters such as RMSD, radius of gyration (Rgyr), and per-residue fluctuations (RMSF). The binding free energies were assessed using MM/GBSA, and a comprehensive 2D mapping of the protein–ligand interactions was conducted. Additionally, non-covalent interactions of these systems were analyzed using the quantum theory of atoms in molecules (QTAIM) and the non-covalent interactions index (NCI) to gain understanding from electronic structure methods of the interactions involved in the studied models. This approach helps us understand how **19d** and **19i** interact with different types of viruses on a deeper level. It also sets the stage for future experiments to confirm the results and improve the structure.

#### 2.3.2. ADMETox

The results showed that **19d** and **19i** (Table S1) are not expected to be well absorbed orally because they display very high lipophilicity (log P > 7) and a moderate polar surface area (TPSA ~61 Å^2^). This lipophilicity was linked to limited aqueous solubility, which shows that they need special formulations to make them more bioavailable. Both chemicals are unlikely to efficiently traverse the blood–brain barrier, which may be advantageous for central nervous system safety. Nonetheless, this necessitates vigilance for possible neurotoxic effects, especially for **19d**, which produced a value of 0.97 as per the ProTox-III model. In terms of metabolism, it is thought that **19i** interacts with the CYP3A4 and CYP2D6 isoenzymes as either a substrate or an inhibitor. On the other hand, **19d** is thought to be more closely linked to CYP2C9 and CYP2D6, with hepatic clearance taking place in both cases because they are lipophilic.

Regarding toxicity, the estimated median lethal dose (LD_50_ ~500 mg/kg) categorized **19d** and **19i** as Class 4. Nonetheless, some pertinent alarms were identified: **19d** demonstrates an elevated likelihood of mutagenicity, neurotoxicity, and possible immunotoxic consequences, whereas **19i** indicates cardiotoxicity linked to hERG II suppression. These results show that chemical structures could be improved to reduce risks. However, the absorption and permeability profiles show that the compounds could be useful for systemic use, as long as the right changes are made to limit negative effects.

#### 2.3.3. Cavity Search and Binding Site Validation

Machine learning algorithms, such as P2Rank and GRaSP, were used to find possible binding sites. PDBsum was also used for structural analysis (Tables S2 and S3). This method facilitated the discovery of several cavities, chosen based on empirical evidence and diverse inhibitory mechanisms documented in the literature [40]. There were two main areas found in M_pro_ (nsp5): the catalytic site and an allosteric site (S1). Both of these ones were thought to be good candidates for techniques that could stop the enzyme from working (Figure S73). In replicase (nsp7), important parts for RNA recognition and the binding interface with nsp8/nsp12 proteins were found (Figure S74). It was found that the polymerase (nsp12) needs certain areas to recognize RNA and nucleotides (Figure S75). These areas are important for genome extension and processing. The nucleotide binding site and the RNA interaction domain were also mapped out in helicase (nsp13) (Figure S76).

Cavities related to cellular recognition (NTD) and ACE2 binding (RBD) were discovered in the Spike protein of its Alpha (B.1.1.7) and Omicron (B.1.1.529) variants (Figure S77). Also, homology analysis (Figure S78) showed big changes in the NTD and RBD domains, which are close to these cavities. This suggests that the virus might have evolved in a way to change the affinity between its ligands and proteins. Despite these differences, docking experiments showed that compounds **19d** and **19i** have a high affinity and do not significantly change where they are expected to bind. The fact that the Alpha and Omicron variants are related in the NTD and RBD areas shows that the mutations are not mainly found in the known active sites. This means that they probably would not mess up the way the ligands in this study interact with each other. Nonetheless, they could affect characteristics like antigenicity or immune evasion.

#### 2.3.4. Docking Studies

The AD4, Vina, and Vinardo force fields were used to run a total of 42,000 docking simulations with ligands **19d** and **19i** in a wide range of defined cavities of interest. After using a redocking method and then clustering with a cutoff of 0.5 Å, Figure S79 (Bubble Plots) and Table S4 show the complexes that have the strongest affinity and the highest population densities. From Table S5, the results indicate that **19d** demonstrates exceptional affinity for the Spike protein, specifically in the *N*-terminal domain (NTD) and the receptor-binding domain (RBD) of the Alpha and Omicron versions. A binding score of −9.0 kcal/mol was achieved in the alpha variant of the N-terminal domain (NTDα), with approximately 33% of the population in the primary cluster, highlighting essential residues including ARG100, VAL124, PHE189, and LEU223, further supported by polar interactions with ARG100, HIS204, and MET174. It was found that NTDo had a binding score of about −8.5 kcal/mol and a cluster coverage of about 33%. TYR167 was also found to be a stabilizing factor. In RBDo, the binding energy is slightly less negative (−6.4 kcal/mol), but 86% of the molecules were found in the main cluster. This decrease was due to aromatic–polar interactions with TYR489, PHE490, and ARG493. This overview indicates a potential multi-site antiviral action of **19d**, particularly targeting the viral entry phase, due to the significant targets located within the Spike protein.

On the other hand, **19i** had a stronger affinity for non-structural proteins that are involved in SARS-CoV-2 replication and maturation. The allosteric site S1 in M_pro_ (nsp5) had a binding affinity of −8.2 kcal/mol and was occupied by about 38% of the protein. This suggests a non-competitive inhibitory mechanism that could make medicines that target the catalytic site work better. In replicase (nsp7), a binding score of −5.2 kcal/mol and 80% convergence point to a stable complex, even though its energy level is moderate. In this case, ASP245 and HIS246 are found to be important residues for catalysis. In the nucleotide recognition region of helicase (nsp13), a value of −8.6 kcal/mol (~29% cluster) was found, and ASP485, HIS554, and ASP534 all played a big role in binding.

The bubble plots in Figure S79 clearly show that the sites with the lowest binding scores (−9.0 to −8.6 kcal/mol) are mostly in important spots, like the NTD of Spike for **19d** and the helicase and allosteric site of M_pro_ for **19i**. The results show that **19d** might stop the virus from entering in places that can be changed easily, while **19i** would stop later stages of replication and genome processing. This duality is a good way to deal with mutational variants that affect different stages of the SARS-CoV-2 infection process. It makes advanced antiviral methods more flexible and effective. To investigate the stability and conformational dynamics of compounds **19d** and **19i** targeting critical SARS-CoV-2 sites, we performed 200 ns molecular dynamics (MDs) simulations in five independent replicas for each system. There was ligand **19d** attached to the Spike protein in its NTDα, NTDo, and RBDα regions, and ligand **19i** attached to M_pro_ (nsp5), replicase (nsp7), and helicase (nsp13). The complexes, which included the polymerase (nsp12) and RBDo, were thrown out because their docking energies were negative in the early studies.

During the paths, each complex showed us more about the overall stability, the flexibility in certain areas, and the ligand’s ability to change shape in its binding site. These findings facilitated the assessment of the persistence of each molecule’s docking under dynamic conditions and the protein’s response to the medication.

#### 2.3.5. Dynamics of the Complexes with **19d**

In the Spike protein, ligand **19d** exhibited persistent binding even in exposed regions susceptible to alterations; Table S6. Molecular dynamics simulations in the alpha variant of the *N*-terminal domain showed RMSD values ranging from around 1.5 to 4.0 Å for the domain and from 2.0 to 3.5 Å for the ligand, signifying prolonged docking stability. The gyration radius (Rg) was mostly between 20 and 21 Å, but some surface loops (residues 140–160 and 240–260) showed big changes in the RMSF that did not cause **19d** to separate. This tolerance to surface flexibility indicates a strong affinity in the *N*-terminal region of the Alpha version.

A comparable dynamic behavior was discovered in the NTDo (Omicron variant). The domain’s RMSD predominantly remained between 2 and 2–5 Å, with one replica nearing 5.0 Å in highly mobile areas, while still maintaining contact with **19d**, Table S6. The ligand ranged between around 2.5 and 4.5 Å, adjusting to loops associated with immune evasion. The Rg remained approximately 20–21 Å, showing that, despite the mutations accumulated in Omicron, the overall shape of the NTD is adequately conserved to stably admit **19d**.

In the RBDo, early docking scores suggested that ligand **19d** might have lower affinity, but the MD trajectories showed the opposite, Table S6. The protein demonstrated RMSD values of roughly 2–5 Å, with intermittent peaks of about 6 Å at the receptor binding region (binding ridge). The ligand fluctuated between approximately 2.5 and 5.0 Å without dissociation, but the Rg of the RBD consistently ranged from 19 to 21 Å, indicating a stable, compact structure. This discovery underscores the significance of dynamic stability in addition to the static affinity obtained via docking.

#### 2.3.6. Dynamics of the Complexes with **19i**

Overall, **19i** kept its ability to bind to the M_pro_ (nsp5, Figure S73) and replicase (nsp7, Figure S74) proteins, whose structures are very stable; Table S6. The system’s RMSD values were mostly between 1.5 and 5.0 Å in the complex with M_pro_. The radius of gyration (Rg) stayed between 25 and 26 Å, which showed that the catalytic fold was still there. Despite minor fluctuations in the ligand’s RMSD (2.0–4.5 Å), no indications of dissociation were observed, implying a stable docking. The RMSF analysis revealed that only the end of the protein exhibited enhanced mobility, leaving the active site, where **19i** is situated, unaffected.

Most replicas in replicase (nsp7) had a protein RMSD of about 1.5 to 3.0 Å, a consistent Rg of about 22.5 Å, and almost no atomic fluctuations; Table S6. This finding indicates that **19i** is positioned within a confined cavity, where restricted structural remodeling may enhance inhibitory efficacy. Conversely, in helicase (nsp13, Figure S76), a greater degree of flexibility and variability was noted. Certain replicas attained RMSD values of ~8.5 Å, while the Rg varied between 28.0 and 30.0 Å, indicating the considerable mobility of segments (residues 300–350 and 500–600) linked to ATPase activity and RNA binding. Still, **19i** stayed connected throughout the simulation, indicating a strong link that can adapt to nsp13′s flexibility and possibly manage temporary functional states.

The dynamic profiles elucidate the distinctions in the mechanisms of action between **19d** and **19i**. Indeed, the former (**19d**) demonstrates affinity for the Spike domains (NTD and RBD), even those exhibiting elevated mutation rates in the Alpha and Omicron versions, whereas the latter (**19i**) mainly associates with conserved non-structural proteins (nsp5, nsp7, nsp13). A clustering analysis with a cutoff radius of 3.0 Å was conducted in GROMACS to find out how common each conformation was. The NTDα/**19d** and NTDo/**19d** complexes stood out because they were present in more than 75% of the populations. These results emphasize the complementary nature of both compounds: **19d** obstructs viral entrance despite significant alterations, whereas **19i** inhibits intracellular replication. This dual antiviral strategy aims to combine the suppression of internal mechanisms with disruption of the Spike-receptor contact.

#### 2.3.7. Interaction of the NTDα/**19d** Complex

**Figure 4:**
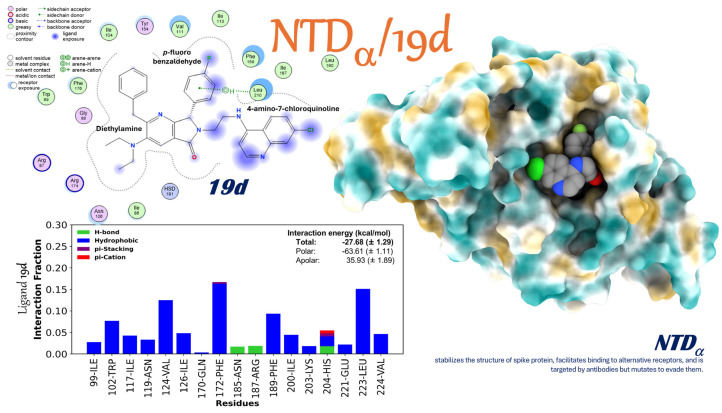
NTDα/**19d** Complex. The 2D picture in the upper left corner shows the chemical structure of **19d**. It shows the non-covalent interactions (hydrogen bonds, hydrophobic interactions, π–π interactions, etc.) between the Spike protein’s NTD residues and the Spike protein’s Alpha form. The histogram (bottom left) illustrates the contact fraction for each residue, prominently featuring Tyr102, Phe172, Gln170, and Leu223, among others. On the right, the NTDα surface is depicted, colored according to its hydrophobicity (blue: hydrophilic, orange: hydrophobic, white: neutral), with **19d** docking into a superficial cavity. The total energy value is −27.68 ± 1.29 kcal/mol, with −63.61 ± 1.11 kcal/mol polar and +35.93 ± 1.89 kcal/mol apolar. This shows a stable binding that may affect Spike’s extra function and the virus’s ability to hide from the immune system.

The complex comprising the NTDα region of the Spike protein and ligand **19d** (NTDα/**19d**: Figure 4, Supplementary Movie S1, Tables S7 and S8) displays a binding free energy of −27.68 ± 1.29 kcal/mol, indicating a thermodynamically robust and potentially useful interaction within the realm of viral biology. There is a positive overall energy balance, even though there is an apolar penalty of +35.93 ± 1.89 kcal/mol due to a desolvation process, which is mostly caused by a significant polar contribution (−63.61 ± 1.11 kcal/mol). This scenario indicates that the specificity and strength of the binding are predominantly dependent on clearly defined electrostatic interactions and hydrogen bonds.

Per-residue analysis identifies numerous essential amino acids for complex stabilization. The interactions between Phe172, Gln170, Leu223, Glu221, and Asn185 are made up of hydrogen bonds (mostly with Leu210), strong hydrophobic contacts, and sometimes π–π and π–cation interactions. These interactions enhance the ligand’s affinity and indicate that the placement of **19d** is ideally aligned with the architecture of the NTDα. Molecular dynamics analysis also supports these results: the RMSD and the radius of gyration of the NTDα stay very small during the simulation, which means that the domain’s overall structure is stable. The RMSF profile also shows that oscillations are mostly found in peripheral loops, especially between 140 and 160 and 240 and 260, but they do not affect the binding site’s integrity or cause **19d** to separate.

The Spike NTD is a region that often undergoes mutations in high-interest variants, which has considerable consequences for immune evasion. It looks like the stable binding of **19d** to this domain in the Alpha version means that the ligand might change how Spike does its extra tasks. Such actions may involve possible disruption of binding to gangliosides or other alternative receptors, which could aid in obstructing or alleviating viral immune evasion mechanisms. After putting all of these observations together, they support the idea that **19d** has a lot of potential as a regulator of Spike NTD function. It could be used to make antiviral drugs that target both viral entry and immune evasion.

#### 2.3.8. Interaction of the NTDo/**19d** Complex

**Figure 5:**
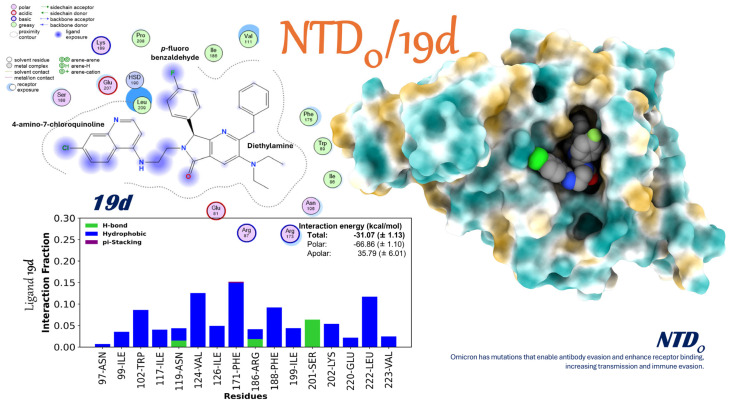
NTDo/**19d** Complex. A 2D picture (upper left) shows the chemical structure of **19d** and the non-covalent interactions (hydrogen bonds, hydrophobic interactions, π–π interactions, etc.) that it has with NTD residues in the Omicron version. The histogram (bottom left) illustrates the proportion of time each residue (e.g., Asn97, Ser98, Ile112, Tyr167, Arg186) maintains contact with the ligand. The right picture shows the NTDo surface. The colors show whether it is hydrophilic or hydrophobic (blue means hydrophilic, orange means hydrophobic, and white means neutral). This shows where **19d** fits into a shallow cavity. The energy breakdown (total: −31.07 ± 1.13 kcal/mol; polar: −66.86 ± 1.10 kcal/mol; apolar: +35.79 ± 6.01 kcal/mol) shows a strong interaction, which means that **19d** can bind even in areas that have been changed to avoid antibodies while improving its affinity for cellular receptors.

The study of the NTDo/**19d** complex (NTDo/**19d**: Figure 5, Supplementary Movie S1, Tables S9 and S10) shows that it has a stronger affinity (−31.07 ± 1.13 kcal/mol) than the Alpha form (−27.68 kcal/mol). The substantial polar component is thought to be responsible for the enhanced affinity (−66.86 ± 1.10 kcal/mol), which significantly surpasses the apolar penalty of +35.79 ± 6.01 kcal/mol. The per-residue energy analysis finds a large number of important stabilizers, such as Phe171, Ile126, Asn119, Glu220, Leu222, and Arg186. These help the molecules bind better through hydrophobic interactions and repeated hydrogen bonds, like with Ser201. Numerous amino acids demonstrate a significant proportion of contact (exceeding 0.15 on the occupancy scale), indicating a strong connection within a functionally pertinent cavity, despite the typical mutations associated with Omicron.

From a virological standpoint, it is notably significant because the ligand **19d** remains attached to a domain engineered to circumvent neutralizing antibodies by substituting or inhibiting residues in the NTD region. The fact that **19d** can accommodate itself in a space next to these epitopes without interfering with glycans or decreasing its effectiveness suggests a possible allosteric mechanism: the ligand may change the dom surface flexibility or how it interacts with cellular cofactors, in addition to competing with the ACE2 receptor. In cases where the Omicron variant shows strong immune resistance, these results suggest that **19d** is a strong pharmaceutical candidate that could help deal with the virus’s growing genetic diversity and offer an extra way to control it.

#### 2.3.9. Interactions of the M_pro_/**19i** Complex

**Figure 6:**
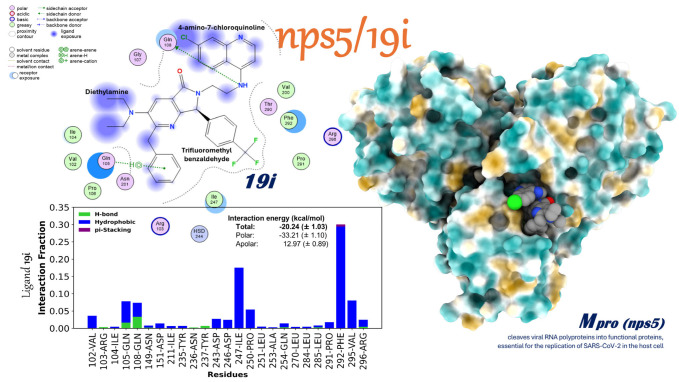
M_pro_/**19i** Complex. The top left corner of the 2D picture displays the chemical structure of **19i**. Some of the non-covalent interactions that it has with the residues of the major protease (M_pro_/nsp5) are shown in this structure. These include hydrogen bonds, hydrophobic interactions, π–π interactions, and more. The histogram (bottom left) illustrates the proportion of time each residue remains in contact with the ligand, emphasizing the significance of Phe292, Val295, Leu253, and Gln105, among others. The M_pro_ surface is shown on the right, with a color key that show hydrophobicity (blue = hydrophilic, orange = hydrophobic, and white = neutral). This diagram shows where **19i** is located inside the protein cavity. It is supported by the energy decomposition (−20.24 ± 1.03 kcal/mol total; −33.21 ± 1.10 kcal/mol polar; +12.97 ± 0.89 kcal/mol apolar), which indicates that **19i** stays bound. This phenomenon could stop the viral proteolysis that is needed for SARS-CoV-2 replication.

The examination of the interaction between the major protease of SARS-CoV-2 (M_pro_ or nsp5) and compound **19i** (M_pro_/**19i**: Figure 6, Supplementary Movie S2, Tables S11 and S12) indicates an average binding free energy of −20.24 ± 1.03 kcal/mol, as determined by MM/GBSA calculations. A strongly negative polar component (−33.21 ± 1.10 kcal/mol) and a positive apolar fraction (12.97 ± 0.89 kcal/mol) work together to make the overall thermodynamically favorable affinity. The numbers on this scale show how much desolvation (17.73 kcal/mol), a loss of non-polar surface area (−4.77 kcal/mol), and van der Waals energies (−35.71 kcal/mol) contributed. The net electrostatic contribution in vacuum is very small (2.50 kcal/mol), which shows that hydrophobic interactions are stronger than ionic interactions. The per-residue energy breakdown shows that Phe292 is the main stabilizer (−4.2 kcal/mol), mostly by stacking with **19i**’s aromatic ring in a π–π way. Together with Phe292, Val295 (−3.1 kcal/mol), Leu253 (−2.7 kcal/mol), and Phe140 (−2.5 kcal/mol) make up a hydrophobic core that surrounds the trifluoromethyl benzaldehyde part of the ligand. In the polar part, there are strong hydrogen bonds between the “chloroquine-like” amino group and the residues Gln105 (−1.8 kcal/mol) and Asn201 (−1.4 kcal/mol). There is also a halogen bond between Gln108 and the chlorine atom of the ligand. Even though they are not as strong as the hydrophobic interactions, these polar interactions make up 25–30% of the occupancy during the simulation, which shows how important they are for the stability of the binding. In line with this changing situation, the molecular dynamics simulations show a generally low RMSD, a steady radius of gyration, and only small changes in the RMSF profile, mostly at the ends of the proteins. During trajectory concatenation, it is seen that **19i** sometimes partially leaves the cavity and then comes back without affecting M_pro_’s overall compactness or integrity. Such behavior indicates a potential competitive inhibition mechanism, wherein **19i** securely binds to the active site without inducing significant structural changes. Moreover, the ligand **19i** seems like a good starting point for making inhibitors for highly conserved viral proteases because it binds strongly and does not change much.

### 2.4. In Silico Studies (Molecular Structure)

#### Quantum Chemistry Analysis of Non-Covalent Interactions

**Figure 7:**
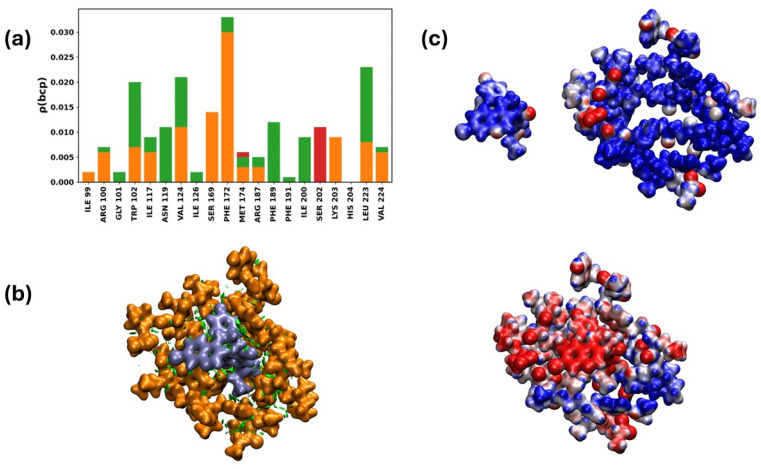
NTDα/**19d** Complex. (**a**) The upper left histogram of the QTAIM analysis for each residue shows the electron density involved in each interaction. Orange color indicates non-standard hydrogen bonds, green indicates H⋯H interactions, and red is associated with Lewis-type interactions. (**b**) NCI isosurfaces in green color (lower left) show dispersive interactions between ligand and receptor. (**c**) MEP for isolated systems and for the whole system. Negative values in red color and positive values in blue color.

**Figure 8:**
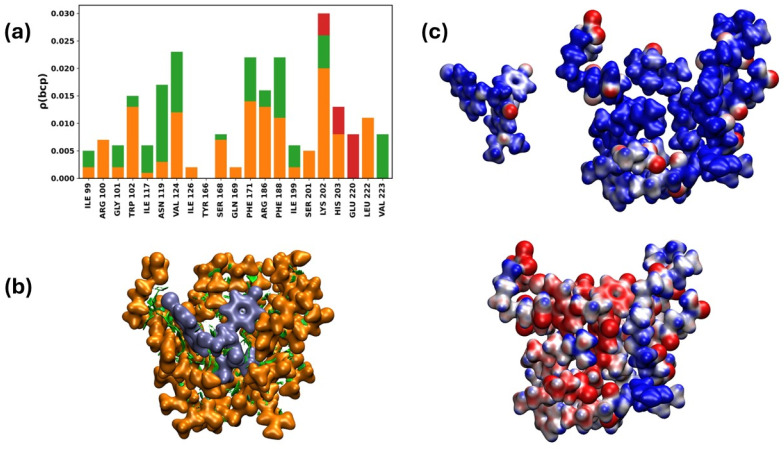
NTDo/**19d** Complex. (**a**) The upper left histogram of the QTAIM analysis for each residue shows the electron density involved in each interaction. Orange color indicates non-standard hydrogen bonds, green indicates H⋯H interactions, and red is associated with Lewis-type interactions. (**b**) NCI isosurfaces in green color (lower left) show dispersive interactions between ligand and receptor. (**c**) MEP for isolated systems and for the whole system. Negative values in red color and positive values in blue color.

**Figure 9:**
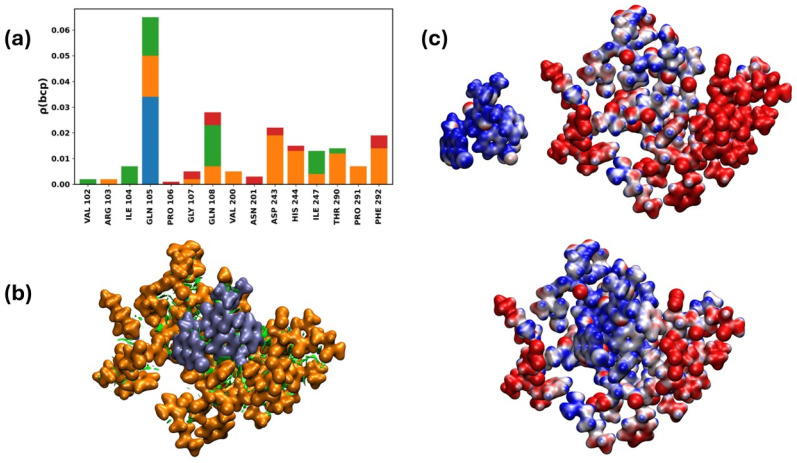
nsp5/**19i** Complex. (**a**) The upper left histogram of the QTAIM analysis for each residue shows the electron density involved in each interaction. Blue color indicates regular hydrogen bonds, orange indicates non-standard hydrogen bonds, green indicates H⋯H interactions, and red is associated with Lewis-type interactions. (**b**) NCI isosurfaces in green color (lower left) show dispersive interactions between ligand and receptor. (**c**) MEP for isolated systems and for the whole system. Negative values in red color and positive values in blue color.

The electron density evaluated at bond critical points between drug and protein is reported in Figure 7a, Figure 8a, and Figure 9a. In Figure 7a and Figure 8a, similar interactions are observed between those detected by QTAIM and MM/GBSA. The nature of the interactions is mainly unconventional hydrogen bonds (CH⋯X) and hydrogen–hydrogen interactions (H⋯H), which are identified as hydrophobic in MM/GBSA. In both methodologies, conventional hydrogen bonds are not relevant. However, QTAIM predicts interactions with other amino acids not detected by MM/GBSA; for example, ARG100, GLY101, SER169 in NTDα and SER168 in NTDo from both variants interact with ligand **19d** according to the QTAIM analysis. Another difference is that the predominance of interactions is predicted differently for some residues; for instance, in NTDo/**19d**, the relevance of LYS202 and LEU222 differs, while MM/GBSA predicts stronger interaction with LEU222, whereas QTAIM indicates a stronger interaction with LYS202, and vice versa. Same result is observed for NTDα/**19d**. The NCI isosurfaces (Figure 7b, Figure 8b and Figure 9b) corroborate the dispersive nature of most interactions (green isosurfaces), including van der Waals and some non-conventional hydrogen bonds. Thus, there are directional non-covalent interactions although non-directional interactions are also observed in these systems.

Figure 7c and Figure 8c show the Molecular Electrostatic Potential (MEP) of ligand **19d** and the cavity of NTDα and NTDo, respectively. In both variants, a redistribution of charge is observed when the ligand interacts with the cavity. In the isolated species, most regions show electron deficiency (blue regions), whereas upon interaction, regions with higher charge density (red regions) become apparent.

The case of nps5/**19i** (Figure 9a) is different from the previous ones. Here, the MM/GBSA methodology predicts a greater number of interacting residues, although each one contributes only a small fraction. In this approach, the most significant interactions are with PHE292 and ILE247, which are hydrophobic in nature. In contrast, although QTAIM also detects interactions with those same residues, the most important interaction between **19i** and nps5 is with residue GLN105; additionally, conventional hydrogen bonds are just as relevant as CH⋯X and H⋯H interactions, which are classified as hydrophobic by MM/GBSA. Another major difference between the MM/GBSA and QTAIM predictions is that in the latter, interaction with residue HIS244 is also significant, whereas it is not detected by the MM/GBSA method.

For the MEP of the isolated species nps5 and **19i** (Figure 9c), it is observed that, unlike in previous cases, the receptor cavity shows regions with a greater contribution of negative charge that, upon interacting with the ligand, become areas with a charge deficiency. This suggests that there is a charge transfer from the cavity to the ligand, which appears in a region where electronic density accumulates.

The findings from the NTD/**19d** and M_pro_/**19i** investigations delineate two complementary strategies for suppressing SARS-CoV-2:

1. Changing exposed and changeable domains: **19d** shows a lot of adaptive flexibility when binding to the NTD’s polar regions in both the Alpha and Omicron versions. The strength of this binding suggests that there may be an allosteric or structural disruption that could make it harder for the virus to enter and avoid the immune response of the host.
2. Stopping viral replication in targets that have been around for a long time: **19i** interacts with the active site of M_pro_, an important and highly conserved enzyme, through a network of contacts mainly made up of hydrophobic forces and stable hydrogen bonds. This method, which is similar to competitive inhibition, stops the viral proteolysis and may still work even if changes are made to other parts of the genome.

The coexistence of these two methodologies exemplifies a dual antiviral strategy: **19d** impedes or alters the accessory roles of the Spike protein, which are particularly significant during the cell recognition phase, while **19i** directly inhibits the proteolytic function of M_pro_. This duality may make it more effective and more resistant to changes brought about by evolution; variants that partially avoid one mechanism might still be vulnerable to the other. Because of this, **19d** and **19i** are very complementary molecular frameworks that, if improved, could make it easier to make next-generation drugs that target different stages of the SARS-CoV-2 infection cycle.

## 3. Materials and Methods

### 3.1. Chemistry (Software, Instrumentation and Chemicals)

Using the Bruker AMX Advance III spectrometer (500 MHz, Fällande, Uster, Switzerland), the ^1^H and ^13^C nuclear magnetic resonance (NMR) spectra were acquired and processed. The solvents used for NMR experiments were deuterated chloroform (CDCl_3_) and deuterated dimethyl sulfoxide (DMSO-*d*_6_). Chemical shifts are reported in parts per million (ppm). Coupling constants are reported in Hertz (*J*/Hz), with tetramethylsilane (TMS) used as the internal reference for NMR spectra at 0.00 ppm. The multiplicities of the signals are denoted using standard abbreviations: singlet (s), doublet (d), triplet (t), quartet (q), and multiplet (m). NMR spectra were analyzed using MestReNova software (Version 12.0.0-20080, A Coruña, Spain). The infrared (IR) spectrum was obtained using a Perkin-Elmer 2000 spectrometer (Norwalk, CT, USA) through the attenuated total reflectance (ATR) method. Maximum absorbance peaks are reported in reciprocal centimeters (υ_max_/cm^−1^), and these measurements are uncorrected. The IR spectrum was analyzed with Origin software (Version 2018b, 9.55, OriginLab Corporation, Northampton, MA, USA). High-resolution mass spectroscopy (HRMS) was performed by electrospray ionization (ESI) using a Micro-TOF II spectrometer from Bruker Daltonics GmbH (Bremen, Germany). The sample was injected directly via the Apollo source and analyzed using the time-of-flight (TOF) method. The HRMS spectrum was further analyzed with Compass software (Version 1.5, Bruker Daltonik GmbH, Bremen, Germany). Microwave-assisted reactions were conducted in closed-vessel mode using a CEM Discover SPMW reactor (Matthews, NC, USA). Reaction progress was monitored by thin-layer chromatography (TLC), with spots visualized under ultraviolet (UV) light at either 254 or 365 nm. Glass preparative plates (20 × 20 cm^2^) coated with silica gel 60, which was doped with a UV indicator (F_254_), were used for product purification. All starting reagents and solvents were used as received, without further purification, distillation, or dehydration. Chemical structures were drawn using ChemDraw software (Version 15.0.0.106 Professional, Perkin Elmer Informatics, Cambridge, MA, USA). The purity of all synthesized products was assessed by NMR and was greater than 96%.

### 3.2. General Procedure for the Synthesis of the Fluorinated 4-Amino-7-chloroquinoline-5-aminoxazole ***14a*** and fluorinated 4-amino-7-chloroquinoline-pyrrolo[3,4-b]pyridin-5-ones ***19a–l***

In a 10 mL CEM Discover microwave reaction tube equipped with a magnetic stirring bar, 2.0 mL of PhCl were added along with anhydrous sodium sulfate (Na_2_SO_4_) (1.0 equiv.). Then, the corresponding fluorinated aromatic aldehyde (1.0 equiv.) and the corresponding aminoquinoline (1.0 equiv.) were sequentially added. The reaction mixture was stirred at room temperature for 10 min, then heated to 80 °C using microwaves for 25 min. Next, Ytterbium (III) triflate (Yb(OTf)_3_) (5% mol) was added and the reaction continued at 65 °C for 5 min. Afterward, the corresponding α-isocyanoacetamide (1.2 equiv.) was added and the reaction mixture heated for 30 min at 80 °C. Finally, maleic anhydride (1.2 equiv.) was added, and the reaction was heated at 80 °C for an additional time of 25 min using microwaves. After the reaction finished, the solvent was removed to dryness. Then, liquid–liquid extraction and the crude purification were performed by silica gel column chromatography, followed by preparative thin-layer chromatography (TLC). This process yields the corresponding fluorinated 4-amino-7-chloroquinoline-pyrrolo[3,4-*b*]pyridin-5-ones (**19a–l**) as white solids.

#### 3.2.1. *N*^1^-((4-Benzyl-5-morpholinooxazol-2-yl)(4-fluorophenyl)methyl)-*N*^2^-(7-chloroquinolin-4-yl)ethane-1,2-diamine **14a**

According to the general procedure, the following compounds were added sequentially to a PhCl (2.0 mL): 4-fluorobenzaldehyde (0.062 g, 0.5 mmol), *N*-(7-chloroquinolin-4-yl)ethane-1,2-diamine (0.111 g, 0.5 mmol), anhydrous sodium sulfate (Na_2_SO_4_) (0.071 g, 0.5 mmol), ytterbium (III) triflate (Yb(OTf)_3_) (0.024 g, 0.04 mmol), and the 2-isocyano-1-morpholino-3-phenylpropan-1-one (0.146 g, 0.6 mmol). This reaction resulted in the formation of compound **14a**, which was obtained as a slightly yellow resin (39% yield); ^1^H-NMR (500 MHz, CDCl_3_): 8.45 (d, 1H, *J* = 5.5 Hz, H-2), 7.93 (d, 1H, *J* = 2.2 Hz, H-8), 7.87–7.81 (d, 1H, *J* = 8.9 Hz, H-5), 7.41–7.37 (m, 2H, H-24, H-20), 7.25–7.14 (m, 6H, H-6, H-33, H-34, H-35, H-36, H-37), 7.06–7.01 (m, 2H, H-23, H-21), 6.42 (bs, NH), 6.28 (d, 1H, *J* = 5.5 Hz, H-3), 4.92 (s, 1H, H-13), 3.84 (s, 2H, H-31), 3.67–3.64 (m, 4H, H-29, H-27), 3.33–3.28 (m, 2H, H-10), 3.12–3.01 (m, 2H, H-11), 2.91–2.85 (m, 4H, H-30, H-26); ^13^C-NMR (125 MHz, CDCl_3_): 163.5 (C-22), 161.5 (C-4), 158.5 (C-14), 152.6 (C-8a), 151.0 (C-2), 150.5 (C-7), 148 (C-19), 139.2 (C-32), 135.0 (C-16), 129.2 (d, ^3^*J* = 8.2 Hz, C-20, C-24), 128.4 (C-34), 128.4 (C-36), 128.3 (C-33, C-37), 127.6 (C-8), 126.3 (C-35), 125.4 (C-6), 124.3 (C-17), 121.9 (C-5), 117.2 (C-4a), 115.6 (d, ^2^*J* = 26.6 Hz, C-21, C-23), 98.6 (C-3), 66.7 (C-27), 66.7 (C-29), 60.3 (C-13), 50.8 (C-26), 50.8 (C-30), 45.5 (C-11), 42.6 (C-10), 31.7 (C-31).


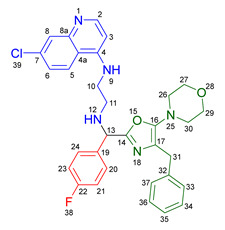


#### 3.2.2. 2-Benzyl-6-(2-((7-chloroquinolin-4-yl)amino)ethyl)-7-(4-fluorophenyl)-3-morpholino-6,7-dihydro-5*H*-pyrrolo[3,4-*b*]pyridin-5-one **19a**

According to the general procedure, the following compounds were added sequentially to a PhCl (2.0 mL): 4-fluorobenzaldehyde (0.062 g, 0.5 mmol), *N*-(7-chloroquinolin-4-yl)ethane-1,2-diamine (0.111 g, 0.5 mmol), anhydrous sodium sulfate (Na_2_SO_4_) (0.071 g, 0.5 mmol), ytterbium (III) triflate (Yb(OTf)_3_) (0.024 g, 0.04 mmol), 2-isocyano-1-morpholino-3-phenylpropan-1-one (0.146 g, 0.6 mmol), and maleic anhydride (0.059 g, 0.6 mmol). This reaction resulted in the formation of compound **19a**, which was obtained as a foamy white solid weighing 0.208 g (69% yield). R*_f_* = 0.41 (AcOEt-MeOH = 90/5, *v*/*v*); ^1^H-NMR (500 MHz, CDCl_3_): 8.47 (d, 1H, *J* = 5.4 Hz, H-34), 7.92 (d, 1H, *J* = 2.1Hz, H-37), 7.90 (s, 1H, H-15), 7.85 (d, 1H, *J* = 9.1 Hz, H-40), 7.42 (dd, 1H, *J* = 8.9, 2.2 Hz, H-39), 7.17–7.08 (m, 7H, H-18, H-19, H-20, H-21, H-22, H-24, H-28), 7.05 (dd, 2H, *J* = 8.9, 8.4 Hz, H-25, H-27), 6.71(s, 1H, H-31), 6.17 (d, 1H, *J* = 5.4 Hz, H-33), 5.51 (s, 1H, H-11), 4.27 (d, 1H, *J* = 14.0 Hz, H-16), 4.20 (d, 1H, *J* = 14.0 Hz, H-16′), 4.14–4.05 (m, 1H, H-30), 3.80 (t, 4H, *J* = 4.6 Hz, H-2, H-6), 3.77–3.701 (m, 1H, H-30′), 3.43–3.36 (m, 1H, H-29), 3.26–3.18 (m, 1H, H-29′), 2.88–2.80 (m, 4H, H-3, H-5); ^13^C-NMR (125 MHz, CDCl_3_): 169.7 (C-13), 163.0 (d, ^1^*J* = 247.8 Hz, C-26), 162.9 (C-8), 160.0 (C-10), 151.7 (C-34), 149.9 (C-32), 148.9 (C-36), 148.2 (C-7), 138.9 (C-17), 135.6 (C-38), 130.8 (C-23), 129.8 (d, ^3^*J* = 8.4 Hz, C-24, C-28), 128.7 (C-18, C-22), 128.4 (C-37), 128.2 (C-19, C-21), 126.2 (C-20), 125.6 (C-39), 123.7 (C-15), 123.2 (C-14), 122.0 (C-40), 117.1 (C-41), 116.2 (d, ^2^*J* = 21.8 Hz, C-25, C-27), 98.2 (C-33), 67.0 (C-2, C-6), 66.2 (C-11), 53.0 (C-3, C-5), 44.0 (C-29), 40.5 (C-30), 40.1 (C-16); HRMS: (ESI^+^) *m/z* calcd. for [M-H]^+^ C_35_H_32_ ClFN_5_O_2_^+^ 608.2223, found 608.2206 (error = 2.8 ppm); IR (*ν*, cm^−1^): 1687 (C=O), 1578 (C=C), 1441 (C-C), 1218 (C=C), 1114 (C-F), 996, 754 (C-C).


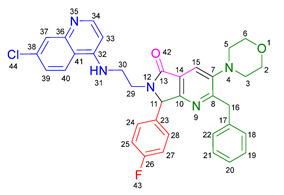


#### 3.2.3. 2-Benzyl-6-(3-((7-chloroquinolin-4-yl)amino)propyl)-7-(4-fluorophenyl)-3-morpholino-6,7-dihydro-5*H*-pyrrolo[3,4-*b*]pyridin-5-one **19b**

According to the general procedure, the following compounds were added sequentially to a PhCl (2.0 mL): 4-fluorobenzaldehyde (0.062 g, 0.5 mmol), *N^1^*-(7-chloroquinolin-4-yl)propane-1,3-diamine (0.117 g, 0.5 mmol), anhydrous sodium sulfate (Na_2_SO_4_) (0.071 g, 0.5 mmol), ytterbium (III) triflate (Yb(OTf)_3_) (0.024 g, 0.04 mmol), 2-isocyano-1-morpholino-3-phenylpropan-1-one (0.146 g, 0.6 mmol), and maleic anhydride (0.059 g, 0.6 mmol). This reaction resulted in the formation of compound **19b**, which was obtained as a foamy white solid weighing 0.233g (75% yield). R*_f_* = 0.44 (AcOEt-MeOH = 90/5, *v*/*v*); ^1^H-NMR (500 MHz, CDCl_3_): 8.45 (d, 1H, *J* = 5.4 Hz, H-35), 7.98 (d, 1H, *J* = 9.0 Hz, H-41), 7.92–7.90 (m, 2H, H-15, H-38), 7.37 (dd, 1H, *J* = 8.9, 2.2 Hz, H-40), 7.20–7.08 (m, 7H, H-18, H-19, H-20, H-21, H-22, H-24, H-28), 7.06–7.02 (m, 2H, H-25, H-27), 6.63 (t, 1H, *J* = 6.3 Hz, H-32), 6.32 (d, 1H, *J* = 5.4 Hz, H-34), 5.43 (s, 1H, H-11), 4.30 (d, 1H, *J* = 14.0 Hz, H-16), 4.22 (d, 1H, *J* = 14.0 Hz, H-16′), 3.93–3.86 (m, 1H, H-29), 3.82 (t, 4H, *J* = 4.6 Hz, H-2, H-6), 3.49–3.39 (m, 1H, H-31), 3.30–3.21 (m, 2H, H-31′, H-29′), 2.89–2.81 (m, 4H, H-3, H-5), 1.82–1.66 (m, 2H, H-30); ^13^C-NMR (125 MHz, CDCl_3_): 168.4 (C-13), 162.8 (d, ^1^*J* = 247.3 Hz, C-26), 162.0 (C-8), 160.0 (C-10), 151.8 (C-35), 149.7 (C-33), 149.3 (C-37), 148.2 (C-7), 139.0 (C-17), 134.9 (C-39), 131.0 (C-23), 129.7 (d, ^3^*J* = 9.9 Hz, C-24, C-28), 128.7 (C-18, C-22), 128.4 (C-38), 128.2 (C-19, C-20), 126.3 (C-20), 125.3 (C-40), 123.7 (C-15), 123.5 (C-14), 121.9 (C-41), 117.6 (C-42), 116.2 (d, ^2^*J* = 26.2 Hz, C-25, C-27), 98.4 (C-34), 67.1 (C-2, C-6), 65.3 (C-11), 53.0 (C-3, C-5), 40.0 (C-16), 39.0 (C-29), 37.7 (C-31), 26.0 (C-30); HRMS: (ESI^+^) *m/z* calcd. for [M-H]^+^ C_36_H_34_ ClFN_5_O_2_^+^ 622.2380, found 622.2393 (error = 2.1 ppm); IR (*ν*, cm^−1^): 1674 (C=O), 1584 (C=C), 1443 (C-C), 1107, 855 (C-F).


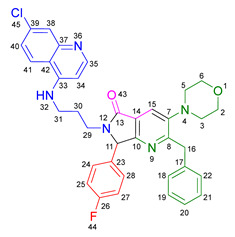


#### 3.2.4. 2-Benzyl-6-(2-((7-chloroquinolin-4-yl)amino)ethyl)-7-(4-fluorophenyl)-3-(piperidin-1-yl)-6,7-dihydro-5*H*-pyrrolo[3,4-*b*]pyridin-5-one **19c**

According to the general procedure, the following compounds were added sequentially to a PhCl (2.0 mL): 4-fluorobenzaldehyde (0.062 g, 0.5 mmol), *N*-(7-chloroquinolin-4-yl)ethane-1,2-diamine (0.111 g, 0.5 mmol), anhydrous sodium sulfate (Na_2_SO_4_) (0.071 g, 0.5 mmol), ytterbium (III) triflate (Yb(OTf)_3_) (0.024 g, 0.04 mmol), 2-isocyano-3-phenyl-1-(piperidin-1-yl)propan-1-one (0.145, 0.6 mmol), and maleic anhydride (0.059 g, 0.6 mmol). This reaction resulted in the formation of compound **19c**, which was obtained as a foamy white solid weighing 0.175 g (58% yield). R*_f_* = 0.47 (AcOEt-MeOH = 90/5, *v*/*v*); ^1^H-NMR (500 MHz, CDCl_3_): 8.45 (d, 1H, *J* = 5.3 Hz, H-15), 7.91 (d, 1H, *J* = 2.2 Hz, H-18), 7.85 (d, 1H, *J* = 8.9 Hz, H-21), 7.85 (s, 1H, H-7), 7.39 (dd, 1H, *J* = 8.9, 2.2 Hz, H-20), 7.15–7.08 (m, 7H, H-24, H-28, H-31, H-32, H-33, H-34, H-35), 7.04–7.00 (m, 2H, H-25, H-27), 6.76 (s, 1H, H-12), 6.14 (d, 1H, *J* = 5.4 Hz, H-14), 5.47 (s, 1H, H-2), 4.22 (d, 1H, *J* = 13.8 Hz, H-29), 4.16 (d, 1H, *J* = 13.8 Hz, H-29′), 4.09–4.02 (m, 1H, H-10), 3.73–3.66 (m, 1H, H-10′), 3.39–3.33 (m, 1H, H-11), 3.22–3.15 (m, 1H, H-11′), 2.80–2.76 (m, 4H, H-37, H-41), 1.72–1.67 (m, 4H, H-38, H-40), 1.60–1.55 (m, 2H, H-39); ^13^C-NMR (125 MHz, CDCl_3_): 169.9 (C-9), 162.9 (d, ^1^*J* = 249.1 Hz, C-26), 162.8 (C-5), 159.1 (C-3), 151.8 (C-15), 149.9 (C-17), 149.7 (C-6), 1478.9 (C-13), 139.2 (C-30), 134.9 (C-19), 131.0 (C-23), 129.7 (d, ^3^*J* = 3.8 Hz, C-24, C-28), 128.7 (C-31, C-35), 128.3 (C-18), 128.0 (C-32, C-34), 126.0 (C-33), 125.5 (C-20), 123.0 (C-7), 122.9 (C-8), 122.0 (C-21), 11.72 (C-22), 116.11 (d, ^2^*J* = 21.8 Hz, C-25, C-27), 98.2 (C-14), 66.1 (C-2), 54.2 (C-37, C-41), 44.0 (C-11), 40.4 (C-10), 39.8 (C-29), 26.3 (C-38, C-40), 23.8 (C-39); HRMS: (ESI^+^) *m/z* calcd. for [M-H]^+^ C_36_H_34_ ClFN_5_O^+^ 606.2430, found 606.2408 (error = 3.7 ppm); IR (*ν*, cm^−1^): 1681 (C=O), 1584 (C=C), 1443 (C-C), 1380 (C=N), 1304, 1212 (C-N), 846 (C-C), 813, 743 (C-F).


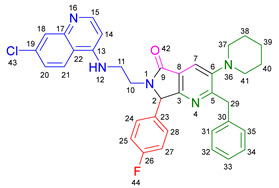


#### 3.2.5. 2-Benzyl-6-(2-((7-chloroquinolin-4-yl)amino)ethyl)-3-(diethylamino)-7-(4-fluorophenyl)-6,7-dihydro-5*H*-pyrrolo[3,4-*b*]pyridin-5-one **19d**

According to the general procedure, the following compounds were added sequentially to a PhCl (2.0 mL): 4-fluorobenzaldehyde (0.062 g, 0.5 mmol), *N*-(7-chloroquinolin-4-yl)ethane-1,2-diamine (0.111 g, 0.5 mmol), anhydrous sodium sulfate (Na_2_SO_4_) (0.071 g, 0.5 mmol), ytterbium (III) triflate (Yb(OTf)_3_) (0.024 g, 0.04 mmol), *N*,*N*-diethyl-2-isocyano-3-phenylpropanamide (0.138 g, 0.6 mmol), and maleic anhydride (0.059 g, 0.6 mmol). This reaction resulted in the formation of compound **19d**, which was obtained as a foamy white solid weighing 0.154 g (52% yield). R*_f_* = 0.49 (AcOEt-MeOH = 90/5, *v*/*v*); ^1^H-NMR (500 MHz, CDCl_3_): 8.47 (d, 1H, *J* = 5.4 Hz, H-15), 7.96–7.92 (m, 1H, H-18), 7.88–7.86 (m, 2H, H-7, H-21), 7.41 (dd, 1H, *J* = 8.9, 2.2 Hz, H-20), 7.17–7.04 (m, 9H, H-31, H-32, H-33, H-34, H-35, H-24, H-28, H-25, H-27), 6.86 (s, 1H, H-12), 6.17 (d, 1H, *J* = 5.5 Hz, H-14), 5.50 (s, 1H, H-2), 4.26 (d, 1H, *J* = 14.0 Hz, H-29), 4.18 (d, 1H, *J* = 14.0 Hz, H-29′), 4.10–4.04 (m, 1H, H-10), 3.77–3.69 (m, 1H, H-10′), 3.43–3.35 (m, 1H, H-11), 3.25–3.18 (m, 1H, H-11′), 3.01–2.95 (m, 4H, H-37, H-42), 0.91 (m, 6H, H-38, H-43); ^13^C-NMR (125 MHz, CDCl_3_): 170.0 (C-9), 164.3 (C-5), 163.0 (d, ^1^*J* = 248.5 Hz, C-26), 159.3 (C-3), 151.5 (C-15), 150.1 (C-13), 147.0 (C-6), 139.2 (C-17, C-30), 135.1 (C-19), 130.9 (C-23), 129.7 (d, ^3^*J* = 8.3 Hz, C-24, C-28), 128.8 (C-31, C-35), 127.9 (C-32, C-34), 125.9 (C-33), 125.6 (C-18, C-40), 125.4 (C-20), 122.7 (C-8), 122.1 (C-7), 117.1 (C-22), 116.3 (d, ^2^*J* = 25.3 Hz, C-25, C-27), 98.2 (C-14), 66.3 (C-2), 47.6 (C-37, C-42), 44.0 (C-11), 40.5 (C-10), 39.9 (C-29), 12.0 (C-38, C-43); HRMS: (ESI^+^) *m/z* calcd. for [M-H]^+^ C_35_H_34_ ClFN_5_O^+^ 594.2430, found 594.2423 (error = 1.2 ppm); IR (*ν*, cm^−1^): 1679 (C=O), 1583 (C=C), 1442 (C-C), 1382 (C=N), 1217 (C=C), 1155 (C-F).


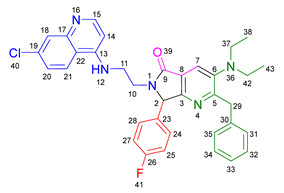


#### 3.2.6. 2-Benzyl-6-(2-((7-chloroquinolin-4-yl)amino)ethyl)-7-(2-fluorophenyl)-3-morpholino-6,7-dihydro-5*H*-pyrrolo[3,4-*b*]pyridin-5-one **19e**

According to the general procedure, the following compounds were added sequentially to a PhCl (2.0 mL): 2-fluorobenzaldehyde (0.062 g, 0.5 mmol), *N*-(7-chloroquinolin-4-yl)ethane-1,2-diamine (0.111 g, 0.5 mmol), anhydrous sodium sulfate (Na_2_SO_4_) (0.071 g, 0.5 mmol), ytterbium (III) triflate (Yb(OTf)_3_) (0.024 g, 0.04 mmol), 2-isocyano-1-morpholino-3-phenylpropan-1-one (0.146 g, 0.6 mmol), and maleic anhydride (0.059 g, 0.6 mmol). This reaction resulted in the formation of compound **19e**, which was obtained as a foamy white solid weighing 0.199 g (66% yield). R*_f_* = 0.40 (AcOEt-MeOH = 90/5, *v/v*); ^1^H-NMR (500 MHz, CDCl_3_): 8.45 (d, 1H, *J* = 5.3 Hz, H-34), 7.92 (s, 1H, H-15), 7.91 (d, 1H, *J* = 2.1 Hz, H-37), 7.83 (d, 1H, *J* = 8.7 Hz, H-40), 7.40 (dd, 1H, *J* = 8.9, 2.2 Hz, H-39), 7.38–7.34 (m, 1H, H-26), 7.21–7.05 (m, 7H, H-18, H-19, H-20, H-21, H-22, H-25, H-28), 6.87(m, 1H, H-27), 6.65–6.59 (m, 1H, H-31), 6.16 (d, 1H, *J* = 5.4 Hz, H-33), 5.92 (s, 1H, H-11), 4.27 (d, 1H, *J* = 13.8 Hz, H-16), 4.24 (d, 1H, *J* = 13.8 Hz, H-16′), 4.12–4.07 (m, 1H, H-29), 3.83–3.79 (m, 4H, H-2, H-6), 3.75–3.69 (m, 1H, H-29′), 3.46–3.40 (m, 1H, H-30), 3.24–3.18 (m, 1H, H-30′), 2.86–2.81 (m, 4H, H-3, H-5); ^13^C-NMR (125 MHz, CDCl_3_): 169.5 (C-13), 162.7 (C-8), 161.7 (d, ^1^*J* = 248.8 Hz, C-24), 162.7 (C-8), 159.4 (C-10), 151.8 (C-34), 149.8 (C-32), 149.0 (C-36), 148.2 (C-7), 139.0 (C-17), 134.8 (C-38), 130.9 (d, ^3^*J* = 8.4 Hz, C-26), 129.5 (C-27), 128.6 (C-18, C-22), 128.4 (C-37), 128.1 (C-19, C-21), 126.2 (C-20), 125.5 (C-39), 124.8 (d, ^3^*J* = 3.5 Hz, C-28), 124.0 (C-14), 123.7 (C-15), 122.3 (d, ^2^*J* = 12.5 Hz, C-23), 121.9 (C-40), 117.2 (C-41), 116.2 (d, ^2^*J* = 21.5Hz, C-25), 98.2(C-33), 67.0 (C-2, C-6), 60.2 (C-11), 53.0 (C-3, C-5), 43.8 (C-30), 40.5 (C-29), 40.0 (C-16); HRMS: (ESI^+^) *m/z* calcd. for [M-H]^+^ C_35_H_32_ ClFN_5_O_2_^+^ 608.2223, found 608.2231 (error = 1.3 ppm); IR (*ν*, cm^−1^): 1679 (C=O), 1578 (C=C), 1444 (C-C), 1323 (C=N), 1112 (C-F), 1062 (C-O-C), 750 (C-C).


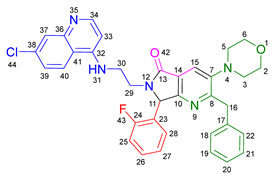


#### 3.2.7. 2-Benzyl-6-(3-((7-chloroquinolin-4-yl)amino)propyl)-7-(2-fluorophenyl)-3-morpholino-6,7-dihydro-5*H*-pyrrolo[3,4-*b*]pyridin-5-one **19f**

According to the general procedure, the following compounds were added sequentially to a PhCl (2.0 mL): 2-fluorobenzaldehyde (0.062 g, 0.5 mmol), *N^1^*-(7-chloroquinolin-4-yl)propane-1,3-diamine (0.117 g, 0.5 mmol), anhydrous sodium sulfate (Na_2_SO_4_) (0.071 g, 0.5 mmol), ytterbium (III) triflate (Yb(OTf)_3_) (0.024 g, 0.04 mmol), 2-isocyano-1-morpholino-3-phenylpropan-1-one (0.146 g, 0.6 mmol), and maleic anhydride (0.059 g, 0.6 mmol). This reaction resulted in the formation of compound **19f**, which was obtained as a foamy white solid weighing 0.195 g (63% yield). R*_f_* = 0.44 (AcOEt-MeOH = 90/5, *v*/*v*); ^1^H-NMR (500 MHz, CDCl_3_): 8.44 (d, 1H, *J* = 5.4 Hz, H-35), 7.99 (d, 1H, *J* = 9.0 Hz, H-41), 7.92 (s, 1H, H-15), 7.90 (d, 1H, *J* = 2.2 Hz, H-38), 7.38–7.31 (m, 2H, H-40, H-26), 7.17–7.05 (m, 7H, H-18, H-19, H-20, H-21, H-22, H-25, H-28), 6.90–6.77 (m, 1H, H-27), 6.67 (t, 1H, *J =* 6.4 Hz, H-32), 6.31 (d, 1H, *J* = 5.5 Hz, H-34), 5.82 (s, 1H, H-11), 4.30 (d, 1H, *J* = 14.0 Hz, H-16), 4.26 (d, 1H, *J* = 14.0 Hz, H-16′), 3.89–3.80 (m, 5H, H-29, H-2, H-6), 3.45–3.39 (m, 1H, H-31), 3.31–3.23 (m, 2H, H-29′, H-31′), 2.87–2.83 (m, 4H, H-3, H-5), 1.87–1.77 (m, 1H, H-30), 1.72–1.63 (m, 1H, H-30′); ^13^C-NMR (125 MHz, CDCl_3_): 168.2 (C-13), 162.4 (C-8), 162.3 (d, ^1^*J* = 251.8 Hz, C-24), 159.4 (C-10), 151.7 (C-35), 149.6 (C-33), 149.2 (C-37), 148.0 (C-7), 139.0 (C-17), 134.7 (C-39), 130.8 (d, ^3^*J* = 9.4 Hz, C-26), 129.4 (C-27), 128.5 (C-18, C-22), 128.3 (C-38), 128.1 (C-19, C-21), 126.1 (C-20), 125.1 (C-40), 124.7 (C-28), 124.1 (C-14), 123.5 (C-15), 122.3 (d, ^2^*J* = 12.0 Hz, C-23), 121.9 (C-41) 117.5 (C-42), 116.1 (d, ^2^*J* = 23.9 Hz, C-25), 98.3 (C-34), 66.9 (C-2, C-6), 59.6 (C-11), 52.9 (C-3, C-5), 39.9 (C-16), 38.9 (C-31), 37.8 (C-29), 25.9 (C-30); HRMS: (ESI^+^) *m/z* calcd. for [M-H]^+^ C_36_H_34_ ClFN_5_O_2_^+^ 622.2380, found 622.2373 (error = 1.1 ppm); IR (*ν*, cm^−1^): 1678 (C=O), 1575 (C=C), 1439 (C-C), 1113, 752 (C-F).


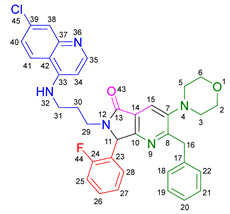


#### 3.2.8. 2-Benzyl-6-(2-((7-chloroquinolin-4-yl)amino)ethyl)-3-morpholino-7-(4-(trifluoromethyl)phenyl)-6,7-dihydro-5*H*-pyrrolo[3,4-*b*]pyridin-5-one **19g**

According to the general procedure, the following compounds were added sequentially to a PhCl (2.0 mL): 4-(trifluoromethyl)benzaldehyde (0.087 g, 0.5 mmol), *N*-(7-chloroquinolin-4-yl)ethane-1,2-diamine (0.111 g, 0.5 mmol), anhydrous sodium sulfate (Na_2_SO_4_) (0.071 g, 0.5 mmol), ytterbium (III) triflate (Yb(OTf)_3_) (0.024 g, 0.04 mmol), 2-isocyano-1-morpholino-3-phenylpropan-1-one (0.146 g, 0.6 mmol), and maleic anhydride (0.059 g, 0.6 mmol). This reaction resulted in the formation of compound **19g**, which was obtained as a foamy white solid weighing 0.252 g (77% yield). R*_f_* = 0.45 (AcOEt-MeOH = 90/5, *v*/*v*); ^1^H-NMR (500 MHz, CDCl_3_): 8.46 (d, 1H, *J* = 5.3 Hz, H-34), 7.91 (d, 1H, *J* = 2.2 Hz, H-37), 7.90 (s, 1H, H-15), 7.84 (d, 1H, *J* = 8.8 Hz, H-40), 7.61 (d, 2H, *J* = 8.3 Hz, H-25, H-27), 7.40 (dd, 1H, *J =* 8.8, 2.2 Hz, H-39), 7.32 (d, 2H, *J* = 8.5 Hz, H-24, H-28), 7.13–7.07 (m, 5H, H-18, H-19, H-20, H-21, H-22), 6.69 (s, 1H, H-31), 6.17 (d, 1H, *J* = 5.4 Hz, H-33), 5.59 (s, 1H, H-11), 4.26 (d, 1H, *J* = 14.0 Hz, H-16), 4.21–4.14 (m, 2H, H-16′, H-29), 3.80 (t, 4H, *J* = 4.6 Hz, H-2, H-6), 3.72–3.65 (m, 1H, H-29′), 3.45–3.39 (m, 1H, H-30), 3.30–3.24 (m, 1H, H-30′), 2.87–2.80 (m, 4H, H-3, H-5); ^13^C-NMR (125 MHz, CDCl_3_): 169.7 (C-13), 162.9 (C-8), 159.4 (C-10), 151.8 (C-34), 149.8 (C-32), 148.9 (C-36), 148.3 (C-7), 139.3 (C-23), 138.8 (C-17), 134.9 (C-38), 131.2 (q, ^2^*J* = 32.7 Hz, C-26), 128.6 (C-18, C-22), 128.4 (C-37), 128.2 (C-19, C-21), 128.1 (C-24, C-28), 126.2 (C-30), 126.1 (d, ^3^*J* = 3.8 Hz, C-25, C-27), 125.5 (C-39), 124.8 (q, ^1^*J* = 270 Hz, C-43), 123.6 (C-15), 123.0 (C-14), 121.9 (C-40), 117.1 (C-41), 98.2 (C-33), 66.9 (C-2, C-6), 66.1 (C-11), 52.9 (C-3, C-5), 43.8 (C-30), 39.9 (C-16), 40.6 (C-29); HRMS: (ESI^+^) *m/z* calcd. for [M-H]^+^ C_36_H_32_ ClF_3_N_5_O_2_^+^ 658.2191, found 658.2173 (error = 2.8 ppm); IR (*ν*, cm^−1^): 1696 (C=O), 1576 (C=C), 1331 (C=N), 1066 (C-F), 848 (C-C).


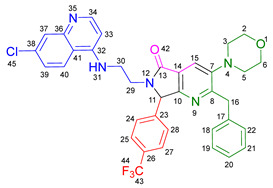


#### 3.2.9. 2-Benzyl-6-(2-((7-chloroquinolin-4-yl)amino)ethyl)-3-(piperidin-1-yl)-7-(4-(trifluoromethyl)phenyl)-6,7-dihydro-5*H*-pyrrolo[3,4-*b*]pyridin-5-one **19h**

According to the general procedure, the following compounds were added sequentially to a PhCl (2.0 mL): 4-(trifluoromethyl)benzaldehyde (0.087 g, 0.5 mmol), *N*-(7-chloroquinolin-4-yl)ethane-1,2-diamine (0.111 g, 0.5 mmol), anhydrous sodium sulfate (Na_2_SO_4_) (0.071 g, 0.5 mmol), ytterbium (III) triflate (Yb(OTf)_3_) (0.024 g, 0.04 mmol), 2-isocyano-3-phenyl-1-(piperidin-1-yl)propan-1-one (0.145, 0.6 mmol), and maleic anhydride (0.059 g, 0.6 mmol). This reaction resulted in the formation of compound **19h**, which was obtained as a foamy white solid weighing 0.192 g (59% yield). R*_f_* = 0.48 (AcOEt-MeOH = 90/5, *v*/*v*); ^1^H-NMR (500 MHz, CDCl_3_): 8.45 (d, 1H, *J* = 5.3 Hz, H-15), 7.92 (d, 1H, *J* = 2.2 Hz, H-18), 7.88–7.81 (m, 2H, H-7, H-21), 7.61 (d, 2H, *J* = 7.8 Hz, H-25, H-27), 7.41 (dd, 1H, *J* = 8.9, 2.2 Hz, H-20), 7.31 (d, 2H, *J* = 8.9 Hz, H-24, H-28), 7.13–7.08 (m, 5H, H-31, H-32, H-33, H-34, H-35), 6.72–6.66 (m, 1H, H-12), 6.16 (d, 1H, *J* = 5.4 Hz, H-14), 5.55 (s, 1H, H-2), 4.23 (d, 1H, *J* = 14.0 Hz, H-29), 4.17–4.12 (m, 2H, H-29′, H-10), 3.70–3.65 (m, 1H, H-10′), 3.43–3.38 (m, 1H, H-11), 3.28–3.22 (m, 1H, H-11′), 2.83–2.78 (m, 4H, H-37, H-41), 1.74–1.68 (m, 4H, H-38, H-40), 1.61–1.56 (m, 2H, H-39); ^13^C-NMR (125 MHz, CDCl_3_): 170.2 (C-9), 163.0 (C-5), 158.5 (C-3), 151.8 (C-15), 149.9 (C-13, C-17), 149.0 (C-6), 139.5 (C-23), 139.1 (C-30), 134.9 (C-19), 131.1 (d, ^2^*J* = 33.2 Hz, C-26), 128.8 (C-31, C-35), 128.4 (C-18), 128.2 (C-32, C-34), 128.0 (C-24, C-28), 126.1 (C-33), 126.1 (d, ^3^*J* = 3.8 Hz, C-25, C-27), 125.6 (C-20), 124.9 (q, ^1^*J* = 270 Hz, C-44), 123.0 (C-7), 122.8 (C-8), 121.9 (C-21), 117.2 (C-22), 98.2 (C-14), 66.1 (C-2), 54.2 (C-37, C-42), 43.9 (C-11), 40.7 (C-10), 39.7 (C-29), 26.3 (C-38, C-40), 23.8 (C-39); HRMS: (ESI^+^) *m/z* calcd. for [M-H]^+^ C_36_H_34_ ClF_3_N_5_O^+^ 656.2398, found 656.2372 (error = 4.0 ppm); IR (*ν*, cm^−1^): 1698 (C=O), 1557 (C=C), 1446 (C-C), 1381 (C=N), 1325, 1213, 1159 (C-N), 1128 (C=C), 1067, 1015 (C-F), 852, 691, 691 (C-C).


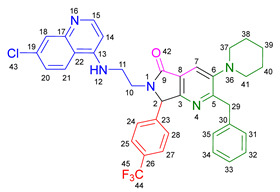


#### 3.2.10. 2-Benzyl-6-(2-((7-chloroquinolin-4-yl)amino)ethyl)-3-(diethylamino)-7-(4-(trifluoromethyl)phenyl)-6,7-dihydro-5*H*-pyrrolo[3,4-*b*]pyridin-5-one **19i**

According to the general procedure, the following compounds were added sequentially to a PhCl (2.0 mL): 4-(trifluoromethyl) benzaldehyde (0.087 g, 0.5 mmol), *N*-(7-chloroquinolin-4-yl)ethane-1,2-diamine (0.111 g, 0.5 mmol), anhydrous sodium sulfate (Na_2_SO_4_) (0.071 g, 0.5 mmol), ytterbium (III) triflate (Yb(OTf)_3_) (0.024 g, 0.04 mmol), *N*,*N*-diethyl-2-isocyano-3-phenylpropanamide (0.138 g, 0.6 mmol), and maleic anhydride (0.059 g, 0.6 mmol). This reaction resulted in the formation of compound **19i**, which was obtained as a foamy white solid weighing 0.202 g (63% yield). R*_f_* = 0.48 (AcOEt-MeOH = 90/5, *v*/*v*); ^1^H-NMR (500 MHz, CDCl_3_): 8.46 (d, 1H, *J* = 5.3 Hz, H-15), 7.92 (d, 1H, *J* = 2.0 Hz, H-18), 7.87–7.84 (m, 2H, H-7, H-21), 7.61 (d, 2H, *J* = 2.0 Hz, H-25, H-27), 7.40 (dd, 1H, *J* = 8.9, 2.2 Hz, H-20), 7.32 (d, 2H, *J* = 8.0 Hz, H-24, H-28), 7.12–7.05 (m, 5H, H-31, H-32, H-33, H-34, H-35), 6.75–6.68 (m, 1H, H-12), 6.17 (d, 1H, *J* = 5.4 Hz, H-14), 5.56 (s, 1H, H-2), 4.25 (d, 1H, *J* = 14.0 Hz, H-29), 4.19–4.12 (m, 2H, H-29′, H-10), 3.72–3.65 (m, 1H, H-10′), 3.75–3.37 (m, 1H, H-11), 3.30–3.22 (m, 1H, H-11′), 2.98 (q, 4H, *J* = 7.1 Hz, H-37, H-42), 0.92 (t, 6H, *J* = 7.1 Hz, H-38, H-43); ^13^C-NMR (125 MHz, CDCl_3_): 170.1 (C-9), 164.4 (C-5), 158.7 (C-3), 151.9 (C-15), 149.8 (C-13), 149.0 (C-17), 147.0 (C-6), 139.5 (C-23), 139.1 (C-30), 134.9 (C-19), 131.17 (d, ^2^*J* = 32.6 Hz, C-26), 128.8 (C-31, C-35), 128.5 (C-18), 128.2 (C-24, C-28), 127.9 (C-32, C-34), 126.1 (d, ^3^*J* = 3.8 Hz, C-25, C-27), 126.0 (C-33), 125.5 (C-20), 125.4 (C-7), 123.7 (d, ^1^*J* = 272.4 Hz, C-41), 122.4 (C-8), 121.9 (C-21), 117.2 (C-22), 98.2 (C-14), 66.2 (C-2), 47.6 (C-37, C-42), 43.9 (C-11), 40.7 (C-10), 39.8 (C-29), 12.0 (C-38, C-43); HRMS: (ESI^+^) *m/z* calcd. for [M-H]^+^ C_36_H_34_ ClF_3_N_5_O^+^ 644.2398, found 644.2403 (error = 0.6 ppm); IR (*ν*, cm^−1^): 1702 (C=O), 1580 (C=C), 1447 (C-C), 1385 (C=N), 1323 (C-N), 1153, 1072 (C-F), 698 (C-C).


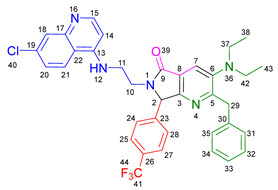


#### 3.2.11. 2-Benzyl-7-(3,5-bis(trifluoromethyl)phenyl)-6-(2-((7-chloroquinolin-4-yl)amino)ethyl)-3-morpholino-6,7-dihydro-5*H*-pyrrolo[3,4-*b*]pyridin-5-one **19j**

According to the general procedure, the following compounds were added sequentially to a PhCl (2.0 mL): 3, 5-bis(trifluoromethyl)benzaldehyde (0.121 g, 0.5 mmol), *N*-(7-chloroquinolin-4-yl)ethane-1,2-diamine (0.111 g, 0.5 mmol), anhydrous sodium sulfate (Na_2_SO_4_) (0.071 g, 0.5 mmol), ytterbium (III) triflate (Yb(OTf)_3_) (0.024 g, 0.04 mmol), 2-isocyano-1-morpholino-3-phenylpropan-1-one (0.146 g, 0.6 mmol), and maleic anhydride (0.059 g, 0.6 mmol). This reaction resulted in the formation of compound **19j**, which was obtained as a foamy white solid weighing 0.264 g (73% yield). R*_f_* = 0.47 (AcOEt-MeOH = 90/5, *v*/*v*); ^1^H-NMR (500 MHz, CDCl_3_): 8.48 (d, 1H, *J* = 5.3 Hz, H-34), 7.93–7.89 (m, 3H, H-37, H-15, H-26), 7.82 (d, 1H, *J* = 8.9 Hz, H-40), 7.69–7.66 (m, 2H, H-24, H-28), 7.42 (dd, 1H, *J* = 8.9, 2.2 Hz, H-39), 7.16–7.06 (m, 5H, H-18, H-19, H-20, H-21, H-22), 6.56 (s, 1H, H-31), 6.22 (d, 1H, *J* = 5.4 Hz, H-33), 5.65 (s, 1H, H-11), 4.33–4.25 (m, 1H, H-29), 4.24 (d, 1H, *J* = 14.0 Hz, H-16), 4.20 (d, 1H, *J* =14.0 Hz, H-16′), 3.85–3.81 (m, 4H, H-2, H-6), 3.66–3.59 (m, 1H, H-29′), 3.51–3.43 (m, 1H, H-30), 3.39–3.31 (m, 1H, 30′), 2.92–2.85 (m, 4H, H-3, H-5); ^13^C-NMR (125 MHz, CDCl_3_): 169.9 (C-13), 163.3 (C-8), 158.4 (C-10), 151.9 (C-34), 149.7 (C-32), 148.9 (C-36), 148.6 (C-7), 138.6 (C-17), 138.3 (C-23), 135.0 (C-38), 132.7 (q, ^2^*J* = 33.7 Hz, C-25, C-27), 128.7 (C-18, C-22), 128.5 (C-37), 128.2 (C-19, C-21), 127.8 (C-24, C-28), 126.3 (C-20), 125.7 (C-39), 123.8 (C-15), 123.1 (C-26), 122.8 (C-14), 121.7 (C-40), 119.6 (C-44, C-45), 117.1 (C-41), 98.3 (C-33), 67.0 (C-2, C-6), 65.4 (C-11), 59.2 (C-3, C-5), 43.8 (C-30), 40.7 (C-29), 39.9 (C-16); ^19^F-NMR (500 MHz, CDCl_3_): −64.5 (F-43, F-46); HRMS: (ESI^+^) *m/z* calcd. for [M-H]^+^ C_37_H_31_ ClF_6_N_5_O_2_^+^ 726.2065, found 726.2035 (error = 4.1 ppm); IR (*ν*, cm^−1^): 1693 (C=O), 1583 (C=C), 1375 (C=N), 1272, 1134 (C=C), 898 (C-C).


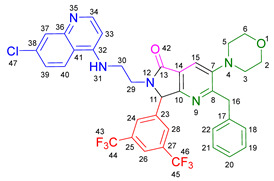


#### 3.2.12. 2-Benzyl-7-(3,5-bis(trifluoromethyl)phenyl)-6-(2-((7-chloroquinolin-4-yl)amino)ethyl)-3-(piperidin-1-yl)-6,7-dihydro-5*H*-pyrrolo[3,4-*b*]pyridin-5-one **19k**

According to the general procedure, the following compounds were added sequentially to a PhCl (2.0 mL): 3, 5-bis(trifluoromethyl) benzaldehyde (0.121 g, 0.5 mmol, *N*-(7-chloroquinolin-4-yl)ethane-1,2-diamine (0.111 g, 0.5 mmol), anhydrous sodium sulfate (Na_2_SO_4_) (0.071 g, 0.5 mmol), ytterbium (III) triflate (Yb(OTf)_3_) (0.024 g, 0.04 mmol), 2-isocyano-3-phenyl-1-(piperidin-1-yl)propan-1-one (0.145, 0.6 mmol), and maleic anhydride (0.059 g, 0.6 mmol). This reaction resulted in the formation of compound **19k**, which was obtained as a foamy white solid weighing 0.263 g (73% yield). R*_f_* = 0.52 (AcOEt-MeOH = 90/5, *v*/*v*); ^1^H-NMR (500 MHz, CDCl_3_): 8.48 (d, 1H, *J* = 5.3 Hz, H-15), 7.93 (d, 1H, *J* = 2.1 Hz, H-18), 7.90–7.88 (m, 1H, H-26), 7.85 (s, 1H, H-7), 7.83 (d, 1H, *J* = 9.0 Hz, H-21), 7.68–7.65 (m, 2H, H-24, H-28), 7.43 (dd, 1H, *J* = 8.9, 2.2 Hz, H-20), 7.13–7.09 (m, 5H, H-31, H-32, H-33, H-34, H-35), 6.57 (t, 1H, *J* = 4.1 Hz, H-12), 6.21 (d, 1H, *J* = 5.3 Hz, H-14), 5.61 (s, 1H, H-2), 4.32–4.25 (m, 1H, H-10), 4.21–4.15 (m, 2H, H-29, H-29′), 3.65–3.59 (m, 1H, H-10′), 3.50–3.42 (m, 1H, H-11), 3.37–3.31 (m, 1H, H-11′), 2.86–2.81 (m, 4H, H-37, H-41), 1.76–1.71 (m, 4H, H-38, H-40), 1.63–1.57 (m, 2H, H-39); ^13^C-NMR (125 MHz, CDCl_3_): 170.3 (C-9), 163.3 (C-5), 157.5 (C-3), 152.0 (C-15), 150.2 (C-13), 149.7 (C-17), 149.1 (C-6), 138.9 (C-23), 138.5 (C-30), 134.9 (C-19), 132.6 (q, ^2^*J* = 32.5 Hz, C-25, C-27), 128.8 (C-31, C-35), 128.6 (C-18), 128.1 (C-32, C-34), 127.8 (C-24, C-28), 126.2 (C-33), 125.7 (C-20), 123.1 (C-7), 123.0 (C-26), 122.5 (C-8), 121.8 (C-21), 119.7 (C-44, C-46), 117.2 (C-22), 98.3 (C-14), 65.4 (C-2), 54.2 (C-37, C-41), 43.9 (C-11), 40.7 (C-10), 39.7 (C-29), 26.3 (C-38, C-40), 23.9 (C-39); ^19^F-NMR (500 MHz, CDCl_3_): −64.5 (F-45, F-47); HRMS: (ESI^+^) *m/z* calcd. for [M-H]^+^ C_38_H_33_ ClF_6_N_5_O^+^ 724.2272, found 724.2297 (error = 3.4 ppm); IR (*ν*, cm^−1^): 1686 (C=O), 1575 (C=C), 1375 (C=N), 1279 (C-F), 1134 (C-C).


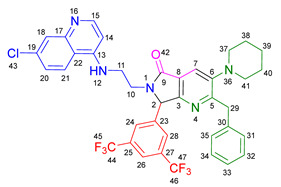


#### 3.2.13. 2-Benzyl-6-(3-((7-chloroquinolin-4-yl)amino)propyl)-3-morpholino-7-(perfluorophenyl)-6,7-dihydro-5*H*-pyrrolo[3,4-*b*]pyridin-5-one **19l**

According to the general procedure, the following compounds were added sequentially to a PhCl (2.0 mL): 2, 3, 4, 5, 6-pentafluorobenzaldehyde (0.098 g, 0.5 mmol), *N^1^*-(7-chloroquinolin-4-yl)propane-1,3-diamine (0.117 g, 0.5 mmol), anhydrous sodium sulfate (Na_2_SO_4_) (0.071 g, 0.5 mmol), ytterbium (III) triflate (Yb(OTf)_3_) (0.024 g, 0.04 mmol), 2-isocyano-1-morpholino-3-phenylpropan-1-one (0.146 g, 0.6 mmol), and maleic anhydride (0.059 g, 0.6 mmol). This reaction resulted in the formation of compound **19l**, which was obtained as a foamy white solid weighing 0.173 g (50% yield). R*_f_* = 0.49 (AcOEt-MeOH = 90/5, *v*/*v*); ^1^H-NMR (500 MHz, CDCl_3_): 8.48 (d, 1H, *J* = 5.4 Hz, H-35), 7.97 (d, 1H, *J* = 9.2 Hz, H-41), 7.93–7.90 (m, 2H, H-15, H-38), 7.40 (dd, 1H, *J* = 2.2, 8.9 Hz, H-40), 7.22–7.12 (m, 5H, H-18,H-19, H-20, H-21, H-22), 6.51 (t, 1H, *J* = 6.4 Hz, H-32), 6.32 (d, 1H, *J* = 5.4 Hz, H-34), 5.87 (s, 1H, H-11), 4.32 (d, 1H, *J* = 14.2 Hz, H-16), 4.26 (d, 1H, *J* = 14.2 Hz, H-16′), 3.97–3.00 (m, 1H, H-29), 3.85–3.81 (m, 4H, H-2, H-6), 3.53–3.46 (m, 1H, H-31), 3.36–3.25 (m, 2H, H-29′, H-31′), 2.90–2.86 (m, 4H, H-3, H-5), 1.94–1.87 (m, 1H, H-30), 1.80–1.73 (m, 1H, H-30′); ^13^C-NMR (125 MHz, CDCl_3_): 168.1 (C-13), 162.7 (C-8), 157.2 (C-10), 151.8 (C-35), 149.5 (C-33), 149.3 (C-37), 148.7 (C-7), 147.6 (C-25, C-27), 146.4 (C-26), 144.3 (C-24, C-28), 139.0 (C-17), 134.9 (C-39), 128.6 (C-18, C-22), 128.3 (C-19, C-21, C-38), 126.3 (C-20), 125.4 (C-40), 124.0 (C-14), 123.8 (C-15), 121.7 (C-41), 117.5 (C-42), 98.3 (C-34), 67.0 (C-2, C-6), 55.3 (C-11), 53.0 (C-3, C-5), 39.9 (C-16), 38.7 (C-31), 38.0 (C-29), 26.1 (C-30); ^19^F-NMR (500 MHz, CDCl_3_): −142.5 (d, *J* = 21.96 Hz, F-43, F-47), −145.1 (d, *J* = 22.63 Hz, F-43, F-47), −152.4 (t, *J* = 20.68 Hz, F-45), −161.5 (td, *J* = 21.61, 8.36 Hz, F-44, F-46), −161.8 (td, *J* = 21.61, 8.11 Hz, F-44, F-46); HRMS: (ESI^+^) *m/z* calcd. for [M-H]^+^ C_36_H_30_ ClF_5_N_5_O_2_^+^ 694.2003, found 694.1976 (error = 3.8 ppm); IR (*ν*, cm^−1^): 1688 (C=O), 1584 (C=C), 1500, 1107 (C-F), 995 (C-C).


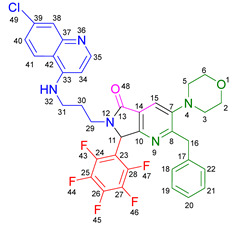


### 3.3. In Vitro Studies

#### 3.3.1. Cell Line and Virus

Cells and virus were obtained and maintained as previously described [34]. Vero E6 cells were cultured at 37 °C with 5% CO_2_ in low-glucose Dulbecco’s Modified Eagle Medium supplemented with 8% fetal bovine serum. The SARS-CoV-2 strain was isolated from a PCR-positive patient. All experiments involving SARS-CoV-2 were conducted in a Biosafety Level 3 laboratory at the National Autonomous University of Mexico. Virus stocks and titers were produced in Vero E6 cells, and aliquots were stored at −80 °C until use.

#### 3.3.2. Cell Viability Assay

The effect of the compounds on cell viability was assessed as previously described [38]. The following process is briefly outlined, Vero cells (25,000/well) were seeded in a 96-well plate with DMEM (8% FBS, glutamine) and incubated at 37 °C, 5% CO_2_ for 24 h before compound addition at various concentrations in triplicate (n = 9). After 48 h, the medium was removed, and cells were fixed, stained (crystal violet/formaldehyde), washed, and treated with acetic acid to release the dye. Absorbance was measured at 590 nm (Sunrise, TECAN, Männedorf, Switzerland), and cell viability was normalized to the compound dilution vehicle.

#### 3.3.3. Time Addition Assay

All tested compounds were initially dissolved in dimethyl sulfoxide (DMSO) to prepare 10 mM stock solutions. DMSO was selected for its capacity to dissolve poorly water-soluble and non-polar compounds while persevering cell viability. A concentration of 10 μM of each compound was ensured by accurate weighing of the solids and subsequently diluting them to 10 mM under standardized conditions. All solutions were freshly prepared prior to each assay. Solubilization and treatment reproducibility were confirmed through biological triplicates with technical duplicates. No crystal formation was observed during incubation conditions (37 °C) [41,42]. Before use in cell-based assays, stock solutions were diluted in DMEM to achieve the appropriate working concentrations. The final DMSO concentration in all experimental conditions did not exceed 0.1% (*v*/*v*), to avoid solvent-associated cytotoxic effects. Compounds (10 μM) were added to the cells either during infection (0 h) or 1 h postinfection and viral titers were then quantified by viral plaques assay 24 postinfection. All infections were carried out using a virus concentration of a MOI of 0.1. The methodology has been previously described [43].

The half-maximal inhibitory concentration (IC_50_) values were estimated based on the antiviral activity observed across different compound concentrations. Linear regression analysis was applied to the data points falling within the linear range of the dose-response curve. All calculations were performed using the following formula:

$$Y=Y_{0}+\frac{Y_{1}-Y_{0}}{X_{1}-X_{o}}\left(X-X_{o}\right),$$

The results were obtained from experiments conducted in biological triplicates with technical duplicates to ensure reproducibility.

### 3.4. In Silico Studies (Docking and Molecular Dynamics)

Computer-aided drug design (CADD) encompasses computational approaches that support the discovery and optimization of bioactive molecules. In this study a structure-based drug design (SBDD) strategy was employed (Figure S80), incorporating in-silico ADME/Tox filters, multi-engine docking (AD4, Vina, Vinardo) with consensus/clustering-based selection explicit-solvent MD refinement, and MM/GBSA binging-energy estimation. Detailed procedures are provided in the following sections.

#### 3.4.1. ADME and Tox Properties

The ADMETox assessment of compounds **19d** and **19i** was performed using five complimentary servers. Initially, SwissADME [44] (http://www.swissadme.ch/ (accessed on 7 October 2024)) assessed physicochemical characteristics, solubility, and drug-likeness criteria (e.g., Lipinski and Veber). Subsequently, vNNadmet [45] (https://vnnadmet.bhsai.org/vnnadmet/ (accessed on 7 October 2024)) used neural networks to predict parameters about the absorption of medicines in the gastrointestinal tract, their interactions with cytochrome P450 enzymes, and potential transporters. Concurrently, PredSkin [46] (http://predskin.labmol.com.br/ (accessed on 7 October 2024)) evaluated the capability to enhance skin sensitivity with machine learning-QSAR models. They accomplished this by doing several simulations (DPRA, KeratinoSens, h-CLAT, and LLNA) to determine a conclusive outcome (sensitizer or non-sensitizer). ProTox-III [47] (https://tox.charite.de/protox3/ (accessed on 7 October 2024)) integrates toxicological data from various sources with machine learning algorithms to ascertain acute toxicity (LD_50_), categorize each molecule by its toxicological class, and assess the risks of mutagenicity, immunotoxicity, neurotoxicity, and cardiotoxicity. Finally, pkCSM [48] (https://biosig.lab.uq.edu.au/pkcsm/ (accessed on 7 October 2024)) employs machine learning methodologies utilizing chemical distance metrics to predict the absorption (including Caco-2 permeability), distribution (including volume of distribution and plasma protein binding), metabolism (as a CYP substrate or inhibitor), elimination (clearance), and toxicity (AMES, hERG, hepatotoxicity) of chemicals. This cohort of servers examined many features, including the solubility of the medications and the probability of adverse events occurring [40,49]. This information was essential in planning and optimizing future research including **19d** and **19i** (Figure S80)**.**

#### 3.4.2. Target Identification and Active Pocket Assessment

The choice of protein targets for ligands **19d** and **19i** was motivated by their established functions in the SARS-CoV-2 infection mechanism. The selected the non-estructured proteins (nsp) are as follows: Main Protease (M_pro_) (nsp5, PDB ID: 8D35 [50]), Replicase (nsp7, PDB ID: 7TZJ [51]), RNA Polymerase (nsp12, PDB ID: 7ED5 [52]), Helicase (nsp13, PDB ID: 7CXN [53]), and the Spike protein in its N-terminal (NTD) and receptor-binding (RBD) domains from the Alpha (α) B.1.1.7 and Omicron (ο) B.1.1.529 variants (PDB IDs: 7FET [54] and 8H3M [55] respectively) or NTDα, NTDο, RBDα, and RBDο. Active binding pockets in each target were identified using co-crystallized ligands and subsequently refined through the Graph-based Residue Neighborhood Strategy to Predict Binding Sites (GRaSP) [56,57] and PrankWeb3 [58], a machine learning approach utilizing random forests to identify potential ligand-binding sites on the protein’s solvent-accessible Surface [59] (Figure S80).

At the beginning of this project, the structure 8D35 (https://www.wwpdb.org/pdb?id=pdb_00008d35 (accessed on 7 July 2024)) was the only available model, and all computational analyses were conducted using this structure. After the manuscript was submitted for consideration in this journal, a reviewer pointed out that an updated structure, 9DW6, had since been released. Naturally, we compared both structures and found no appreciable differences that would affect the validity of our conclusions. A detailed comparison is provided in the Supporting Information (Figure S81). Therefore, the computational work presented in this article remains based on structure 8D35.

The accuracy of docking and binging free energy calculations can be limited by the resolution and quality of the input protein structure. Medium-to-low resolution models (>2.5 Å) often exhibit ambiguous side-chain orientations, absence of structural waters, and uncertain hydrogen-bonding networks, which may compromise docking accuracy [60,61]. To mitigate these limitations, we employed the following strategies: (i) binding site identifications using co-crystallized ligand, GRaSP and PrankWeb3; (ii) multi-engine docking with clustering to reduce scoring biases; (iii) MD replicates to assess binging stability; and (iv) a focus on comparative MM/GBSA trends rather than absolute energies values. This multi-tiered approach helps ensure the reliability of comparative binding insights despite inherent structural uncertainties.

#### 3.4.3. Homology Modeling and Docking Simulations

Swiss-Model [62] was used for homology modeling to address missing crystallographic regions. The resultant models were evaluated using GMQE and QMEAN scores, both of which were needed to surpass 0.9. Conclusive structural validations were conducted using MolProbity [63] and VERIFY3D [64]. Mutations in the NT and RB domains of the spike protein (Alpha and Omicron variants) were included based on data from https://covariants.org/ (accessed on 7 July 2024) using Chimera [65], with accurate rotamer selection for each substituted residue ensured [66].

#### 3.4.4. Preparation of the System for Docking

All protein structures were subjected to energy minimization in an aqueous environment (6 Å solvent shell) using the YASARA server [67]. Protonation states were established at pH 7.4, and minimization ceased when the energy variation fell below 0.01 kcal/mol (0.05 kJ/mol) during 200 steepest-descent iterations [68]

#### 3.4.5. Docking Protocol

Docking simulations were conducted using AutoDock Vina v1.2.5, establishing precise grid dimensions for each target. Three separate force fields—AD4, Vina, and Vinardo—were used. A total of 42,000 individual docking experiments were conducted, with each of the two ligands (**19d** and **19i**) docked 500 times in each target cavity throughout each force field. The multi-force-field methodology has shown strong dependability [69,70]. Subsequent to the first docking, all ligand conformations were subjected to a redocking process and then clustered using a 0.5 Å threshold. The final ligand conformations chosen for molecular dynamics (MDs) simulations were those with binding scores at least one standard deviation below the mean and inside the most populated cluster [71,72] (Figure S80).

#### 3.4.6. Molecular Dynamics Simulations: System Construction

MD simulations were performed via GROMACS [73]. The protonation states of each protein were confirmed at pH 7.4 using PropKa on the PDB2PQR service [74] (https://server.poissonboltzmann.org/ (accessed on 2 September 2024)). Input files were produced using CHARMM-GUI [75], with the CHARMM36m force field [76]. Ligand parameters for **19d** and **19i** were acquired using the Ligand Reader and Modeler tool in CHARMM-GUI [77]. Each protein–ligand combination was placed in an orthorhombic box, maintaining a minimum distance of 15 Å from the box edges, and was solubilized with TIP3P water. 0.15 M NaCl was introduced to neutralize the net charge. This approach produced 30 systems (20 with nsp proteins and 10 with NT or RB Spike domains), each duplicated five times.

A preliminary energy reduction consisting of 10,000 steepest-descent steps was conducted, followed by two equilibration phases under distinct ensembles: (i) NVT for 10 ns and (ii) NPT for 20 ns. A 150 ns production run was then performed in the NPT ensemble with a 4 fs timestep, facilitated by Hydrogen Mass Repartitioning (HMR) [78]. Periodic Boundary Conditions (PBCs) and Particle Mesh Ewald (PME) were used to address long-range electrostatic interactions. In the NPT phase, the temperature was sustained at 310.15 K using a velocity-rescale thermostat, while the pressure was regulated at 1 bar using the Parrinello–Rahman barostat. Five separate molecular dynamics replicates were conducted for each ligand-target combination, resulting in a total simulation duration of 7.0 μs across all systems. MDAnalysis version 2.6.1 [79] was used for trajectory analysis, while all molecular visualizations were produced using Chimera [65].

#### 3.4.7. Calculations of Binding Free Energy: MM/GBSA Methodology

Binding free energies were calculated via the MM/GBSA methodology in gmx_MMPBSA [80,81,82], a program adapted from AMBER’s MMPBSA.py [83,84]. The GB approximation was used to effectively include solvation effects.

Representative structures were obtained by concatenating the equilibrated segments of the five replicas for each system, then followed by a clustering analysis [85,86]. Clustering was performed using GROMACS’s cluster tool with a 2.5 Å cutoff, concentrating on Cα atoms, and ensuring that a minimum of 75% of all frames [87,88] were included into the primary cluster.

The binding free energy $\left(∆G_{binding}\right)$ was calculated using:

$$∆G_{binding}=\;∆G_{complex}-\left(∆G_{protein}+\;∆G_{ligand}\right)$$

where $∆G_{complex},\;∆G_{protein},\;\mathrm{and}\;∆G_{ligand}$ represent the free energies of the complex, the protein, and the ligand, respectively, under identical simulation conditions. Each free energy term 〈G〉 is defined by the Equation:

$$G=\;E_{MM}+\;∆G_{solv}-\;TS_{MM}\;$$

where $E_{MM}$ is the molecular mechanics energy, $∆G_{solv}$ is the solvation energy, T is the temperature, and $S_{MM}$ is the molecular mechanics entropy.

The observed binding free energies account just for the enthalpic contribution, while the entropic component $\left(TS_{MM}\right)$ was excluded [69,82]. The interactions between protein and ligand were then analyzed using PLIP v2.2.2 [89,90].

The flux of computational methodology can be in the Supplementary Materials.

### 3.5. In Silico Studies (Molecular Structure)

#### Non-Covalent Interactions by Using the Electron Density

The quantum theory of atoms in molecules (QTAIM) [91,92,93] and the non-covalent interactions index (NCI) [94] have been important quantum chemistry tools to analyze non-covalent interactions. In particular, these two approaches are useful to understand, under an atomistic point of view, the interaction drug–receptor [95,96].

The QTAIM is based on the analysis of the electron density, $\rho \left(r\right)$, and its derivatives, like Laplacian and Hessian. Localization of $\rho \left(r\right)$ critical points, especially bond critical points (bcp), is crucial to predict contacts between atoms, in our case, non-covalent interactions are the main target. The sign of the Laplacian of the electron density, the ratio between local potential energy ($V\left(bcp\right)$) and local kinetic energy ($G\left(bcp\right)$), and the local total energy of bond ($H\left(bcp\right)$), evaluated at the bcp, are important elements to characterize non-covalent interactions. Additional to these quantities, the NCI predicts zones where non-covalent interactions are present; the NCI helps to characterize weak and non-directional interactions. Thus, coupling QTAIM and NCI is a good alternative to elucidate the nature of non-covalent interactions. In addition, the molecular electrostatic potential (MEP) [97] has always been an important tool to predict possible local electrostatic contacts between different fragments. In summary, QTAIM, NCI, and MEP are quantum chemistry approaches to analyze non-covalent interactions in this article.

As it was mentioned above, for our analysis, electron density is required. Because the size of the systems considered in this article is large, our systems are required to be modeled. The Spike NTDα/**19d**, NTDo/**19d**, and nsp5/**19i** were modeled using all residues containing at least one atom within a maximum distance of 5.0 Å from the ligand; residues beyond this cutoff were discarded. The peptide fragments that were made had caps added to the N- and C-termini by methylation to keep the peptide bonds from breaking. This methodology has been applied in other studies [98,99,100].

The COSMO solvation model (ε = 4.0) was used to simulate the internal medium of a protein [101]; a single-point energy calculation was performed at the PBE0-D3/6-311G** level of theory [102,103,104,105,106,107] using the TeraChem suite code [108,109,110]. Analysis of the electron density through the QTAIM, NCI, and MEP were obtained by using the graphics processing units for atoms and molecules (GPUAM) code [111,112,113].

## 4. Conclusions

This new MCR-based method shows significant improvements in quickly and effectively synthesizing complex polyheterocycles that have antiviral properties. By using the new method that combines an UZ-3CR and a μw-assisted *aza*-Diels–Alder/*N*-acylation/decarboxylation/dehydration process in one step in chlorobenzene—this is the first time this solvent has been used in such reactions—twelve fluorinated 4-amino-7-chloroquinoline-pyrrolo[3,4-*b*]pyridin-5-ones were prepared, achieving yields between 50 and 77% with atom economy values from 88 to 90%. In Vero E6 cells, early SARS-CoV-2 replication was significantly inhibited by compounds **19d** (IC_50_ = 6.74 µM) and **19i** (IC_50_ = 5.29 µM) when delivered at 0h and 1h postinfection, respectively. At 10 µM, cell viability remained above 90% relative to control vehicle. Simultaneous computer analyses showed strong interactions with key viral targets, supporting a two-part action that blocks both the virus from entering cells and the processing needed for its genetic material to mature. Collectively, our results establish **19d** and **19i** as potential candidates for the advancement of broad-spectrum antivirals targeting SARS-CoV-2 and its variations. Molecular mechanics simulations for compound **19i** revealed stable and high-affinity interactions with M_pro_, in line with its experimental antiviral activity. Besides, compound **19d** compound exhibited strong affinity toward the *N*-terminal domain of the Spike protein, suggesting a blockage in viral entry. Future research should focus on improving the structure of the drugs to make them work better and have fewer side effects, testing their effectiveness in animal studies, and evaluating how well the drug formulations maintain effective levels in the body, which will help us be ready for current and future viral outbreaks.

## Figures and Tables

**Table 1:**
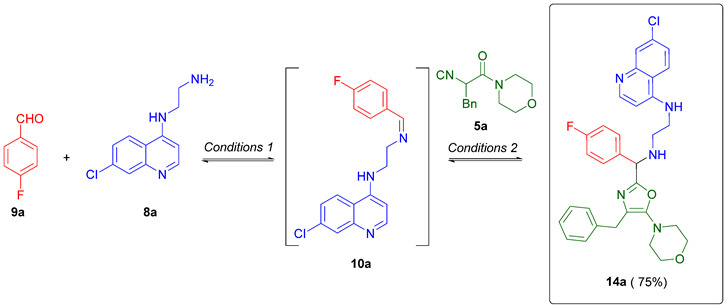
Optimization of UZ-3CR conditions.

Entry	Solvent	Conditions 1 Additives/T_1_ (°C)	Conditions 2 Additives/T_2_ (°C)	Yield (%) ^a^
1	PhMe	Na_2_SO_4 (anh.)_/r.t (25)	InCl_3_ (15% mol) MW (70)	-
2	PhMe	Na_2_SO_4 (anh.)_/MW (80)	InCl_3_ (15% mol) MW (70)	-
3	MeOH	Na_2_SO_4 (anh.)_/MW (80)	InCl_3_ (15% mol) MW (70)	8
4	PhMe/MeOH 9:2 *v*/*v*	Na_2_SO_4 (anh.)_/MW (80)	InCl_3_ (15% mol) MW (70)	14
5	PhMe/MeOH 9:2 *v*/*v*	Na_2_SO_4 (anh.)_/MW (80)	InCl_3_ (15% mol) MW (80)	19
6	PhCl	Na_2_SO_4 (anh.)_/MW (80)	InCl_3_ (15% mol) MW (80)	41
7	PhCl	Na_2_SO_4 (anh.)_/MW (80)	Sc(OTf)_3_ (5% mol) MW (80)	52
8	PhCl	Na_2_SO_4 (anh.)_/MW (80)	Yb(OTf)_3_ (5% mol) MW (80)	75

**^a^** Yields calculated after the purification of the compound **14a**. Color key: red (aldehyde moiety), blue (amine moiety) and green (isocyanide moiety).

**Table 2:**
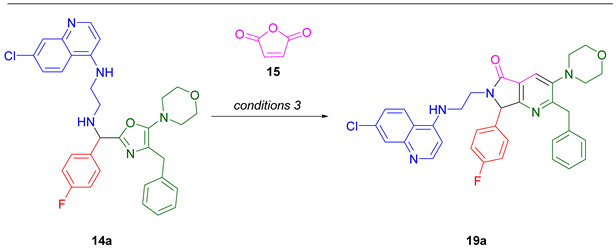
Optimization of conditions for cascade process between 5-aminooxazole and maleic anhydride.

Entry	Solvent	Conditions 3 T_1_ (°C)	Yield (%) ^a^
1	PhMe/MeOH 9:2	MW (70)	79
2	PhMe/MeOH 9:2	MW (80)	74
3	PhCl	MW (80)	94

^a^ Yields calculated after the purification of the compound **19a**. Color key: red (aldehyde moiety), blue (amine moiety), green (isocyanide moiety) and pink (anhydride moiety).

**Table 3:** IC_50_ of the compounds tested.

	19a	19b	19c	19d	19e	19f	19g	19h	19i	19j	19k	19l
IC_50_ (µM) 0 h	-	-	-	6.74 *	17.14	9.23	7.50	-	24.00	15.00	-	7.06
IC_50_ (µM) 1 h	-	-	-	-	-	-	6.98	-	5.29 *	-	-	-

- ND; * these compounds correspond to the lowest IC_50_ values, indicating high antiviral potency at the corresponding time point.

## Data Availability

The data presented in this study is available on request from the corresponding authors.

## Supplementary Materials

The supporting information can be downloaded at: https://www.mdpi.com/article/10.3390/ijms26157651/s1.
