# Supplementary material for: Multi-Component Synthesis of New Fluorinated-Pyrrolo[3,4-b]pyridin-5-ones Containing the 4-Amino-7-chloroquinoline Moiety and In Vitro–In Silico Studies Against Human SARS-CoV-2
Source: Int J Mol Sci. 2025 Aug 7;26(15):7651. doi: 10.3390/ijms26157651 (PMC12347832; doi:10.3390/ijms26157651)
Supplement: Supplementary file 1 [file ijms-26-07651-s001.zip › ijms-3728777-supplementary.pdf]

ELECTRONIC SUPPLEMENTARY MATERIAL

Multi-component Synthesis of New Fluorinated-Pyrrolo[3,4-*b*]pyridin-5-ones Containing the 4-amino-7-chloroquine Moiety and In Vitro–In Silico Studies Against Human SARS-CoV-2

Roberto E. Blanco-Carapia [1], Ricardo Hernández-López [1], Sofía L. Alcaraz-Estrada [2], Rosa Elena Sarmiento-Silva [3], Montserrat Elemi García-Hernández [3], Nancy Viridiana Estrada-Toledo [4], Gerardo Padilla-Bernal [1], Leonardo D. Herrera-Zúñiga [1,5], Jorge Garza [1], Rubicelia Vargas [1,*], Eduardo González-Zamora [1] and Alejandro Islas-Jácome [1,*]

1 Departamento de Química, Universidad Autónoma Metropolitana–Iztapalapa, Av. Ferrocarril San Rafael Atlixco 186, Col. Leyes de Reforma 1A Sección, Iztapalapa, Ciudad de México C.P. 09310, Mexico; edreyblanco@gmail.com (R.E.B.-C.); richi.quimera72@gmail.com (R.H.-L.); requiem900909@gmail.com (G.P.-B.); lherrera@cua.uam.mx (L.D.H.-Z.); jgarza@izt.uam.mx (J.G.); egz@xanum.uam.mx (E.G.-Z.)

2 División de Medicina Genómica, Centro Médico Nacional 20 de Noviembre, Instituto de Seguridad y Servicios Sociales de los Trabajadores del Estado (ISSSTE), Félix Cuevas 540, Col. Del Valle Sur, Benito Juárez, Ciudad de México C.P. 03100, Mexico; sofializeth@gmail.com (S.L.A.-E.)

3 Departamento de Microbiología e Inmunología, Facultad de Medicina, Veterinaria y Zootecnia, Universidad Nacional Autónoma de México, Av. Universidad 3000, Ciudad Universitaria, Coyoacán, Ciudad de México C.P. 04510, Mexico; rosass@unam.mx (R.E.S.-S.); elemi.gh@gmail.com (M.E.G.-H.)

4 Health Pharma Professional Research S.A de C.V., Av. Insurgentes Sur 662-Piso 3, Col. Del Valle, Benito Juárez, Ciudad de México C.P. 03100, Mexico; nefrtty-n_ancy@hotmail.com (N.V.E.-T.)

5 Departamento de Ciencias Naturales, Universidad Autónoma Metropolitana–Cuajimalpa, Vasco de Quiroga 4871, Col. Contadero, Cuajimalpa, Ciudad de México C.P. 05348, Mexico

* Correspondence: rvargas@izt.uam.mx (R.V.); aij@xanum.uam.mx (A.I.-J.)

TABLE OF CONTENTS

CONTENT	PAGE
**Figure S1:** ^{1}H-NMR (500 MHz, $CDCl_3$) spectrum of compound **14a**	S4
**Figure S2:** ^{13}C-NMR (125 MHz, $CDCl_3$) spectrum of compound **14a**	S4
**Figure S3:** 2D-NMR (COSY) spectrum of compound **14a**	S5
**Figure S4:** 2D-NMR (HSQC-part I) spectrum of compound **14a**	S5
**Figure S5:** 2D-NMR (HSQC-part II) spectrum of compound **14a**	S6
**Figure S6:** 2D-NMR (HMBC-part I) spectrum of compound **14a**	S6
**Figure S7:** 2D-NMR (HMBC-part II) spectrum of compound **14a**	S7
**Figure S8:** 2D-NMR (HMBC-part III) spectrum of compound **14a**	S7
**Figure S9:** ^{1}H-NMR (500 MHz, $CDCl_3$) spectrum of compound **19a**	S8
**Figure S10:** ^{13}C-NMR (125 MHz, $CDCl_3$) spectrum of compound **19a**	S8
**Figure S11:** ^{19}F-NMR (500 MHz, $CDCl_3$) spectrum of compound **19a**	S9
**Figure S12:** 2D-NMR (COSY) spectrum of compound **19a**	S9
**Figure S13:** 2D-NMR (HSQC-part I) spectrum of compound **19a**	S10
**Figure S14:** 2D-NMR (HSQC-part II) spectrum of compound **19a**	S10
**Figure S15:** 2D-NMR (HMBC-part I) spectrum of compound **19a**	S11
**Figure S16:** 2D-NMR (HMBC-part II) spectrum of compound **19a**	S11
**Figure S17:** 2D-NMR (HMBC-part III) spectrum of compound **19a**	S12
**Figure S18:** HRMS (ESI^{+}-TOF) spectrum of compound **19a**	S12
**Figure S19:** FT-IR (ATR) spectrum of compound **19a**	S13
**Figure S20:** ^{1}H-NMR (500 MHz, $CDCl_3$) spectrum of compound **19b**	S13

**Figure S21:** ^{13}C-NMR (125 MHz, $CDCl_3$) spectrum of compound **19b**	S14
**Figure S22:** HRMS (ESI$^+$-TOF) spectrum of compound **19b**	S14
**Figure S23:** FT-IR (ATR) spectrum of compound **19b**	S15
**Figure S24:** ^{1}H-NMR (500 MHz, $CDCl_3$) spectrum of compound **19c**	S15
**Figure S25:** ^{13}C-NMR (125 MHz, $CDCl_3$) spectrum of compound **19c**	S16
**Figure S26:** HRMS (ESI$^+$-TOF) spectrum of compound **19c**	S16
**Figure S27:** FT-IR (ATR) spectrum of compound **19c**	S17
**Figure S28:** ^{1}H-NMR (500 MHz, $CDCl_3$) spectrum of compound **19d**	S17
**Figure S29:** ^{13}C-NMR (125 MHz, $CDCl_3$) spectrum of compound **19d**	S18
**Figure S30:** HRMS (ESI$^+$-TOF) spectrum of compound **19d**	S18
**Figure S31:** FT-IR (ATR) spectrum of compound **19d**	S19
**Figure S32:** ^{1}H-NMR (500 MHz, $CDCl_3$) spectrum of compound **19e**	S19
**Figure S33:** ^{13}C-NMR (125 MHz, $CDCl_3$) spectrum of compound **19e**	S20
**Figure S34:** HRMS (ESI$^+$-TOF) spectrum of compound **19**e	S20
**Figure S35:** FT-IR (ATR) spectrum of compound **19e**	S21
**Figure S36:** ^{1}H-NMR (500 MHz, $CDCl_3$) spectrum of compound **19f**	S21
**Figure S37:** ^{13}C-NMR (125 MHz, $CDCl_3$) spectrum of compound **19f**	S22
**Figure S38:** HRMS (ESI$^+$-TOF) spectrum of compound **19f**	S22
**Figure S39:** FT-IR (ATR) spectrum of compound **19f**	S23
**Figure S40:** ^{1}H-NMR (500 MHz, $CDCl_3$) spectrum of compound **19g**	S23
**Figure S41:** ^{13}C-NMR (125 MHz, $CDCl_3$) spectrum of compound **19g**	S24
**Figure S42:** HRMS (ESI$^+$-TOF) spectrum of compound **19g**	S24
**Figure S43**: FT-IR (ATR) spectrum of compound **19g**	S25
**Figure S44:** ^{1}H-NMR (500 MHz, $CDCl_3$) spectrum of compound **19h**	S25
**Figure S45:** ^{13}C-NMR (125 MHz, $CDCl_3$) spectrum of compound **19h**	S26
**Figure S46:** HRMS (ESI$^+$-TOF) spectrum of compound **19h**	S26
**Figure S47:** FT-IR (ATR) spectrum of compound **19h**	S27
**Figure S48:** ^{1}H-NMR (500 MHz, $CDCl_3$) spectrum of compound **19i**	S27
**Figure S49:** ^{13}C-NMR (125 MHz, $CDCl_3$) spectrum of compound **19i**	S28
**Figure S50:** HRMS (ESI$^+$-TOF) spectrum of compound **19i**	S28
**Figure S51:** FT-IR (ATR) spectrum of compound **19i**	S29
**Figure S52:** ^{1}H-NMR (500 MHz, $CDCl_3$) spectrum of compound **19j**	S29
**Figure S53:** ^{13}C-NMR (125 MHz, $CDCl_3$) spectrum of compound **19j**	S30
**Figure S54:** 2D-NMR (HMBC-part I) spectrum of compound **19j**	S30
**Figure S55:** 2D-NMR (HMBC-part II) spectrum of compound **19j**	S31
**Figure S56:** ^{19}F-NMR (500 MHz, $CDCl_3$) spectrum of compound **19j**	S31
**Figure S57:** HRMS (ESI$^+$-TOF) spectrum of compound **19j**	S32
**Figure S58:** FT-IR (ATR) spectrum of compound **19j**	S32
**Figure S59:** ^{1}H-NMR (500 MHz, $CDCl_3$) spectrum of compound **19k**	S33
**Figure S60:** ^{13}C-NMR (125 MHz, $CDCl_3$) spectrum of compound **19k**	S33
**Figure S61:** 2D-NMR (HMBC-part I) spectrum of compound **19k**	S34
**Figure S62:** 2D-NMR (HMBC-part II) spectrum of compound **19k**	S34
**Figure S63:** ^{19}F-NMR (500 MHz, $CDCl_3$) spectrum of compound **19k**	S35
**Figure S64:** HRMS (ESI$^+$-TOF) spectrum of compound **19k**	S35
**Figure S65:** FT-IR (ATR) spectrum of compound **19k**	S36
**Figure S66:** ^{1}H-NMR (500 MHz, $CDCl_3$) spectrum of compound **19l**	S36
**Figure S67:** ^{13}C-NMR (125 MHz, $CDCl_3$) spectrum of compound **19l**	S37
**Figure S68:** 2D-NMR (HMBC-part I) spectrum of compound **19l**	S37
**Figure S69:** 2D-NMR (HMBC-part II) spectrum of compound **19l**	S38
**Figure S70:** ^{19}F-NMR (500 MHz, $CDCl_3$) spectrum of compound **19l**	S38
**Figure S71:** HRMS (ESI$^+$-TOF) spectrum of compound **19l**	S39
**Figure S72:** FT-IR (ATR) spectrum of compound **19l**	S39
**Figure S73:** Structural Representation of the Main Protease of SARS-CoV-2 (M_{pro}/nsp5).	S40
**Figure S74:** Structural Depiction of the SARS-CoV-2 Replicase (nsp7).	S41
**Figure S75:** Structural Representation of the SARS-CoV-2 Polymerase (nsp12).	S42
**Figure S76:** Structural Depiction of the SARS-CoV-2 Helicase (nsp13).	S43

**Figure S77:** Illustrates the structure representation of the spike protein using its *N*-terminal domain and the receptor-binding domain of the SARS-CoV-2 virus.	S44
**Figure S78**: Analysis of Homology and Mutations in NTD and RBD Variants.	S45
**Figure S79:** Bubble Plot of Binding Energy and Two-Dimensional Protein-Ligand Interaction.	S45
**Figure S80:** Comprehensive Methodology.	S46
**Figure S81:** Superposition of the 8D35 (yellow) and 9DW6 (red) crystal structures.	S47
**Table S1:** ADMETox Properties of compounds **19d** and **19i**.	S48
**Table S2:** Active Site Match Using PrankWeb, GRaSP, and PDBsum.	S49
**Table S3:** Cavity Size.	S50
**Table S4:** Better link energies with good population.	S51
**Table S5:** Comparative binding affinities of chloroquine, **19d**, and **19i** against selected SARS-CoV-2 targets.	S52
**Table S6:** Summary of Molecular Dynamics Properties.	S53
**Table S7:** NTDα MM/GBSA Decomposition.	S54
**Table S8:** NTDα Per-Amino Acid GB Δ_TDC.	S54
**Table S9:** NTDo MM/GBSA Decomposition.	S55
**Table S10:** NTDo Per-Amino Acid GB Δ_TDC.	S55
**Table S11:** nsp5 MM/GBSA Decomposition.	S56
**Table S12:** nsp5 Per-Amino Acid GB Δ_TDC.	S56
**Supplementary Movie 1**	S56
**Supplementary Movie 2**	S56

N^1-((4-benzyl-5-morpholinooxazol-2-yl)(4-fluorophenyl)methyl)-N^2-(7-chloroquinolin-4-yl)ethane-1,2-diamine

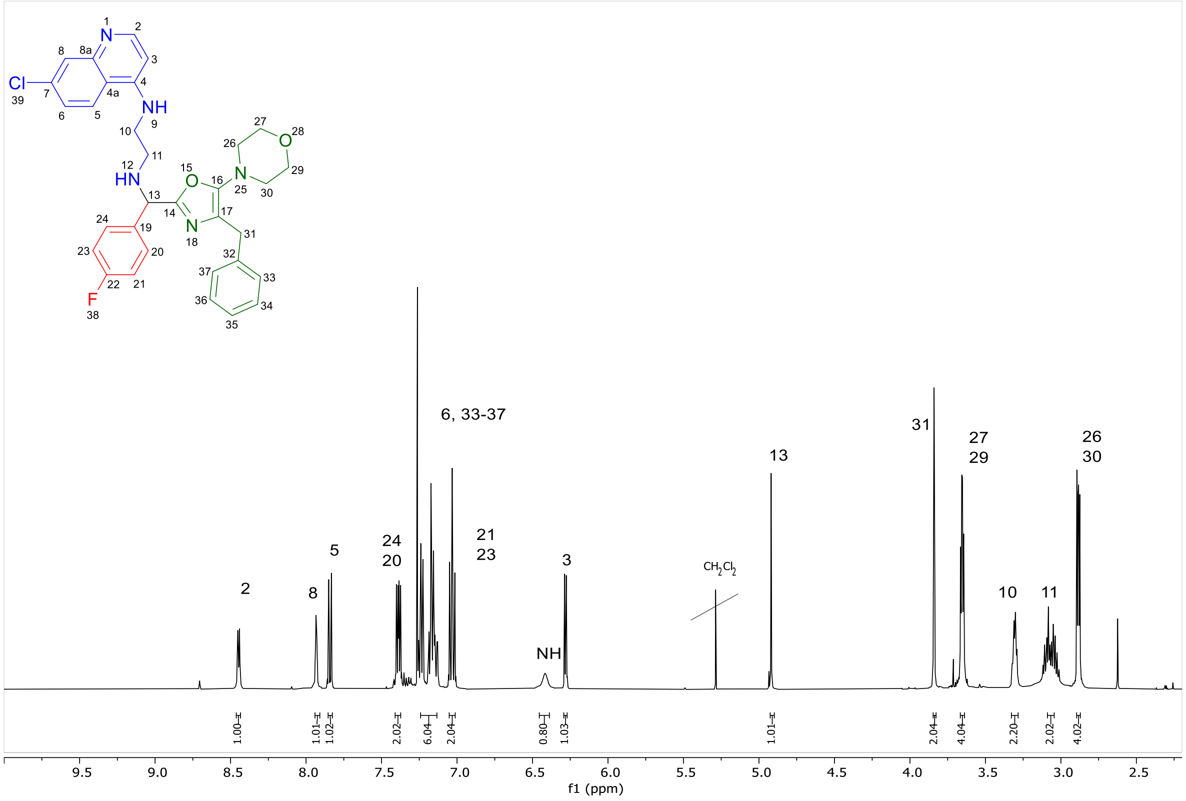

Figure S1: ^{1}H-NMR (500 MHz, $CDCl_3$) spectrum of compound **14a**

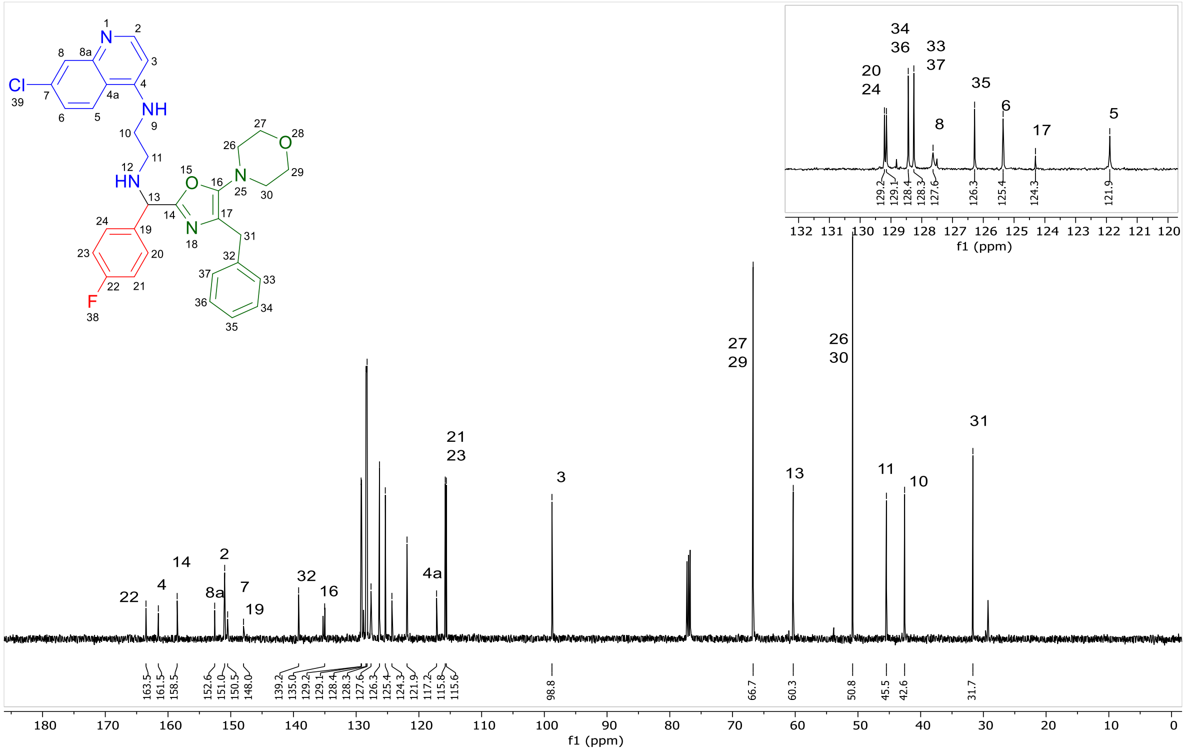

Figure S2: ^{13}C-NMR (125 MHz, $CDCl_3$) spectrum of compound **14a**

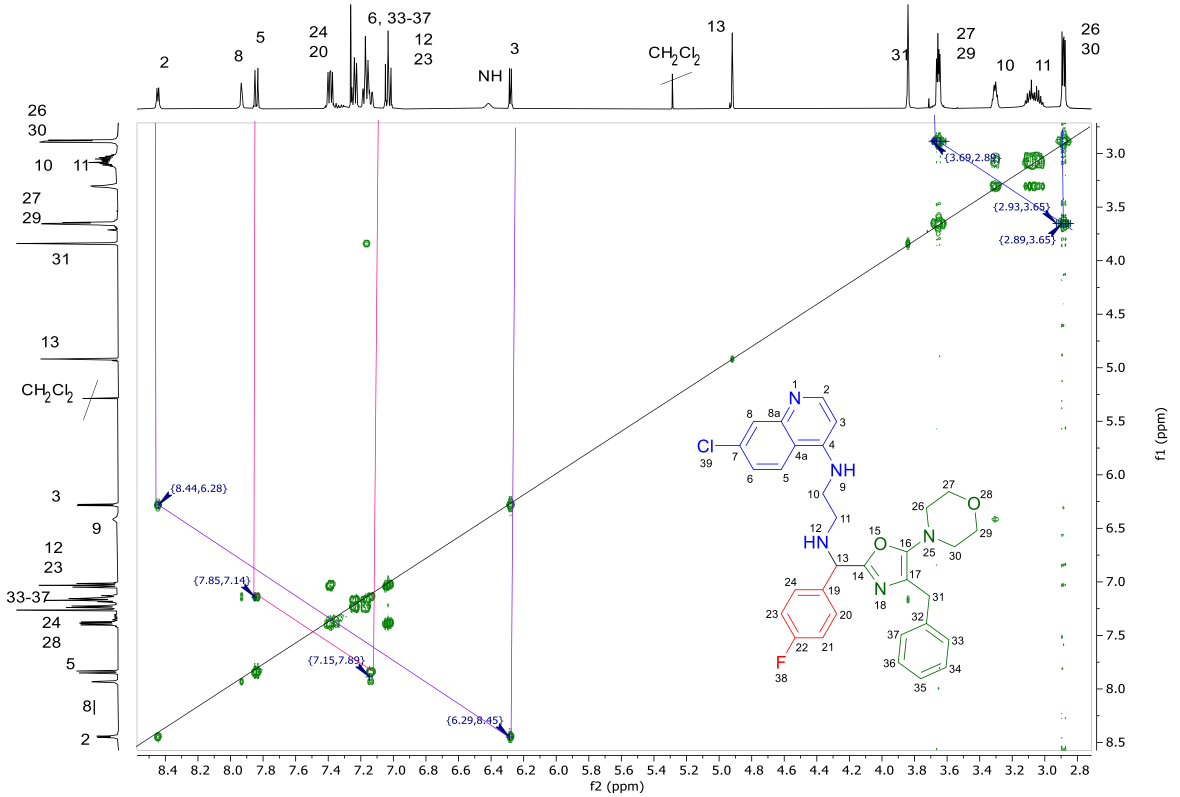

Figure S3: 2D-NMR (COSY) spectrum of compound **14a**

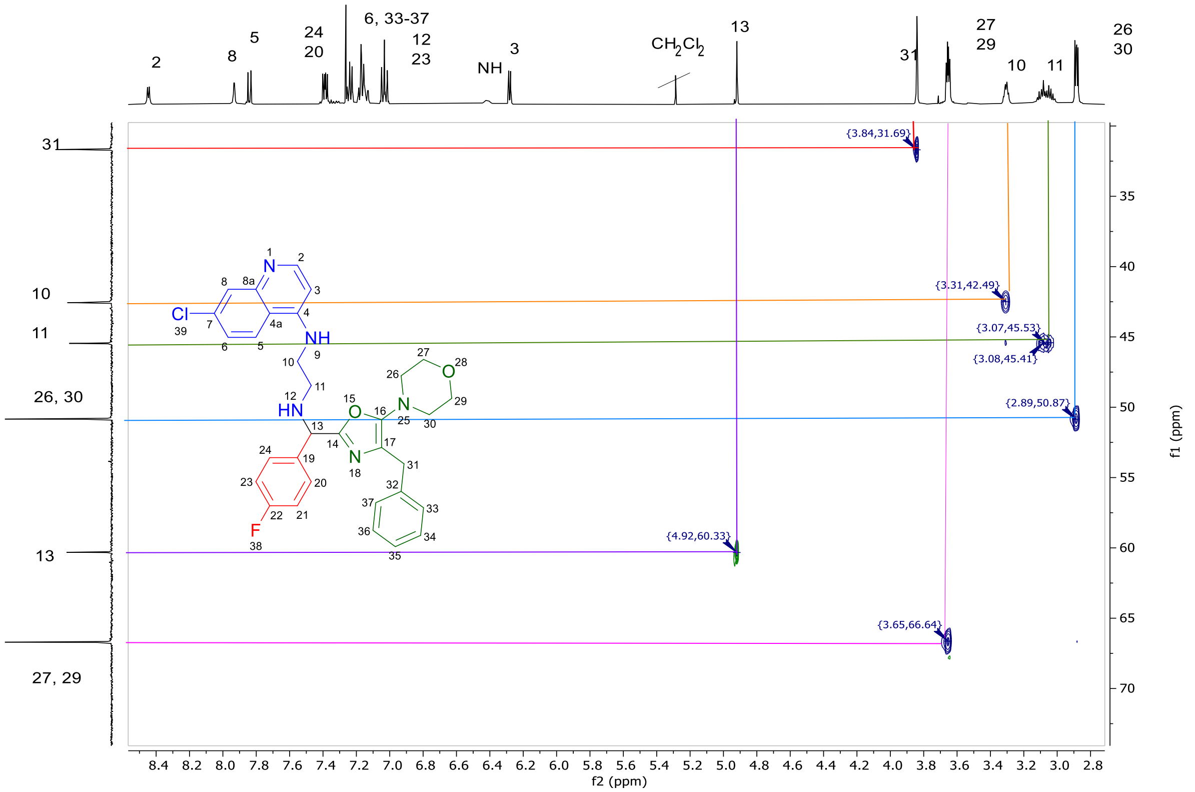

Figure S4: 2D-NMR (HSQC-part I) spectrum of compound **14a**

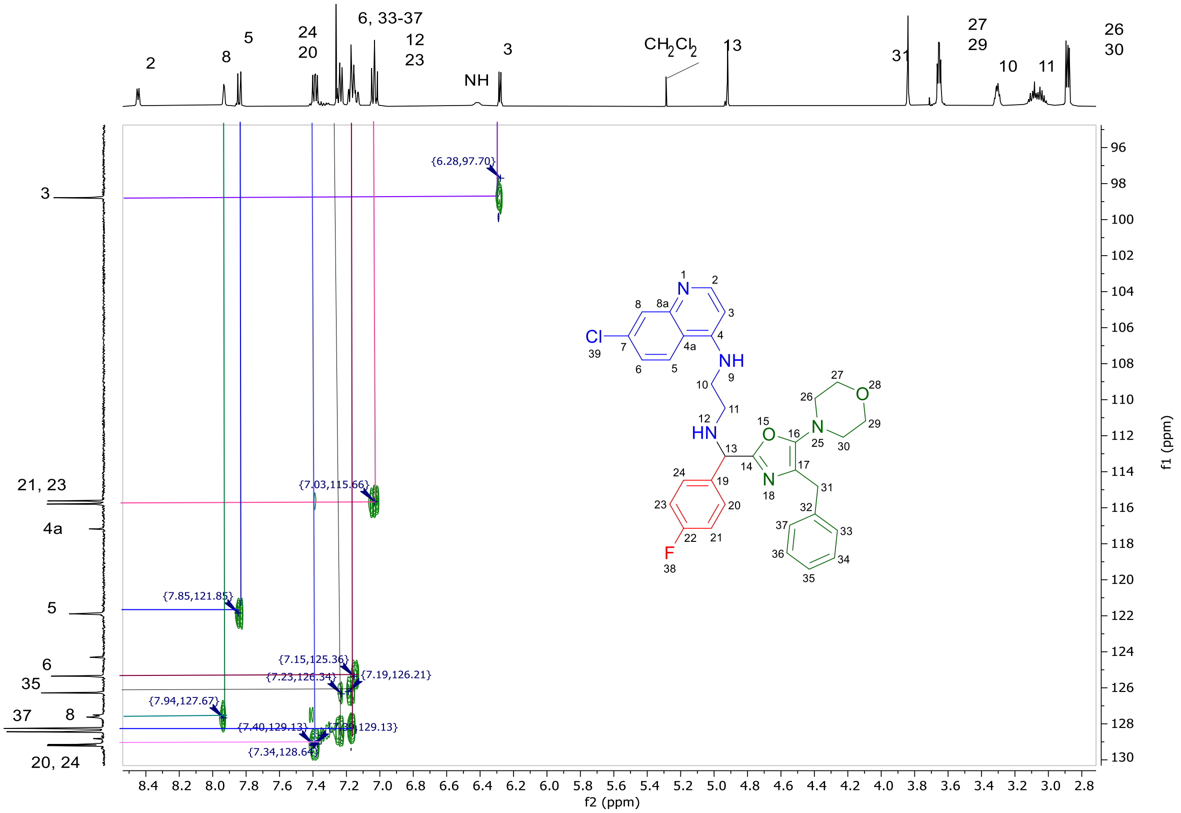

Figure S5: 2D-NMR (HSQC-part II) spectrum of compound **14a**

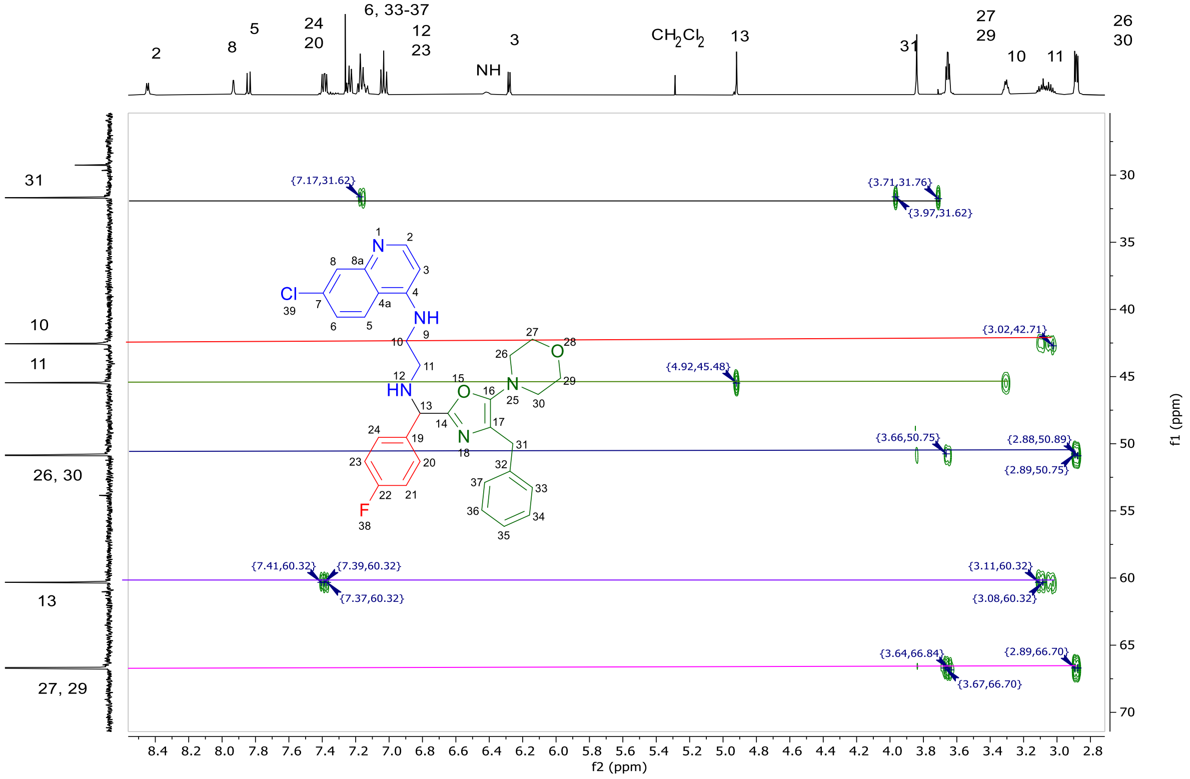

Figure S6: 2D-NMR (HMBC-part I) spectrum of compound **14a**

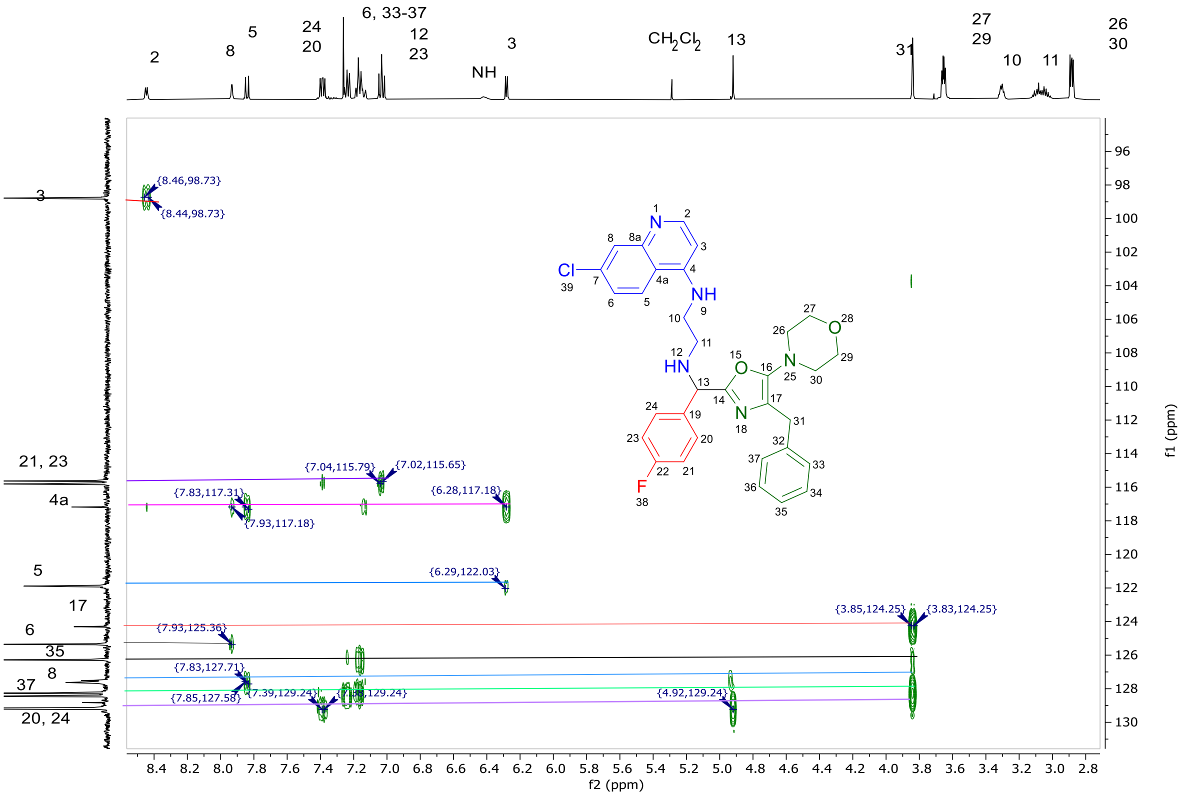

Figure S7: 2D-NMR (HMBC-part II) spectrum of compound **14a**

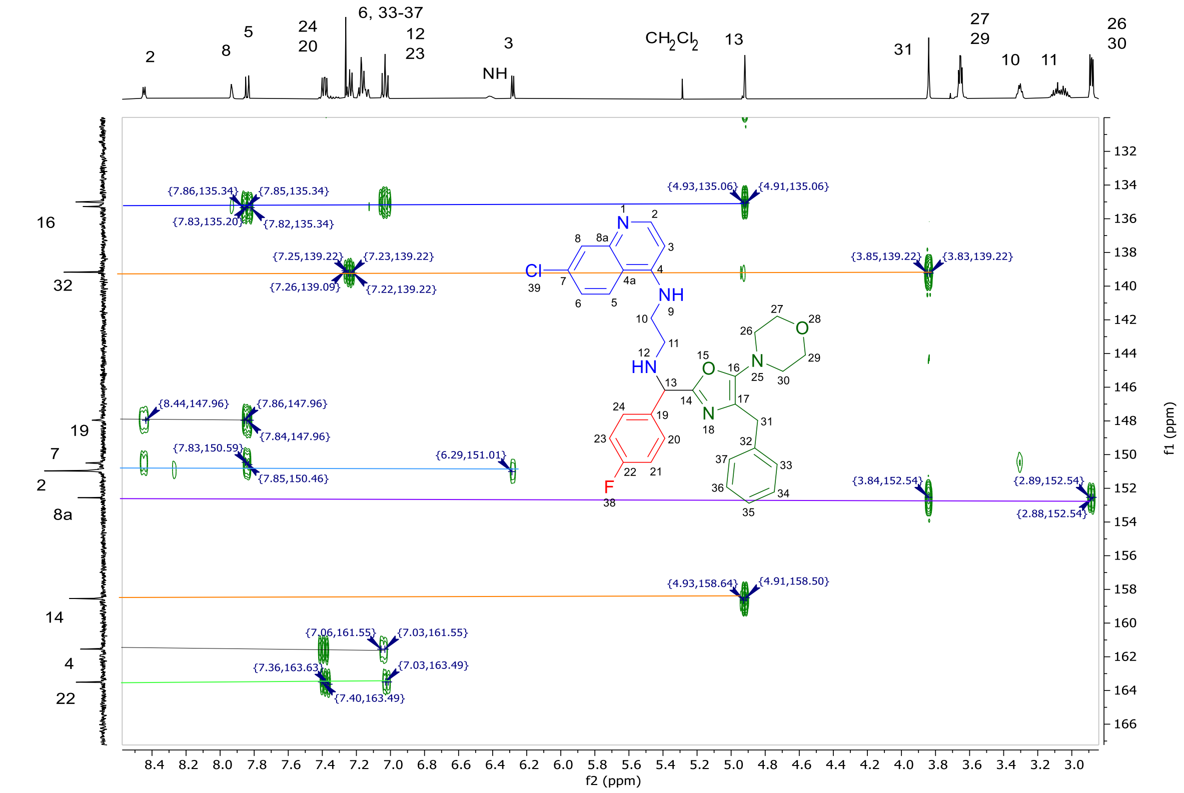

Figure S8: 2D-NMR (HMBC-part III) spectrum of compound **14a**

2-benzyl-6-(2-((7-chloroquinolin-4-yl)amino)ethyl)-7-(4-fluorophenyl)-3-morpholino-6,7-dihydro-5*H*-pyrrolo[3,4-*b*]pyridin-5-one **19a**

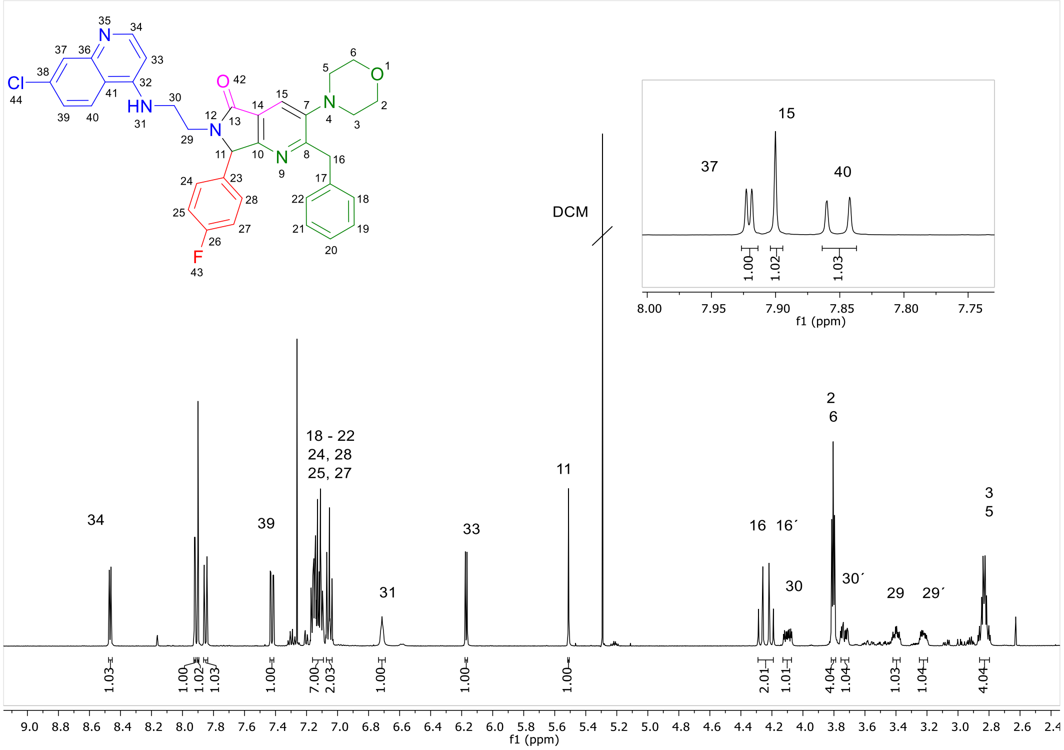

Figure S9: ^{1}H-NMR (500 MHz, $CDCl_3$) spectrum of compound **19a**

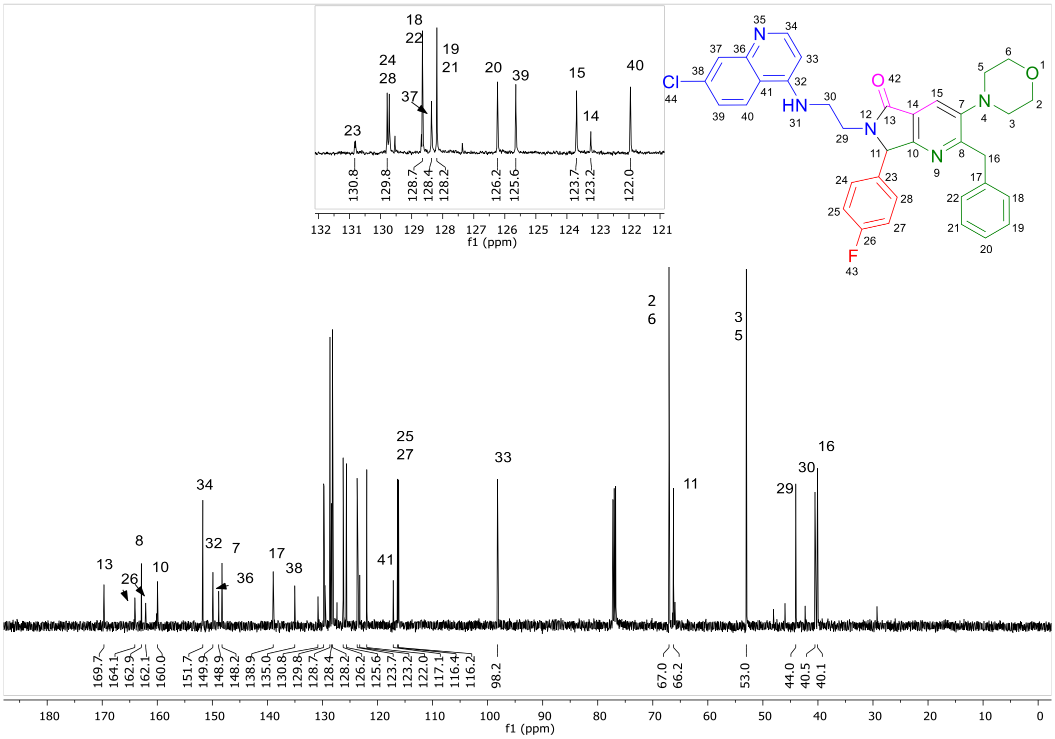

Figure S10: ^{13}C-NMR (125 MHz, $CDCl_3$) spectrum of compound **19a**

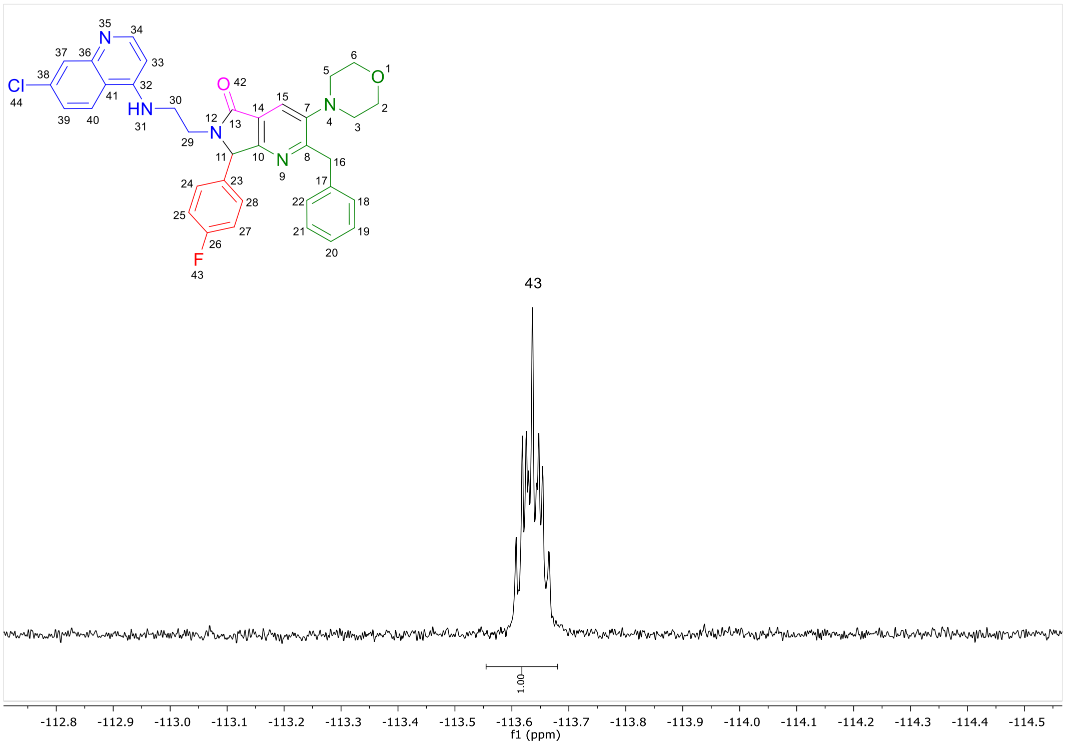

Figure S11: ^{19}F-NMR (500 MHz, $CDCl_3$) spectrum of compound **19a**

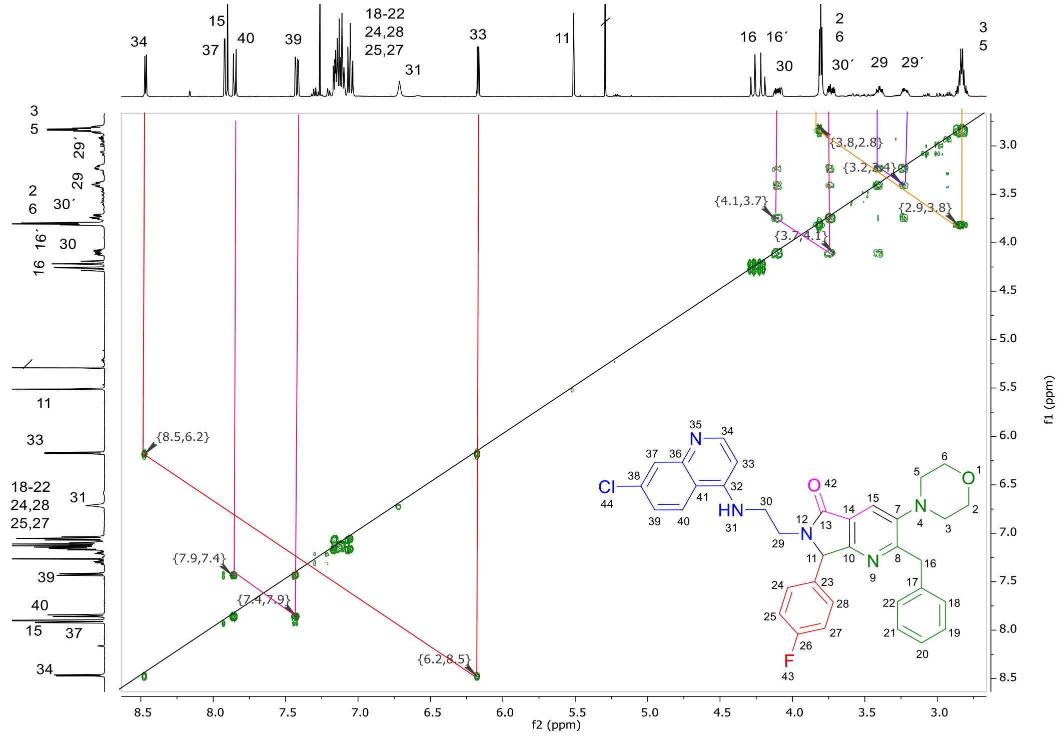

Figure S12: 2D-NMR (COSY) spectrum of compound **19a**

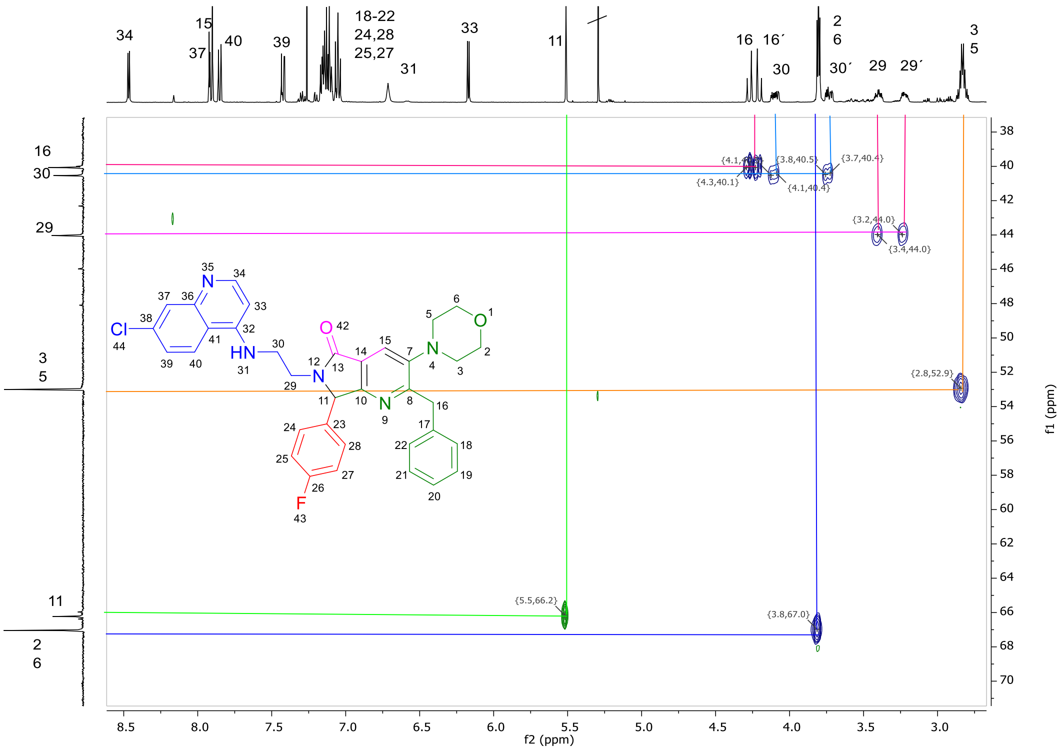

Figure S13: 2D-NMR (HSQC-part I) spectrum of compound **19a**

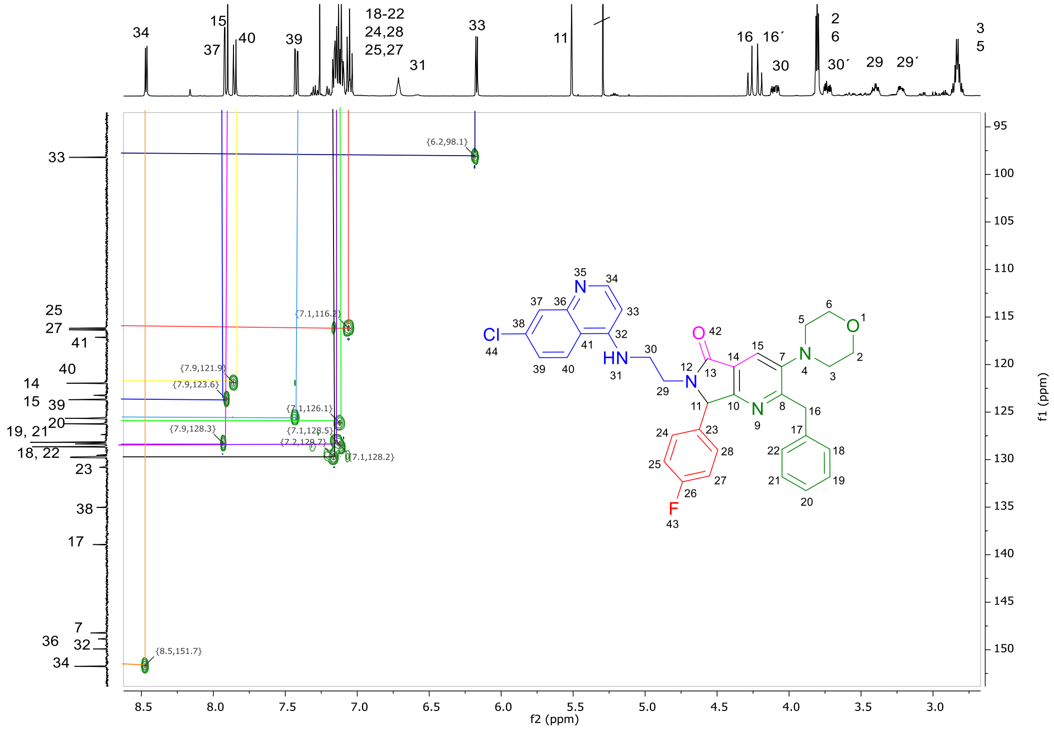

Figure S14: 2D-NMR (HSQC-part II) spectrum of compound **19a**

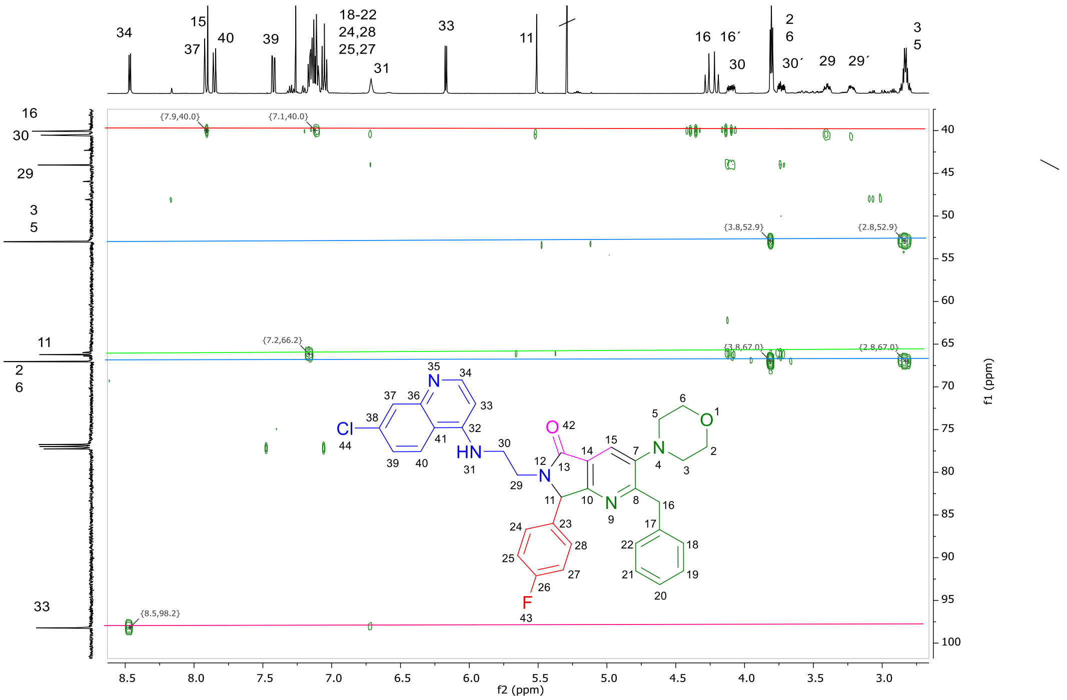

Figure S15: 2D-NMR (HMBC-part I) spectrum of compound **19a**

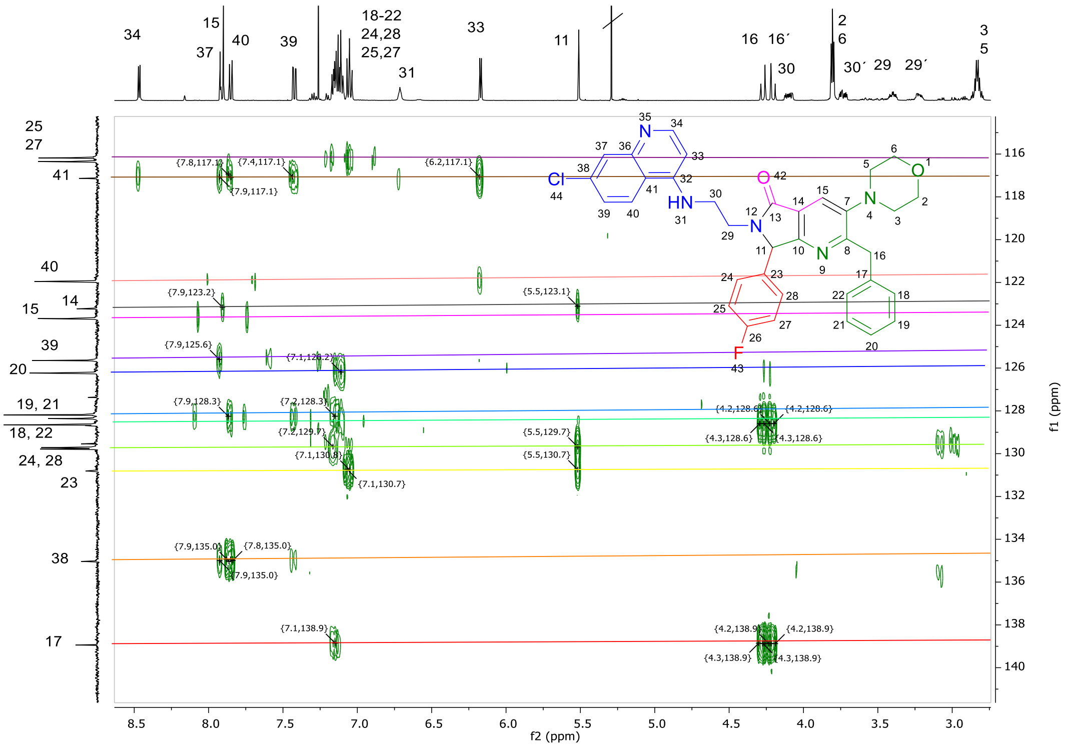

Figure S16: 2D-NMR (HMBC-part II) spectrum of compound **19a**

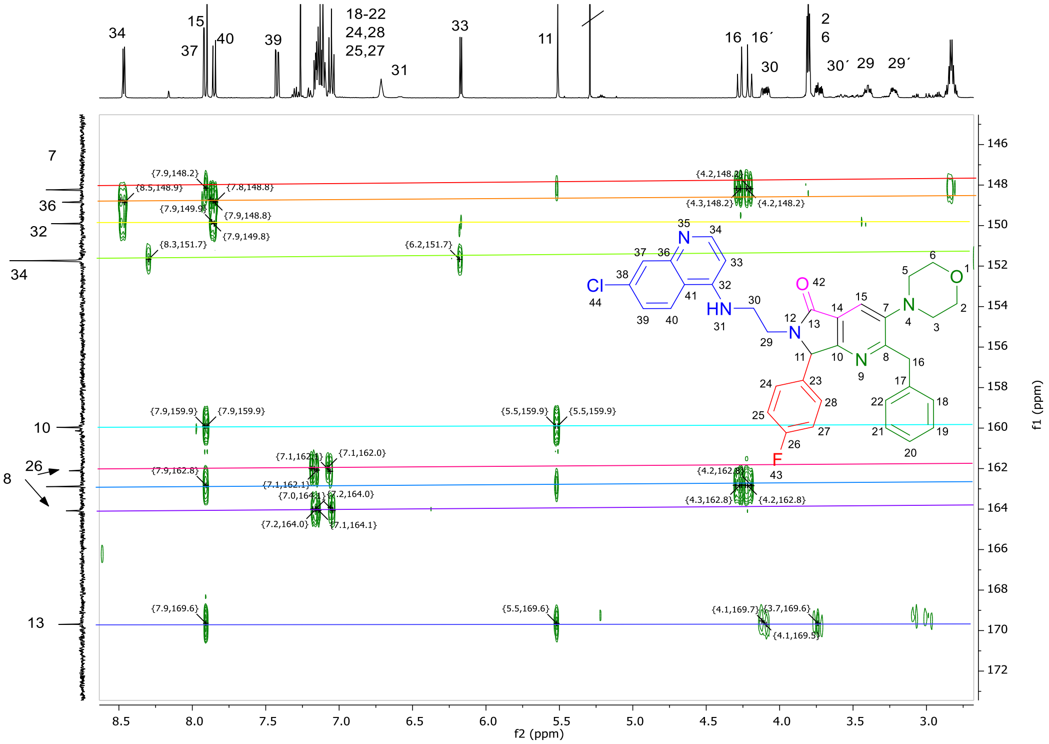

Figure S17: 2D-NMR (HMBC-part III) spectrum of compound **19a**

Mass Spectrum SmartFormula Report

Acquisition Parameter

Source Type	ESI	Ion Polarity	Positive	Set Nebulizer	0.5 Bar
Focus	Active	Set Capillary	4500 V	Set Dry Heater	150 °C
Scan Begin	50 m/z	Set End Plate Offset	-500 V	Set Dry Gas	4.0 l/min
Scan End	3000 m/z	Set Charging Voltage	0 V	Set Divert Valve	Waste
		Set Corona	0 nA	Set APCI Heater	0 °C

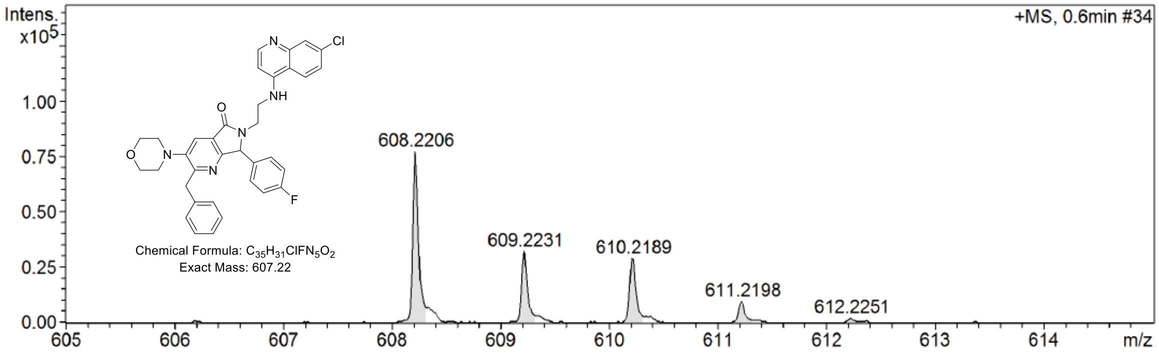

Meas. m/z	#	Ion Formula	Sum Formula	m/z	err [ppm]	mSigma	# Sigma
608.2206	1	C35H32ClFN5O2	C35 H31 Cl F N5 O2	608.2223	2.8	14.5	1

Figure S18: HRMS (ESI$^+$-TOF) spectrum of compound **19a**

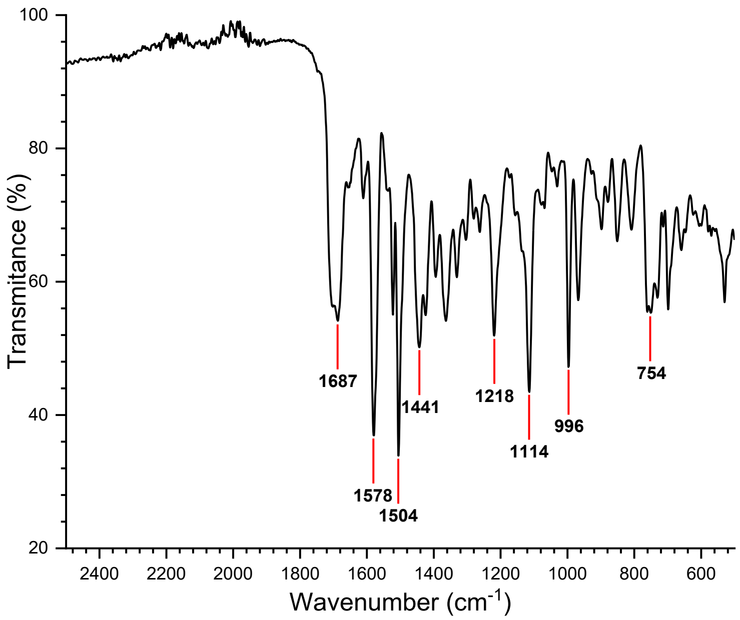

Figure S19: FT-IR (ATR) spectrum of compound **19a**

2-benzyl-6-(3-((7-chloroquinolin-4-yl)amino)propyl)-7-(4-fluorophenyl)-3-morpholino-6,7-dihydro-5*H*-pyrrolo[3,4-*b*]pyridin-5-one **19b**

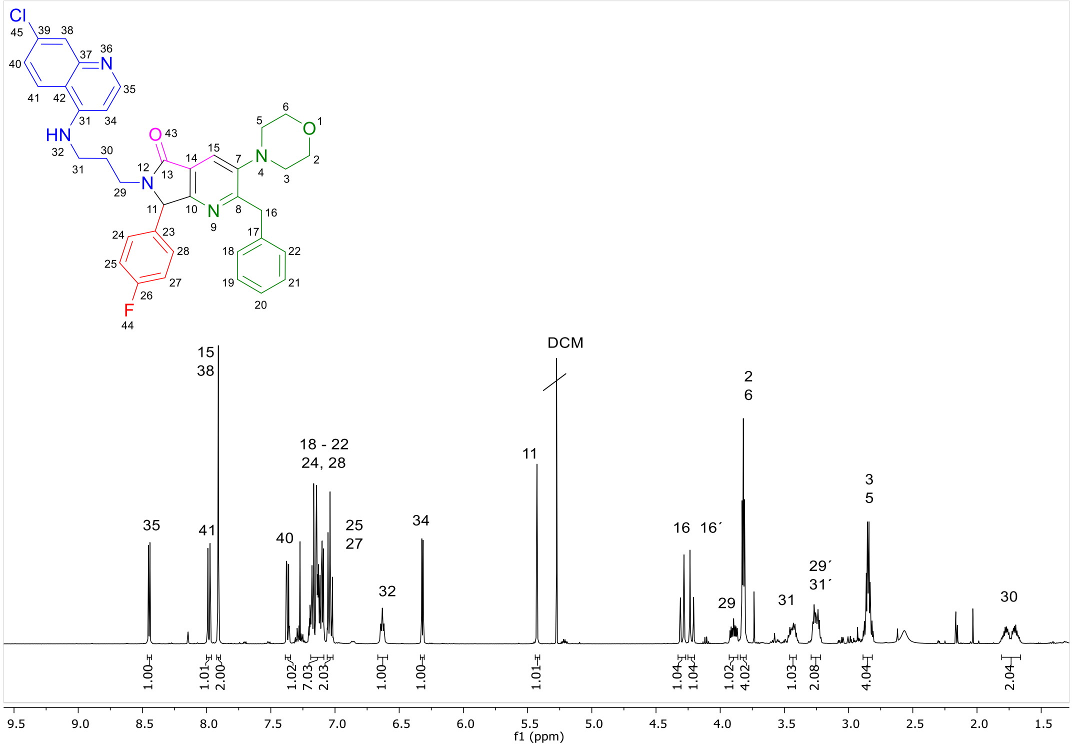

Figure S20: ^{1}H-NMR (500 MHz, $CDCl_3$) spectrum of compound **19b**

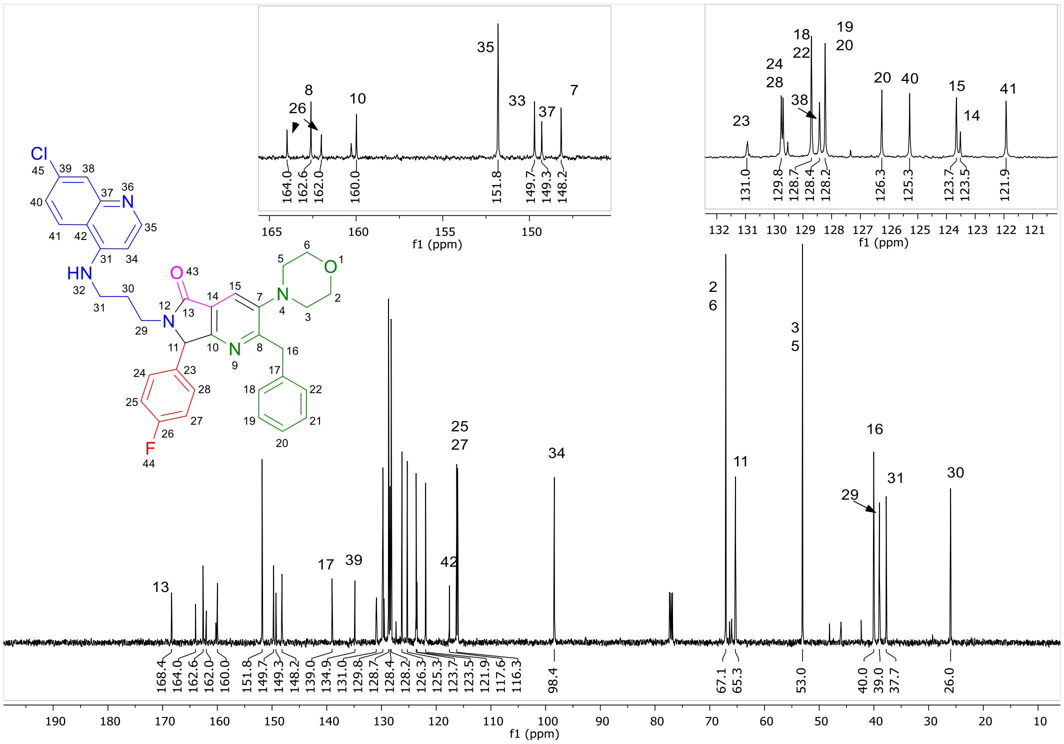

Figure S21: ^{13}C-NMR (125 MHz, $CDCl_3$) spectrum of compound **19b**

Mass Spectrum SmartFormula Report

Acquisition Parameter

Source Type	ESI	Ion Polarity	Positive	Set Nebulizer	0.3 Bar
Focus	Active	Set Capillary	4500 V	Set Dry Heater	180 °C
Scan Begin	50 m/z	Set End Plate Offset	-500 V	Set Dry Gas	4.0 l/min
Scan End	3000 m/z	Set Charging Voltage	0 V	Set Divert Valve	Waste
		Set Corona	0 nA	Set APCI Heater	0 °C

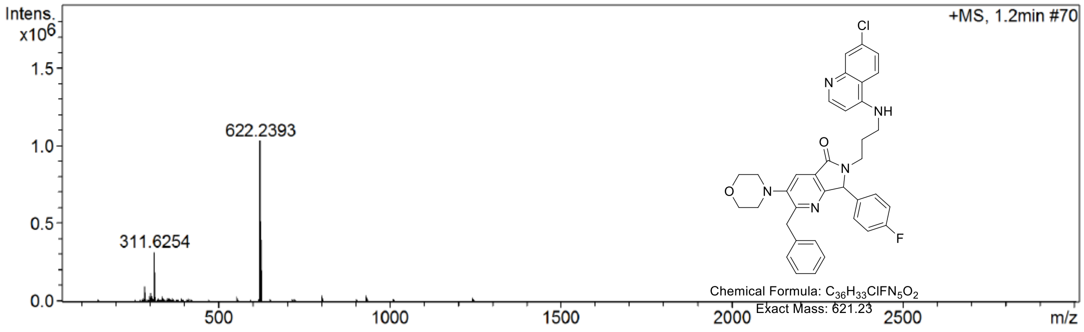

Meas. m/z	#	Ion Formula	Sum Formula	m/z	err [ppm]	mSigma	# Sigma	Score
556.2463	1	C29H36ClFN5O3	C29 H35 Cl F N5 O3	556.2485	4.1	12.7	1	47.98
	2	C32H35ClN5O2	C32 H34 Cl N5 O2	556.2474	2.0	16.1	2	100.00
	3	C26H37ClF2N5O4	C26 H36 Cl F2 N5 O4	556.2497	6.1	23.6	3	13.15
	4	C25H42Cl2F2N3O4	C25 H41 Cl2 F2 N3 O4	556.2515	9.4	129.7	4	0.02
	5	C27H38Cl2F2N5O	C27 H37 Cl2 F2 N5 O	556.2416	8.4	130.5	5	0.04
	6	C26H40Cl2N5O4	C26 H39 Cl2 N5 O4	556.2452	1.9	132.0	6	1.13
	7	C28H41Cl2FN3O3	C28 H40 Cl2 F N3 O3	556.2504	-7.3	133.2	7	0.07
	8	C31H40Cl2N3O2	C31 H39 Cl2 N3 O2	556.2492	5.3	138.8	8	0.18
	9	C35H31FN5O	C35 H30 F N5 O	556.2507	8.0	166.4	9	0.01
622.2393	1	C36H34ClFN5O2	C36 H33 Cl F N5 O2	622.2380	2.1	9.6	1	100.00

Figure S22: HRMS (ESI$^+$-TOF) spectrum of compound **19b**

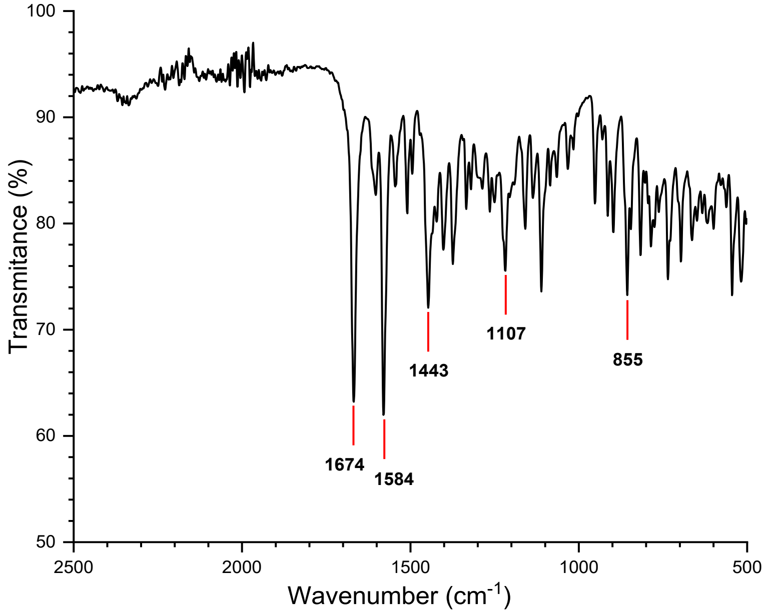

Figure S23: FT-IR (ATR) spectrum of compound **19b**

2-benzyl-6-(2-((7-chloroquinolin-4-yl)amino)ethyl)-7-(4-fluorophenyl)-3-(piperidin-1-yl)-6,7-dihydro-5*H*-pyrrolo[3,4-*b*]pyridin-5-one **19c**

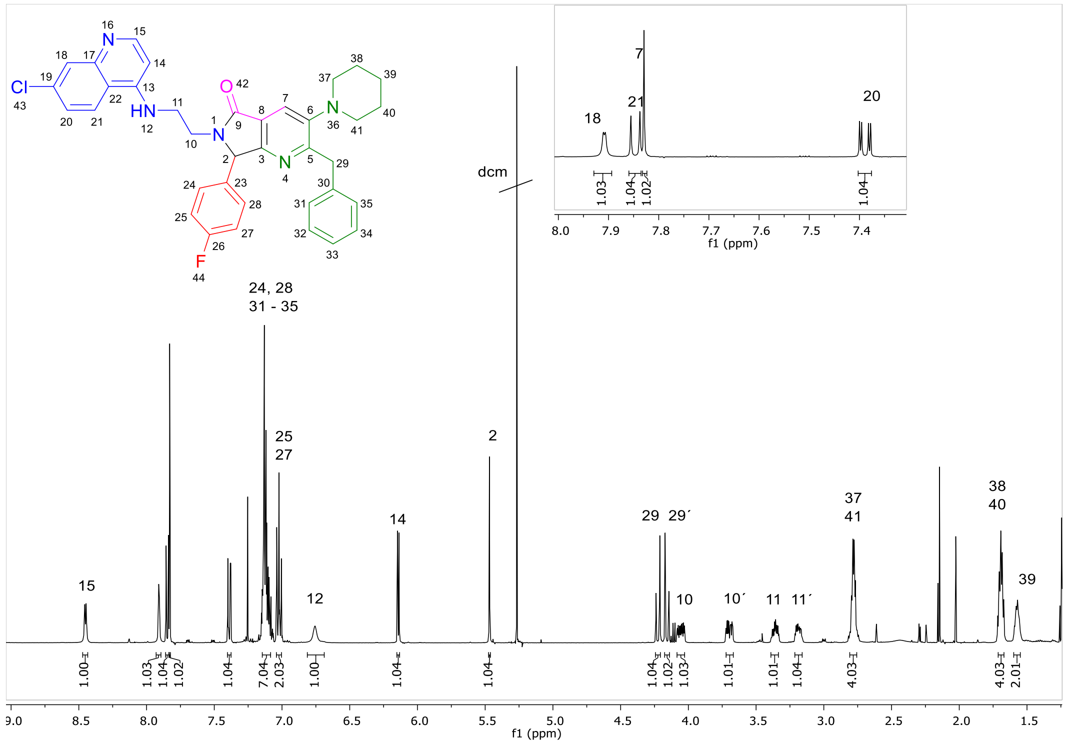

Figure S24: ^{1}H-NMR (500 MHz, $CDCl_3$) spectrum of compound **19c**

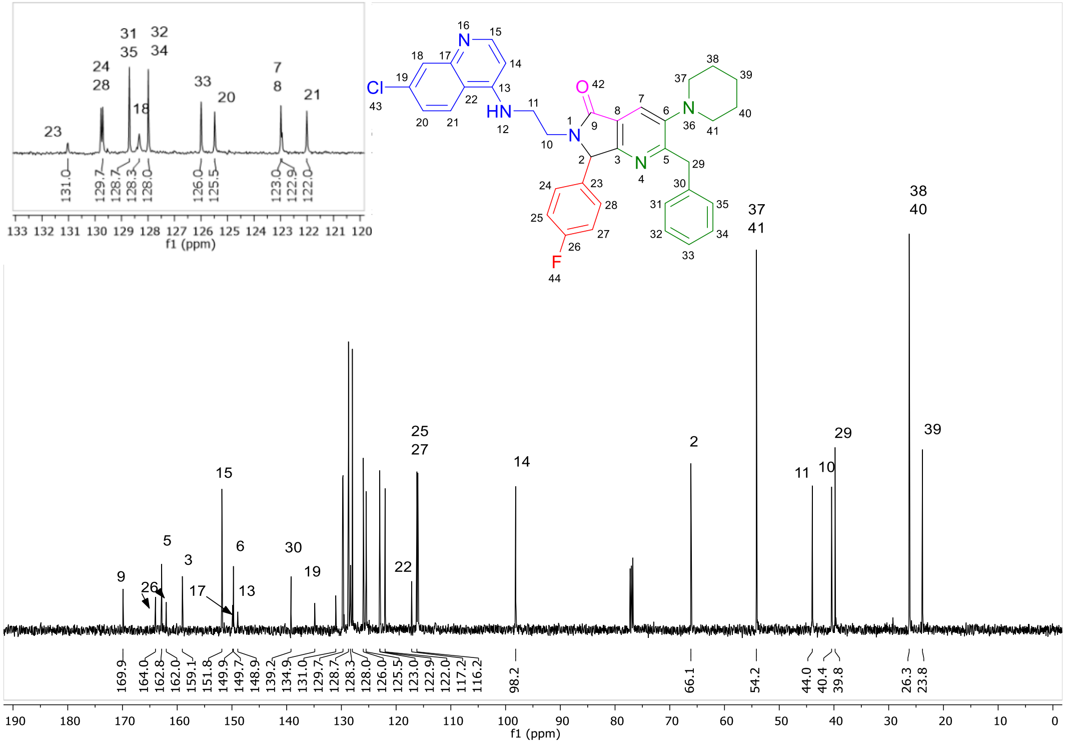

Figure S25: ^{13}C-NMR (125 MHz, $CDCl_3$) spectrum of compound **19c**

Mass Spectrum SmartFormula Report

Acquisition Parameter

Source Type	ESI	Ion Polarity	Positive	Set Nebulizer	0.5 Bar
Focus	Active	Set Capillary	4500 V	Set Dry Heater	150 °C
Scan Begin	50 m/z	Set End Plate Offset	-500 V	Set Dry Gas	4.0 l/min
Scan End	3000 m/z	Set Charging Voltage	0 V	Set Divert Valve	Waste
		Set Corona	0 nA	Set APCI Heater	0 °C

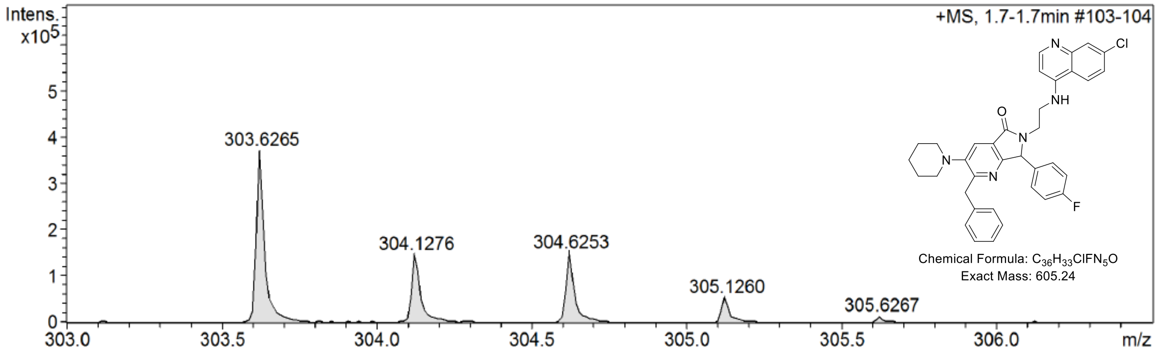

Meas. m/z	#	Ion Formula	Sum Formula	m/z	err [ppm]	mSigma	# Sigma
303.6265	1	C36H35ClFN5O	C36 H35 Cl F N5 O	303.6252	-4.4	7.1	1
606.2408	1	C36H34ClFN5O	C36 H33 Cl F N5 O	606.2430	-3.7	12.6	1

Figure S26: HRMS (ESI$^+$-TOF) spectrum of compound **19c**

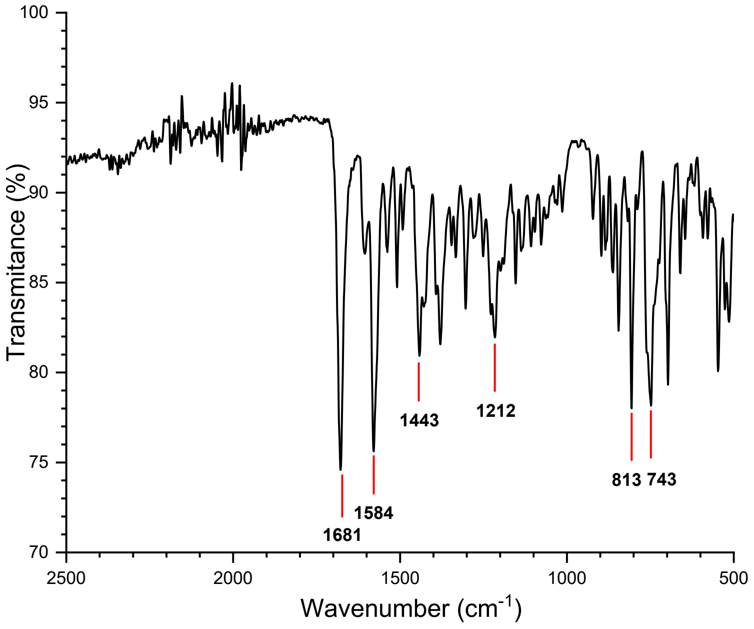

Figure S27: FT-IR (ATR) spectrum of compound **19c**

2-benzyl-6-(2-((7-chloroquinolin-4-yl)amino)ethyl)-3-(diethylamino)-7-(4-fluorophenyl)-6,7-dihydro-5*H*-pyrrolo[3,4-*b*]pyridin-5-one **19d**

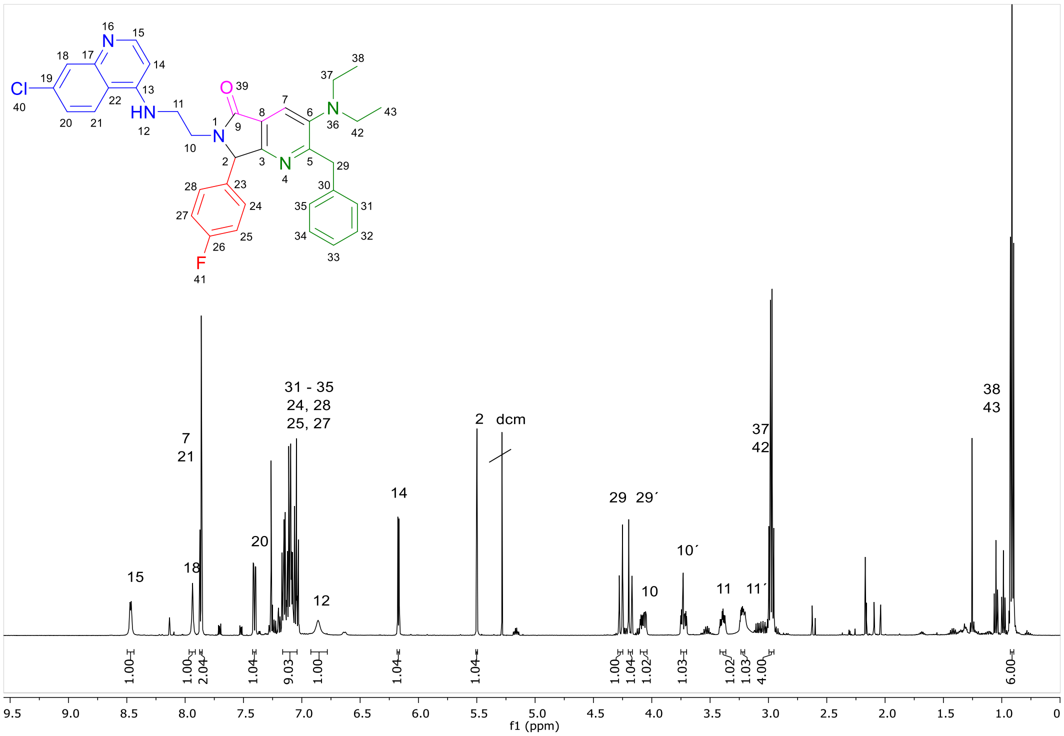

Figure S28: ^{1}H-NMR (500 MHz, $CDCl_3$) spectrum of compound **19d**

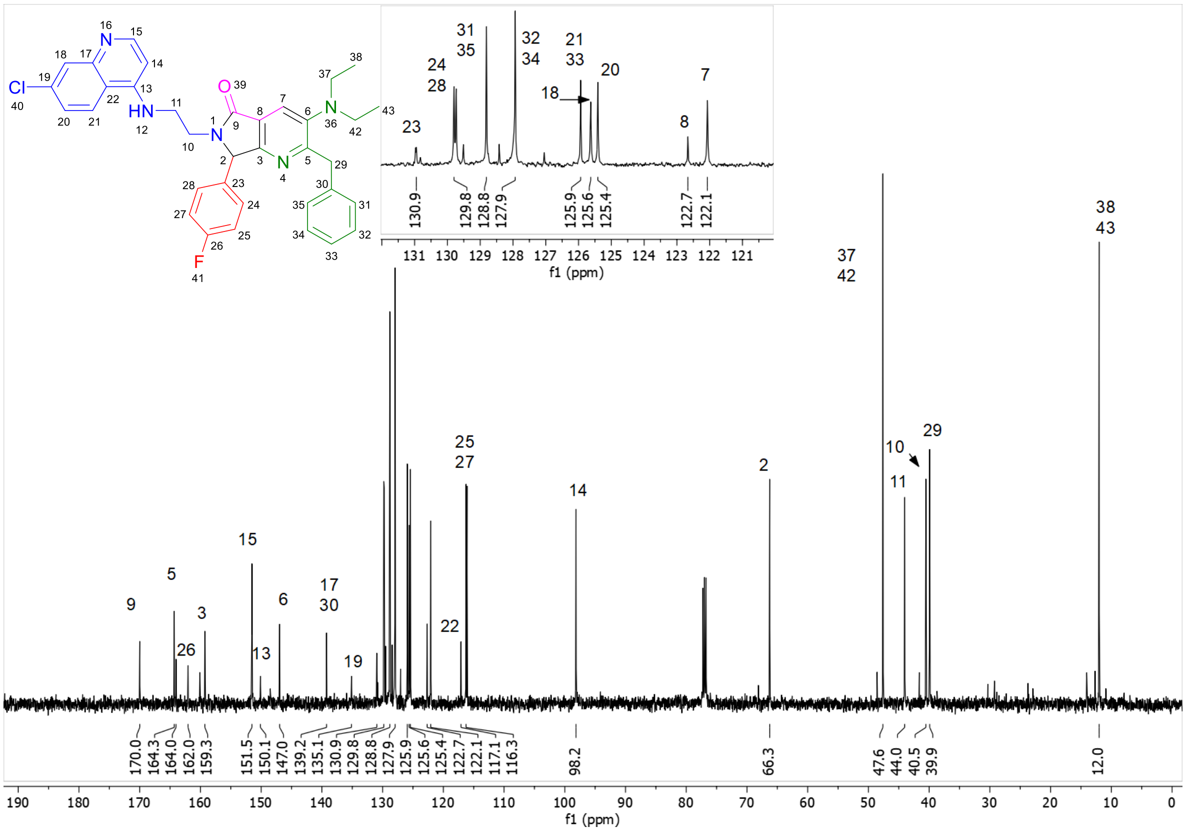

Figure S29: ^{13}C-NMR (125 MHz, $CDCl_3$) spectrum of compound **19d**

Mass Spectrum SmartFormula Report

Acquisition Parameter

Source Type	ESI	Ion Polarity	Positive	Set Nebulizer	0.5 Bar
Focus	Active	Set Capillary	4500 V	Set Dry Heater	150 °C
Scan Begin	50 m/z	Set End Plate Offset	-500 V	Set Dry Gas	4.0 l/min
Scan End	3000 m/z	Set Charging Voltage	0 V	Set Divert Valve	Waste
		Set Corona	0 nA	Set APCI Heater	0 °C

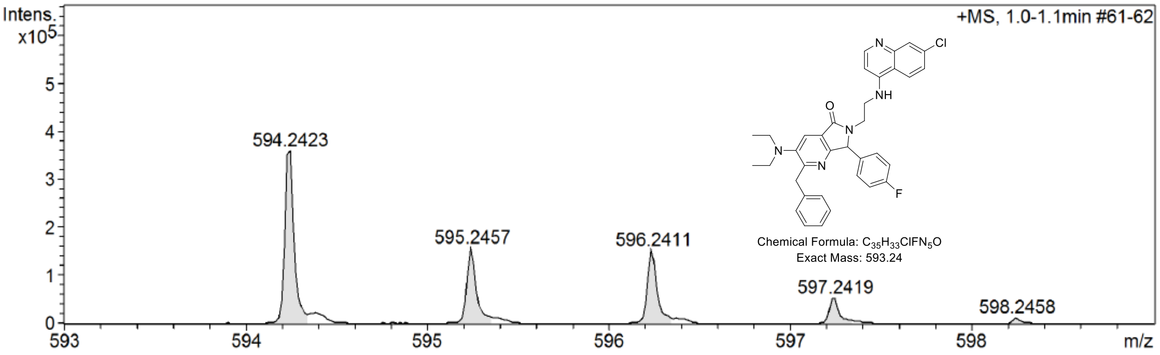

Meas. m/z	#	Ion Formula	Sum Formula	m/z	err [ppm]	|err| [mDa]	mSigma	# Sigma
297.6272	1	C35H35ClFN5O	C35 H33 Cl F N5 O	297.6252	-6.8	2.0	28.7	2
594.2423	1	C35H34ClFN5O		594.2430	-1.2	0.7	18.7	1

Figure S30: HRMS (ESI$^+$-TOF) spectrum of compound **19d**

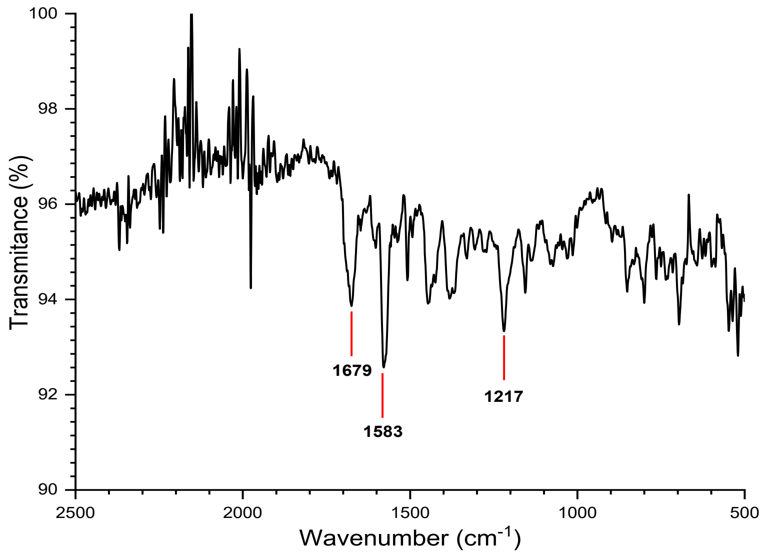

Figure S31: FT-IR (ATR) spectrum of compound **19d**

2-benzyl-6-(2-((7-chloroquinolin-4-yl)amino)ethyl)-7-(2-fluorophenyl)-3-morpholino-6,7-dihydro-5*H*-pyrrolo[3,4-*b*]pyridin-5-one **19e**

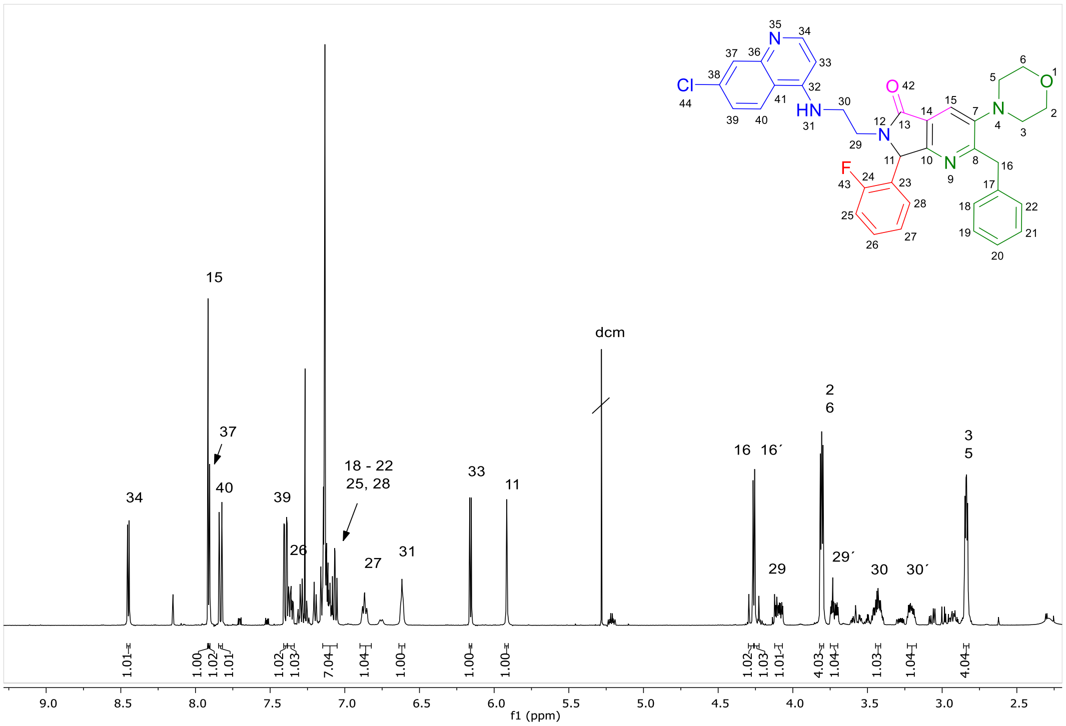

Figure S32: ^{1}H-NMR (500 MHz, $CDCl_3$) spectrum of compound **19e**

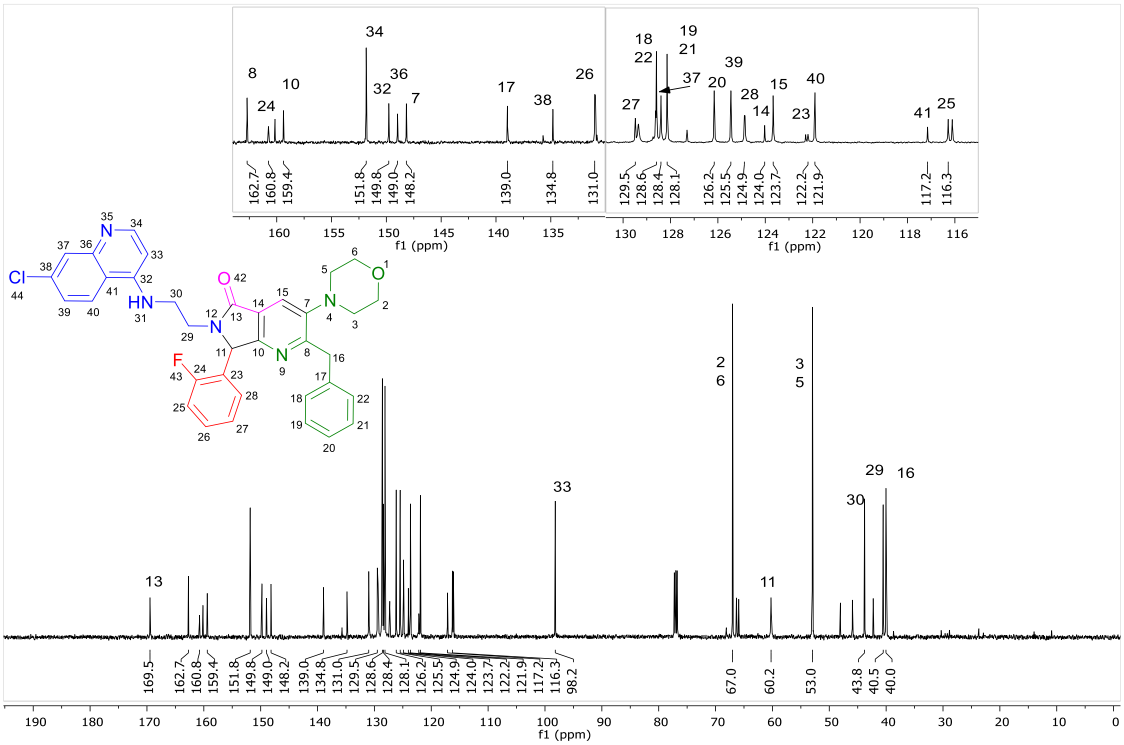

Figure S33: ^{13}C-NMR (125 MHz, $CDCl_3$) spectrum of compound **19e**

Mass Spectrum SmartFormula Report

Acquisition Parameter

Source Type	ESI	Ion Polarity	Positive	Set Nebulizer	0.3 Bar
Focus	Active			Set Dry Heater	180 °C
Scan Begin	50 m/z	Set Capillary	4500 V	Set Dry Gas	4.0 l/min
Scan End	3000 m/z	Set End Plate Offset	-500 V	Set Divert Valve	Waste

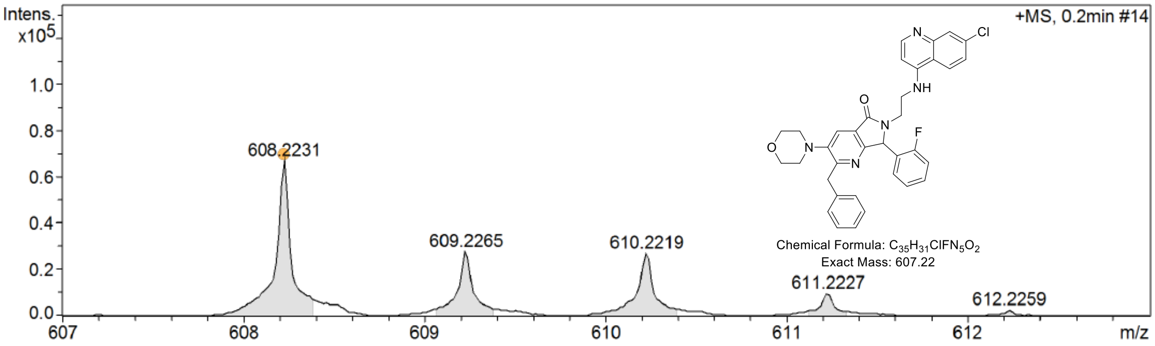

Meas. m/z	#	Ion Formula	Sum Formula	m/z	err [ppm]	mSigma	# Sigma
608.2231	1	C35H32ClFN5O2	C35 H31 Cl F N5 O2	608.2223	-1.3	7.9	2

Figure S34: HRMS (ESI$^+$-TOF) spectrum of compound **19e**

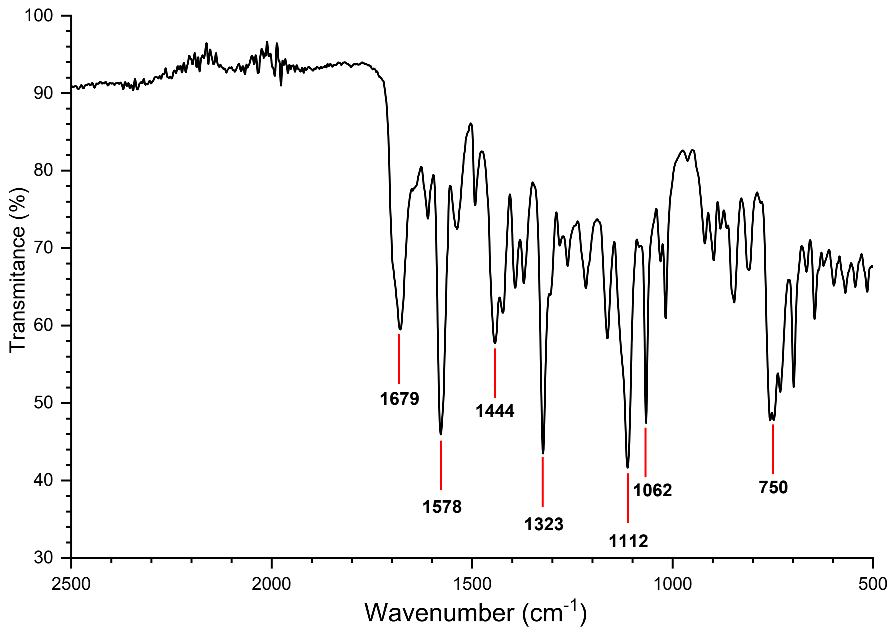

Figure S35: FT-IR (ATR) spectrum of compound **19e**

2-benzyl-6-(3-((7-chloroquinolin-4-yl)amino)propyl)-7-(2-fluorophenyl)-3-morpholino-6,7-dihydro-5*H*-pyrrolo[3,4-*b*]pyridin-5-one **19f**

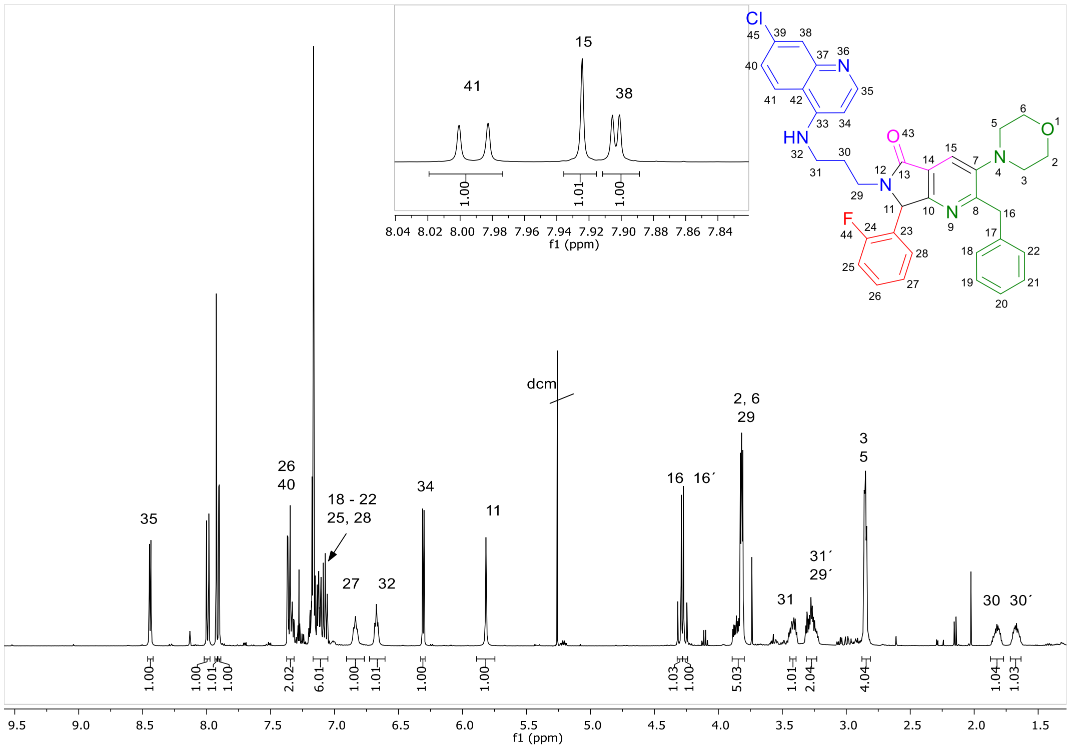

Figure S36: ^{1}H-NMR (500 MHz, $CDCl_3$) spectrum of compound **19f**

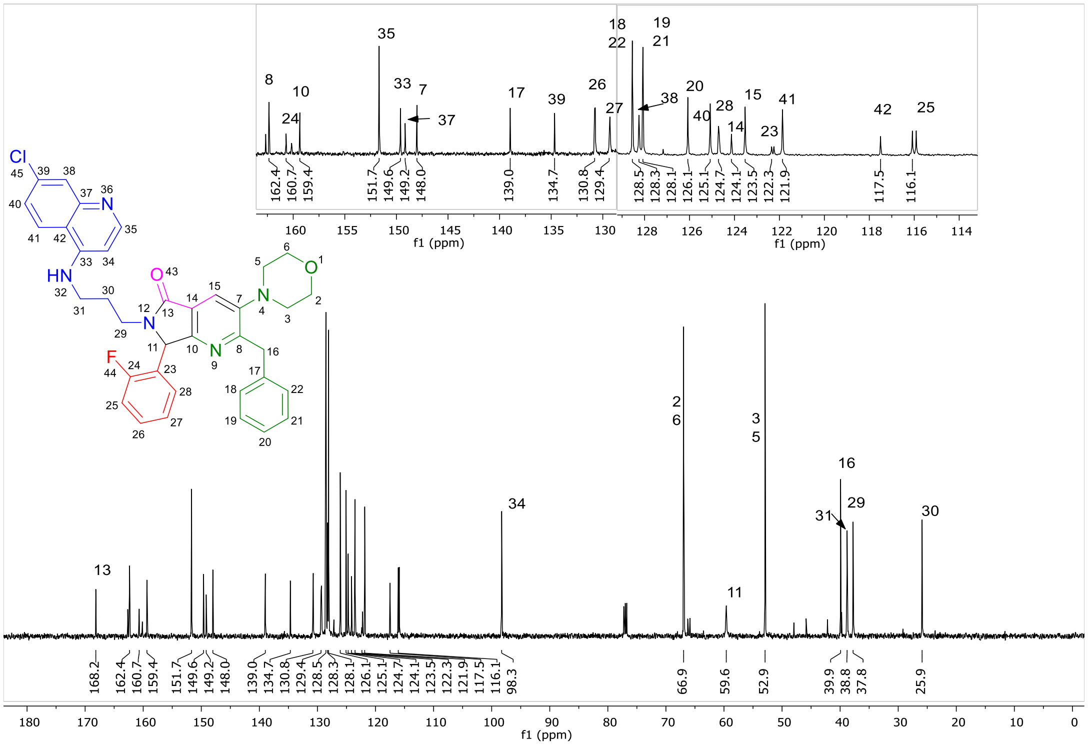

Figure S37: ^{13}C-NMR (125 MHz, $CDCl_3$) spectrum of compound **19f**

Mass Spectrum SmartFormula Report

Acquisition Parameter

Source Type	ESI	Ion Polarity	Positive	Set Nebulizer	0.3 Bar
Focus	Active			Set Dry Heater	180 °C
Scan Begin	50 m/z	Set Capillary	4500 V	Set Dry Gas	4.0 l/min
Scan End	3000 m/z	Set End Plate Offset	-500 V	Set Divert Valve	Waste

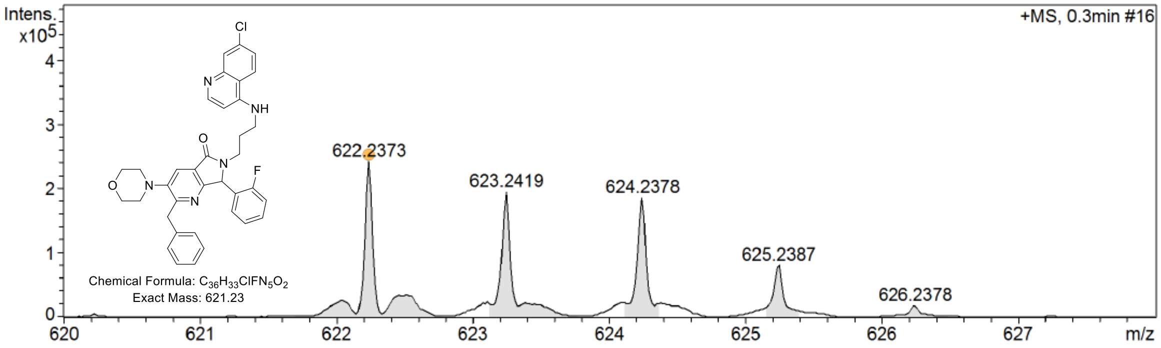

Meas. m/z	#	Ion Formula	Sum Formula	m/z	err [ppm]	mSigma	# Sigma
622.2373	1	C36H34ClFN5O2	C36 H33 Cl F N5 O2	622.2380	-1.1	231.6	2

Figure S38: HRMS (ESI$^+$-TOF) spectrum of compound **19f**

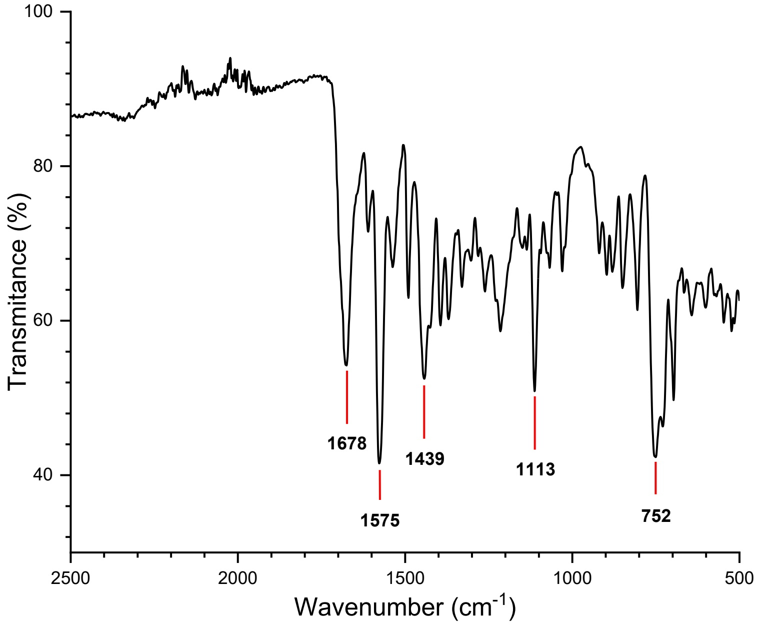

Figure S39: FT-IR (ATR) spectrum of compound **19f**

2-benzyl-6-(2-((7-chloroquinolin-4-yl)amino)ethyl)-3-morpholino-7-(4-(trifluoromethyl)phenyl)-6,7-dihydro-5*H*-pyrrolo[3,4-*b*]pyridin-5-one **19g**

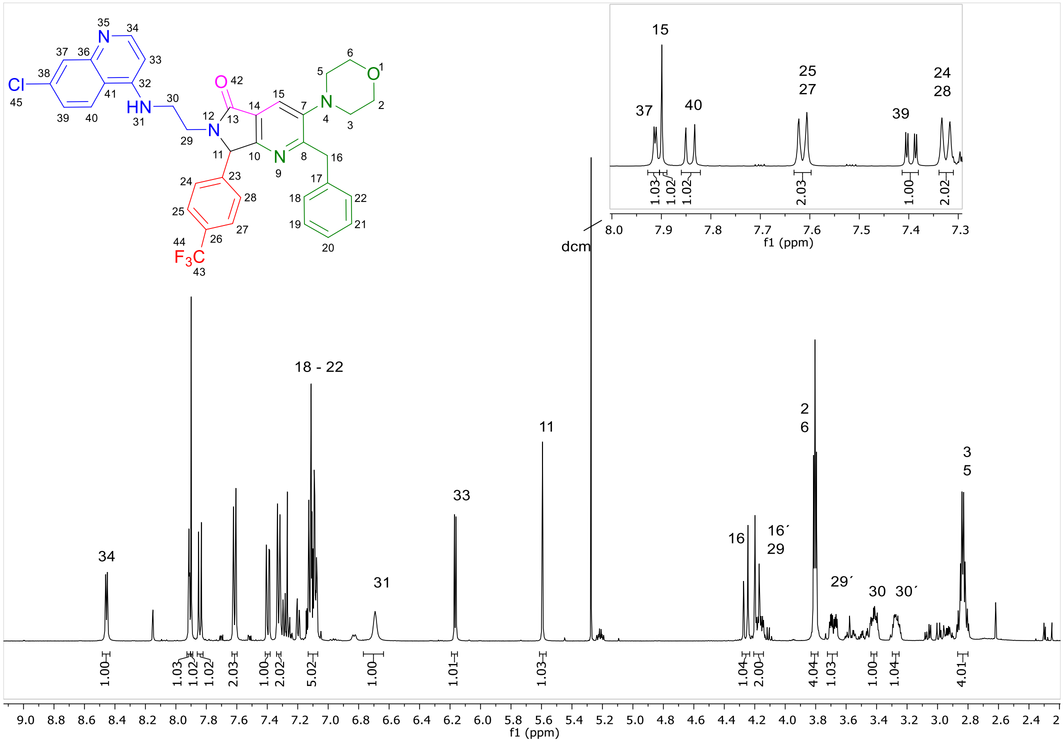

Figure S40: ^{1}H-NMR (500 MHz, $CDCl_3$) spectrum of compound **19g**

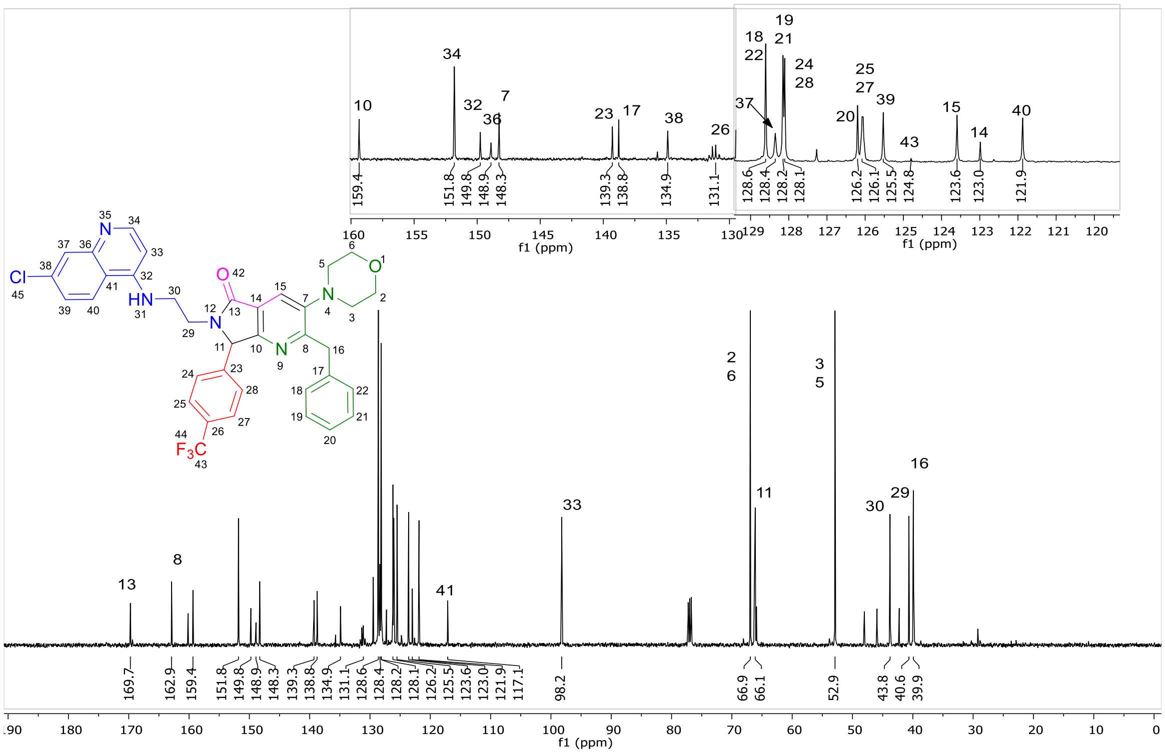

Figure S41: ^{13}C-NMR (125 MHz, $CDCl_3$) spectrum of compound **19g**

Mass Spectrum SmartFormula Report

Acquisition Parameter

Source Type	ESI	Ion Polarity	Positive	Set Nebulizer	0.5 Bar
Focus	Active	Set Capillary	4500 V	Set Dry Heater	150 °C
Scan Begin	50 m/z	Set End Plate Offset	-500 V	Set Dry Gas	4.0 l/min
Scan End	3000 m/z	Set Charging Voltage	0 V	Set Divert Valve	Waste
		Set Corona	0 nA	Set APCI Heater	0 °C

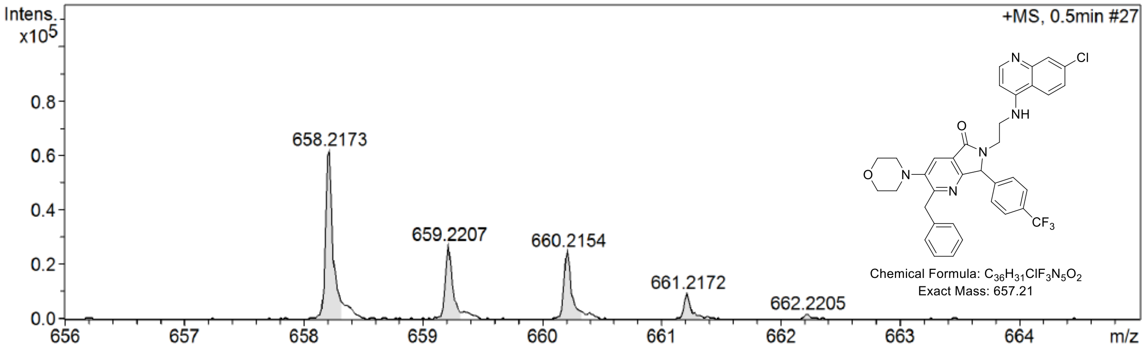

Meas. m/z	#	Ion Formula	Sum Formula	m/z	err [ppm]	mSigma	# Sigma
658.2173	1	C36H32ClF3N5O2	C36 H32 Cl F3 N5 O2	658.2191	2.8	11.7	1

Figure S42: HRMS (ESI$^+$-TOF) spectrum of compound **19g**

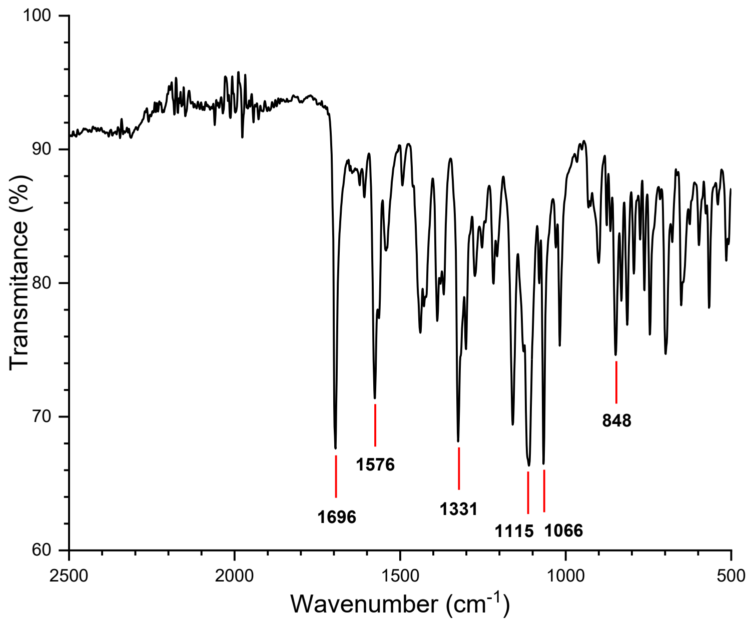

Figure S43: FT-IR (ATR) spectrum of compound **19g**

2-benzyl-6-(2-((7-chloroquinolin-4-yl)amino)ethyl)-3-(piperidin-1-yl)-7-(4-(trifluoromethyl)phenyl)-6,7-dihydro-5*H*-pyrrolo[3,4-*b*]pyridin-5-one **19h**

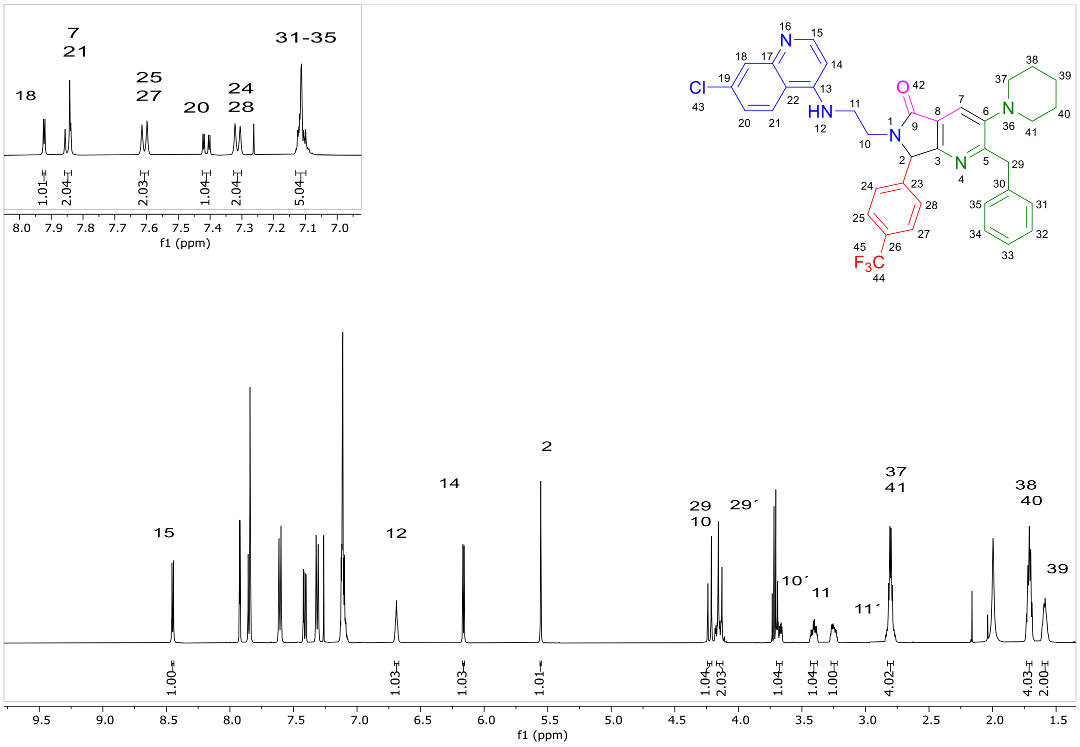

Figure S44: ^{1}H-NMR (500 MHz, $CDCl_3$) spectrum of compound **19h**

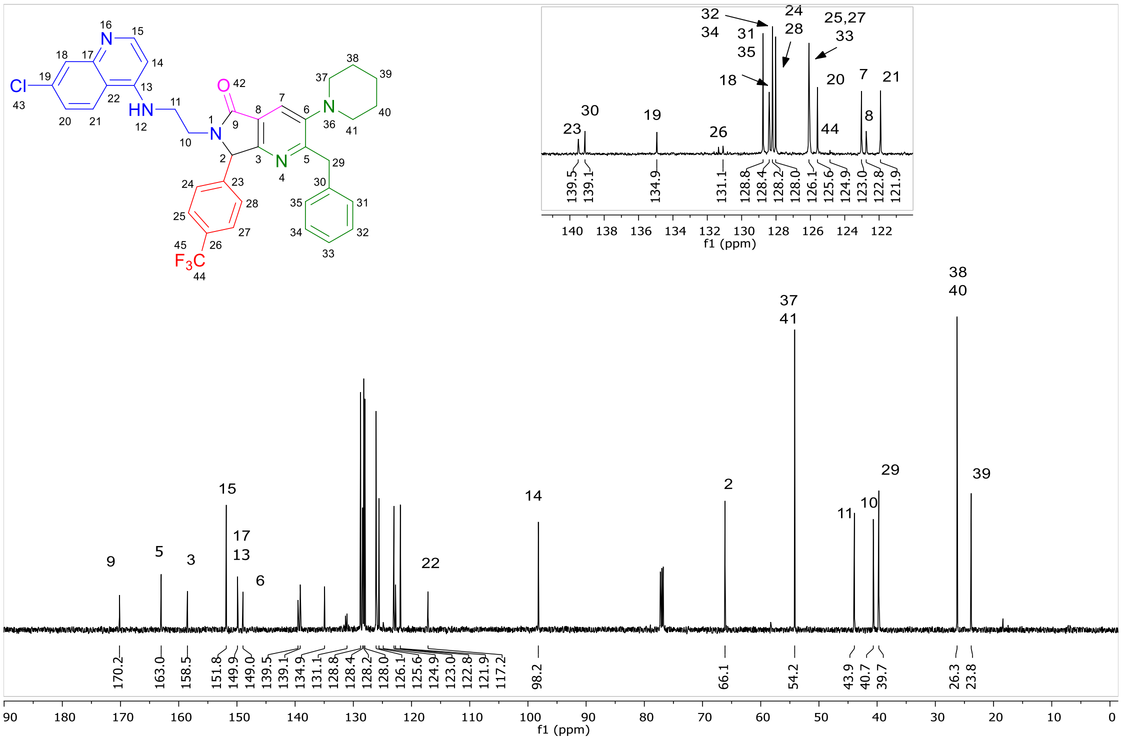

Figure S45: ^{13}C-NMR (125 MHz, $CDCl_3$) spectrum of compound **19h**

Mass Spectrum SmartFormula Report

Acquisition Parameter

Source Type	ESI	Ion Polarity	Positive	Set Nebulizer	0.5 Bar
Focus	Active	Set Capillary	4500 V	Set Dry Heater	150 °C
Scan Begin	50 m/z	Set End Plate Offset	-500 V	Set Dry Gas	4.0 l/min
Scan End	3000 m/z	Set Charging Voltage	0 V	Set Divert Valve	Waste
		Set Corona	0 nA	Set APCI Heater	0 °C

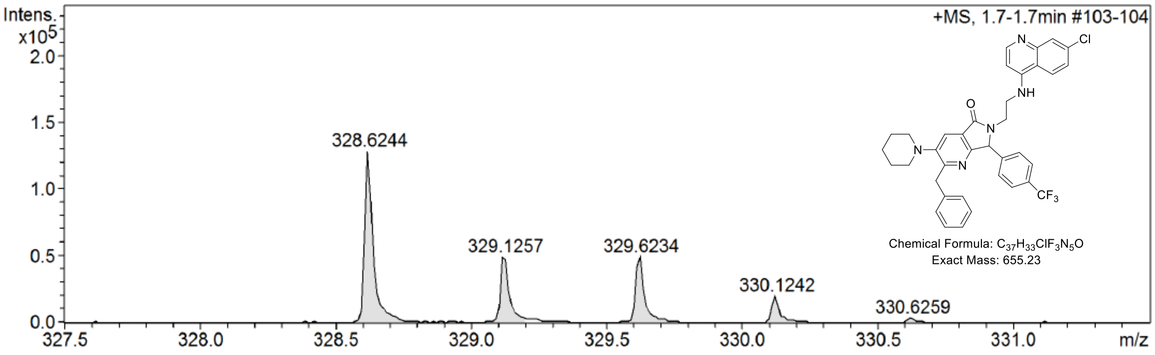

Meas. m/z	#	Ion Formula	Sum Formula	m/z	err [ppm]	mSigma	# Sigma
328.6244	1	C37H35ClF3N5O	C37 H33 Cl F3 N5 O	328.6236	2.7	19.2	2
656.2372	1	C37H34ClF3N5O		656.2398	-4.0	26.9	1

Figure S46: HRMS (ESI$^+$-TOF) spectrum of compound **19h**

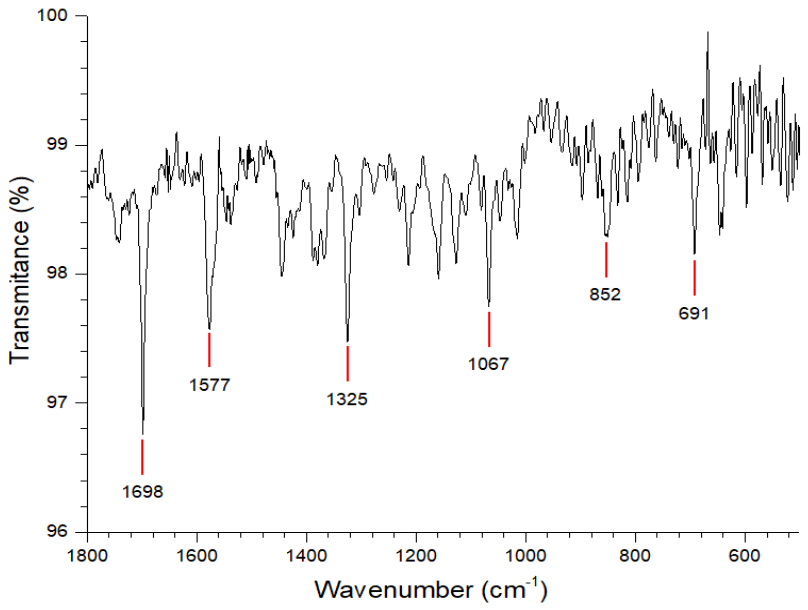

Figure S47: FT-IR (ATR) spectrum of compound **19h**

2-benzyl-6-(2-((7-chloroquinolin-4-yl)amino)ethyl)-3-(diethylamino)-7-(4-(trifluoromethyl)phenyl)-6,7-dihydro-5*H*-pyrrolo[3,4-*b*]pyridin-5-one **19i**

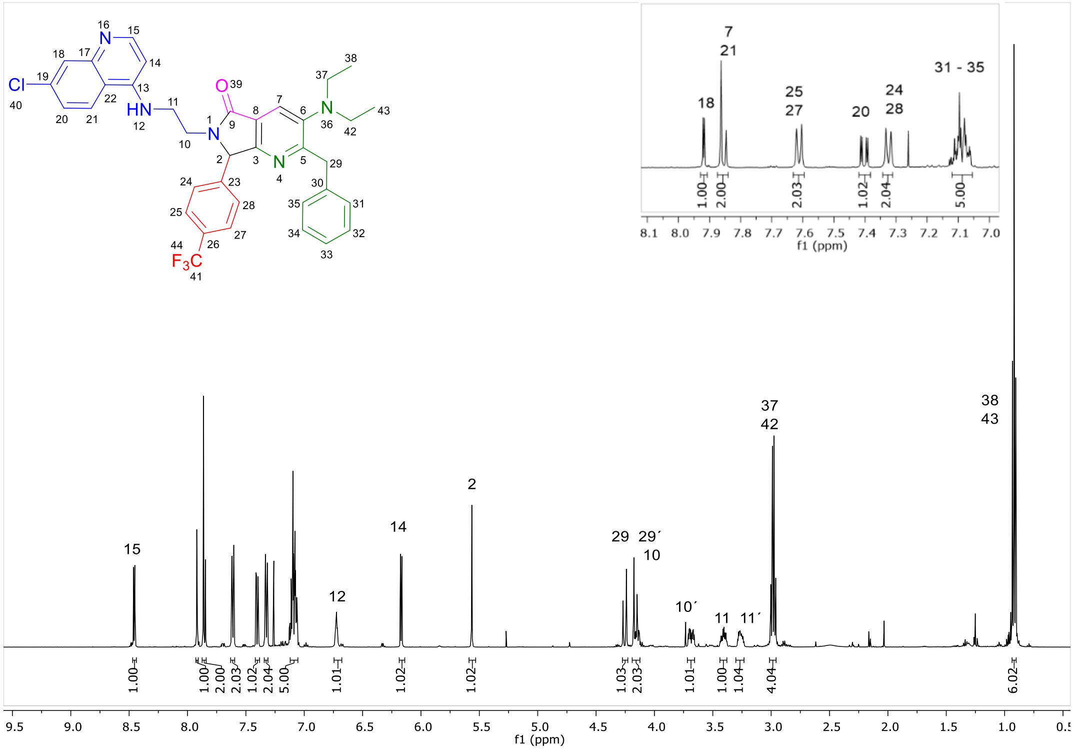

Figure S48: ¹H-NMR (500 MHz, CDCl₃) spectrum of compound **19i**

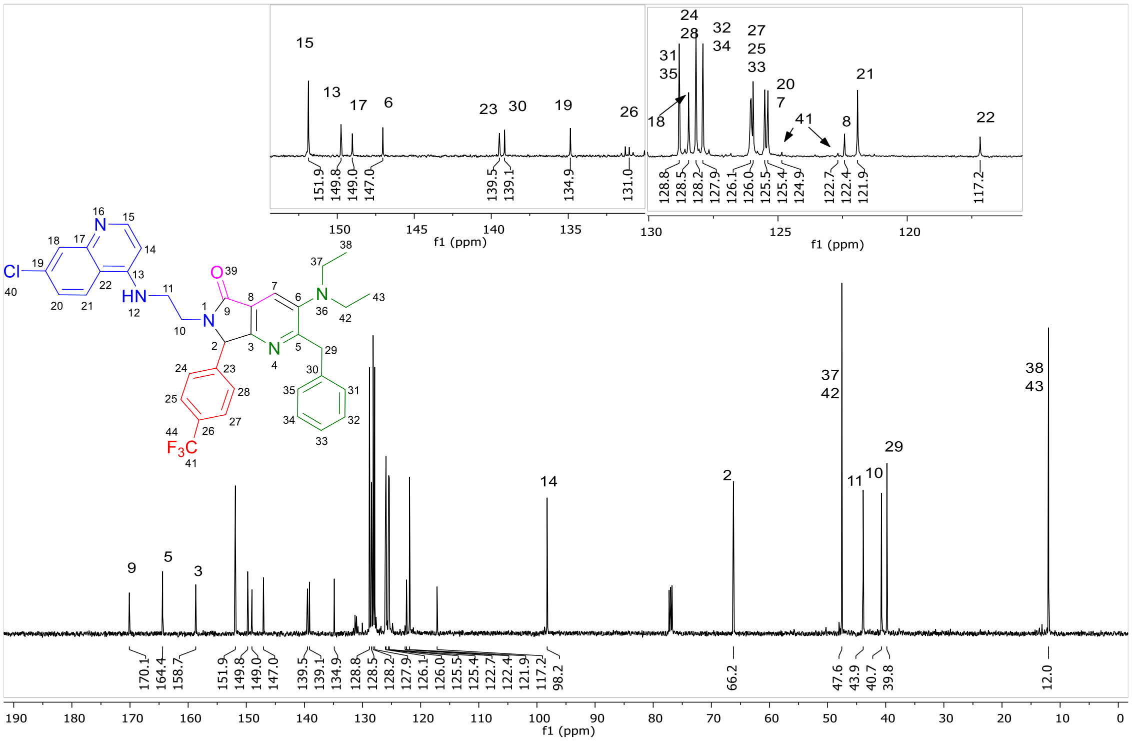

Figure S49: ^{13}C-NMR (125 MHz, $CDCl_3$) spectrum of compound **19i**

Mass Spectrum SmartFormula Report

Acquisition Parameter

Source Type	ESI	Ion Polarity	Positive	Set Nebulizer	0.3 Bar
Focus	Active			Set Dry Heater	180 °C
Scan Begin	50 m/z	Set Capillary	4500 V	Set Dry Gas	4.0 l/min
Scan End	3000 m/z	Set End Plate Offset	-500 V	Set Divert Valve	Waste

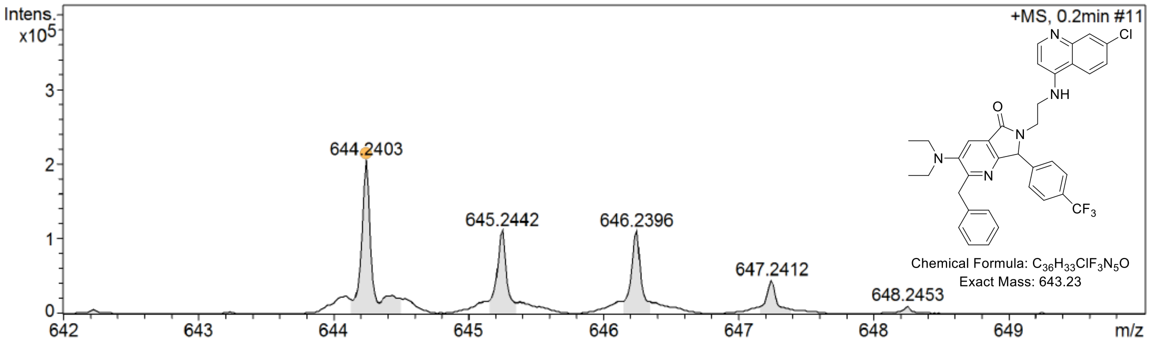

Meas. m/z	#	Ion Formula	Sum Formula	m/z	err [ppm]	mSigma	# Sigma
644.2403	1	C36H34ClF3N5O	C36 H33 Cl F3 N5 O	644.2398	0.6	83.0	3

Figure S50: HRMS (ESI$^+$-TOF) spectrum of compound **19i**

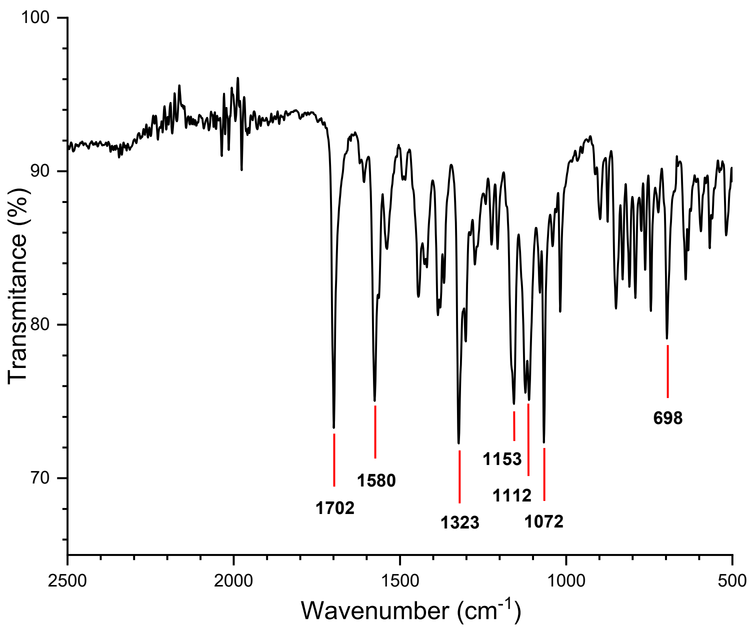

Figure S51: FT-IR (ATR) spectrum of compound **19i**

2-benzyl-7-(3,5-bis(trifluoromethyl)phenyl)-6-(2-((7-chloroquinolin-4-yl)amino)ethyl)-3-morpholino-6,7-dihydro-5*H*-pyrrolo[3,4-*b*]pyridin-5-one **19j**

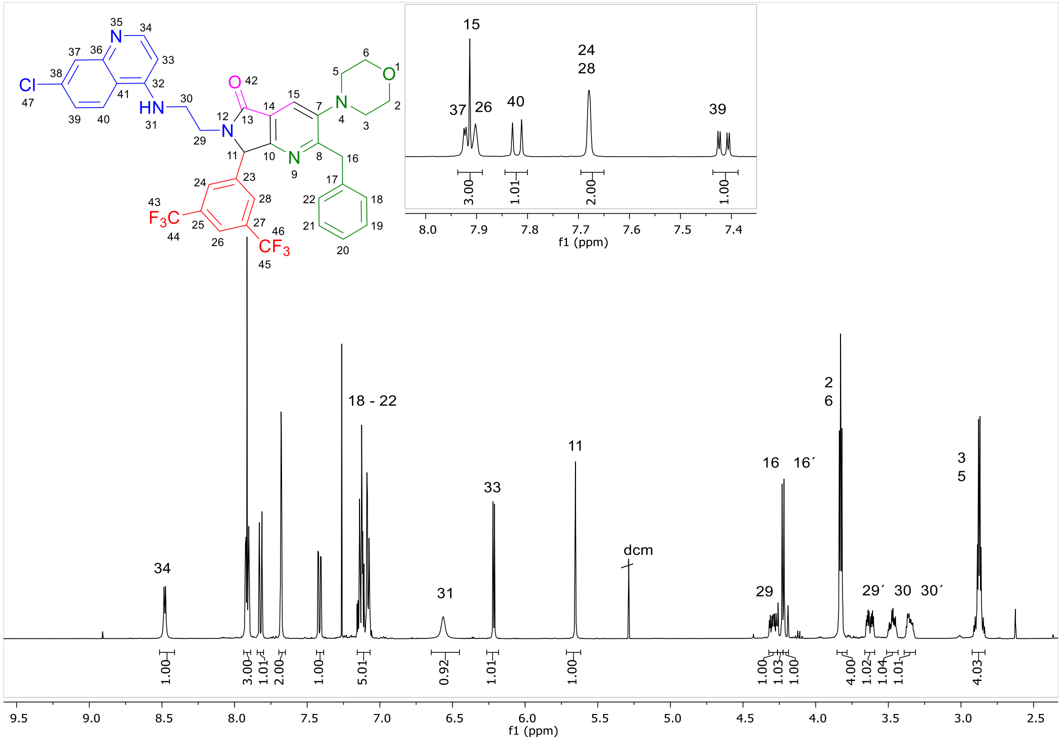

Figure S52: ^{1}H-NMR (500 MHz, $CDCl_3$) spectrum of compound **19j**

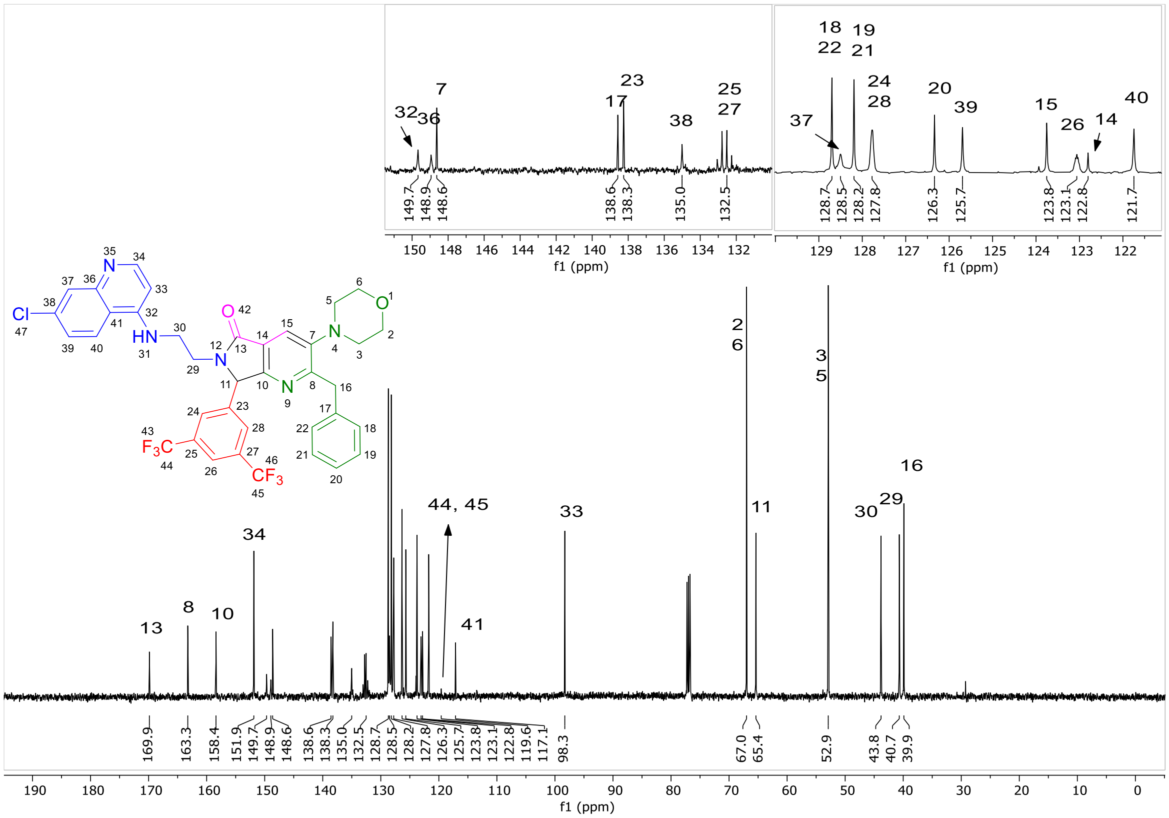

Figure S53: ^{13}C-NMR (125 MHz, $CDCl_3$) spectrum of compound **19j**

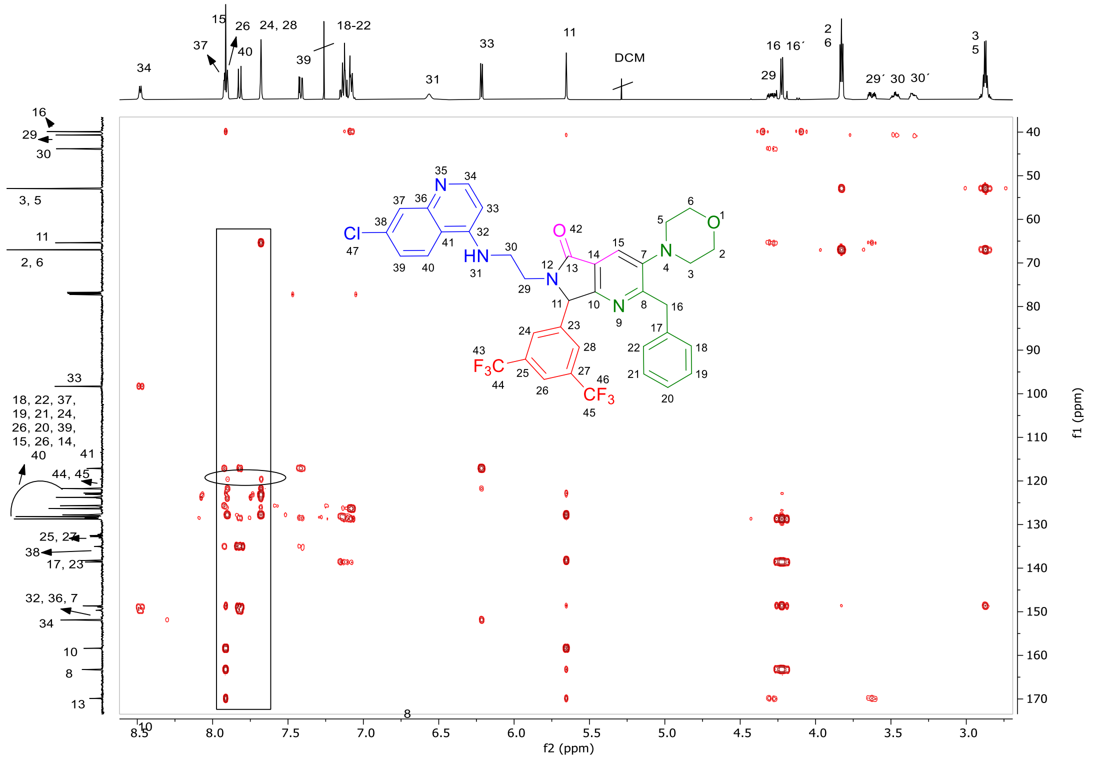

Figure S54: 2D-NMR (HMBC-part I) spectrum of compound **19j**

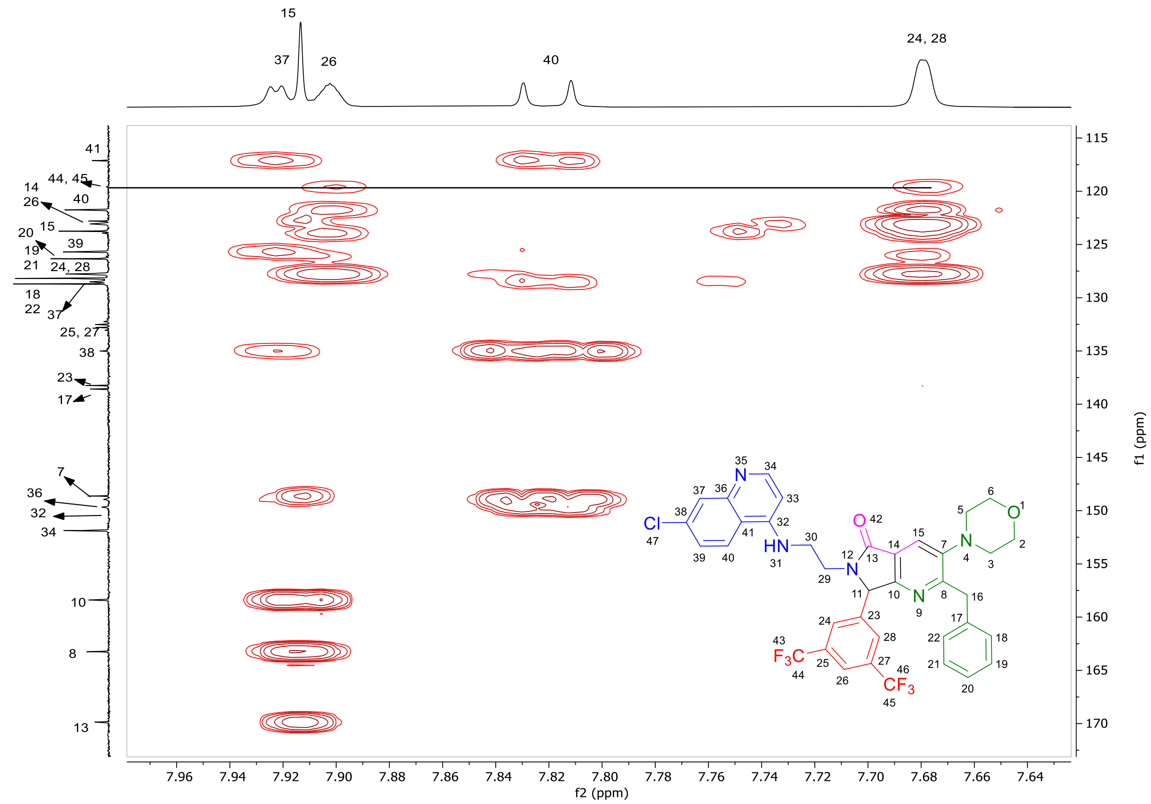

Figure S55: 2D-NMR (HMBC-part II) spectrum of compound **19j**

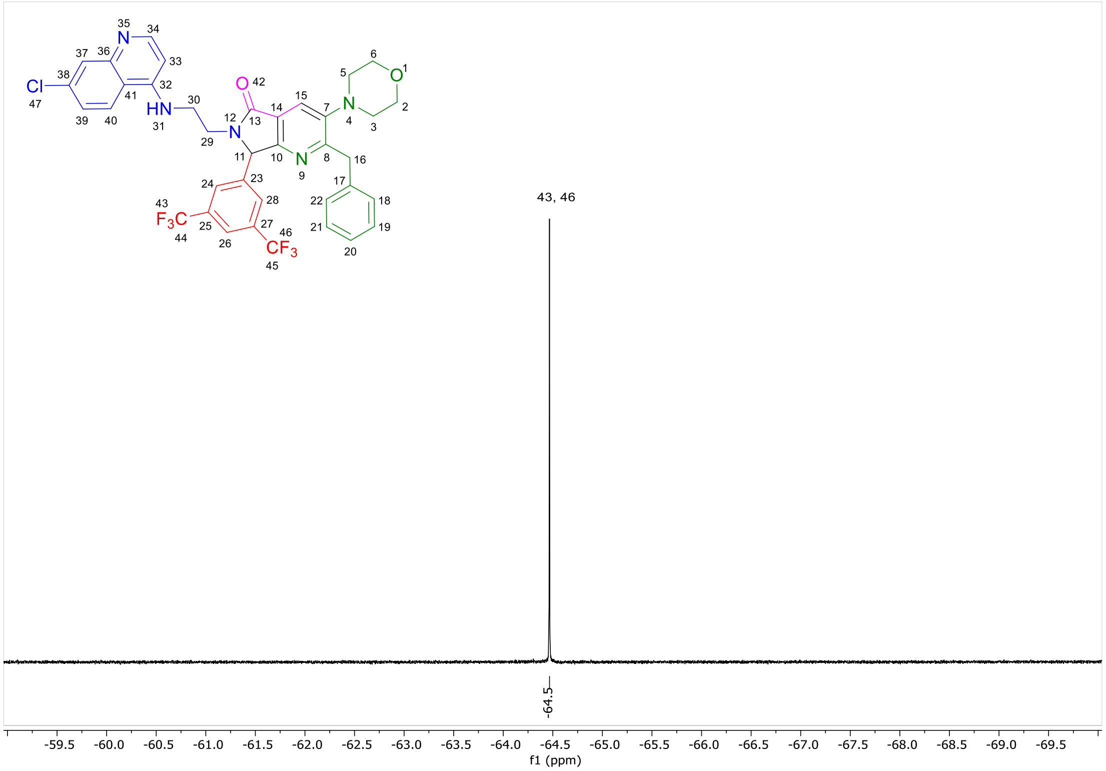

Figure S56: ^{19}F-NMR (500 MHz, $CDCl_3$) spectrum of compound **19j**

Mass Spectrum SmartFormula Report

Acquisition Parameter

Source Type	ESI	Ion Polarity	Positive	Set Nebulizer	0.5 Bar
Focus	Active	Set Capillary	4500 V	Set Dry Heater	150 °C
Scan Begin	50 m/z	Set End Plate Offset	-500 V	Set Dry Gas	4.0 l/min
Scan End	3000 m/z	Set Charging Voltage	0 V	Set Divert Valve	Waste
		Set Corona	0 nA	Set APCI Heater	0 °C

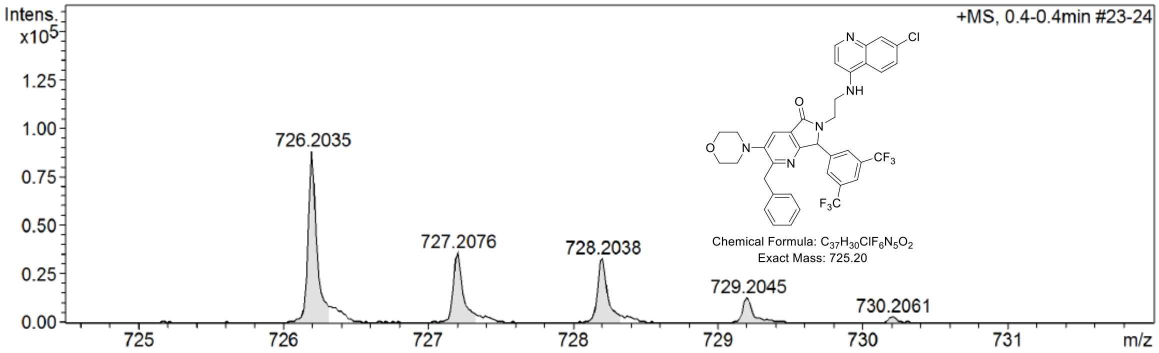

Meas. m/z	#	Ion Formula	Sum Formula	m/z	err [ppm]	mSigma	# Sigma
726.2035	1	C37H31ClF6N5O2	C37 H30 Cl F6 N5 O2	726.2065	-4.1	16.2	1

Figure S57: HRMS (ESI+-TOF) spectrum of compound **19j**

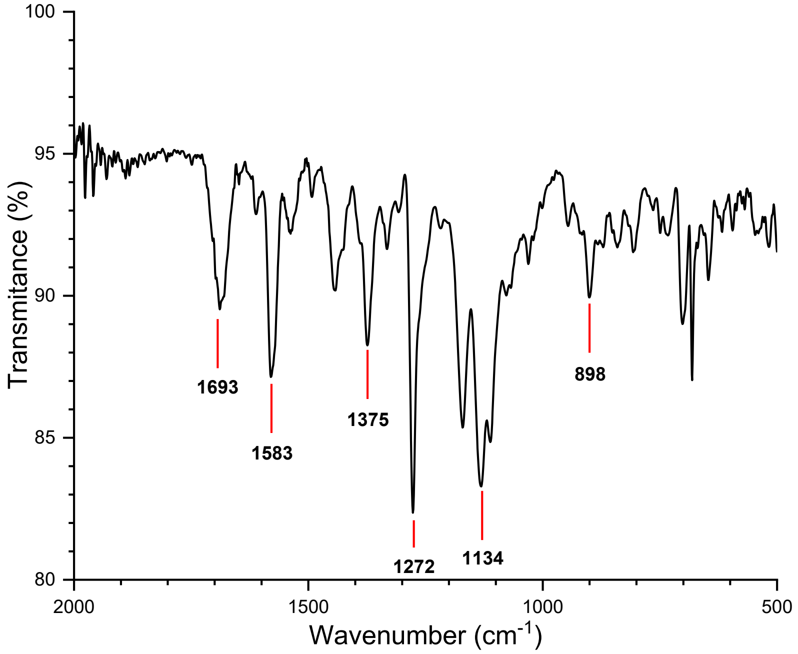

Figure S58: FT-IR (ATR) spectrum of compound **19j**

2-benzyl-7-(3,5-bis(trifluoromethyl)phenyl)-6-(2-((7-chloroquinolin-4-yl)amino)ethyl)-3-(piperidin-1-yl)-6,7-dihydro-5*H*-pyrrolo[3,4-*b*]pyridin-5-one **19k**

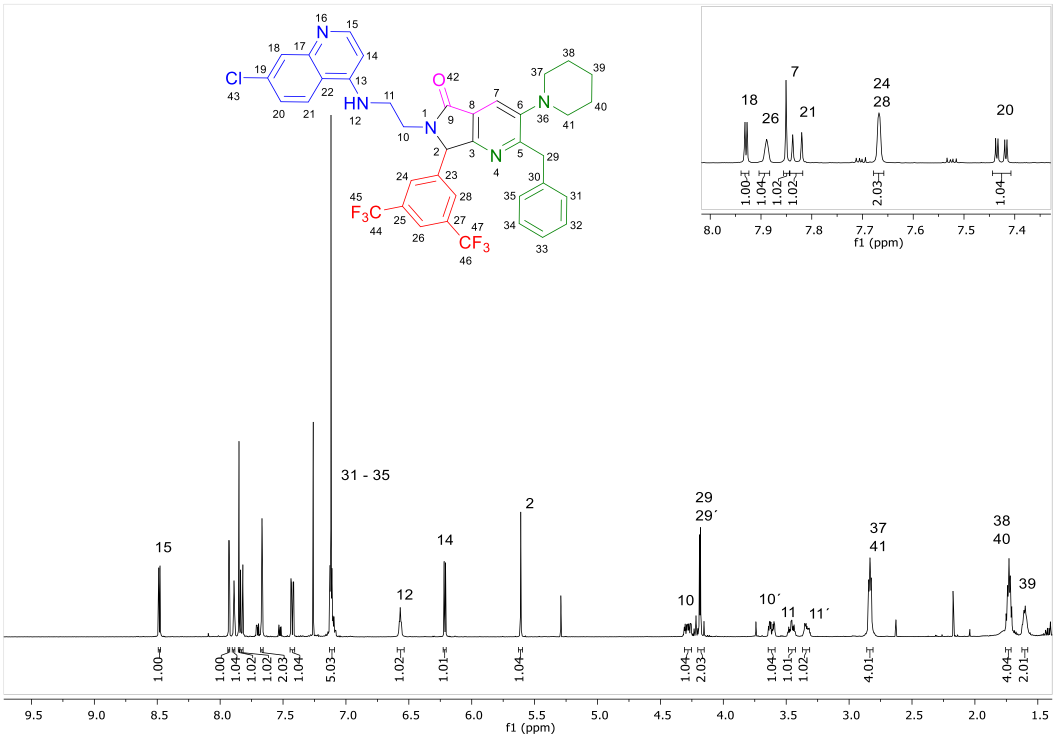

Figure S59: ^{1}H-NMR (500 MHz, $CDCl_3$) spectrum of compound **19k**

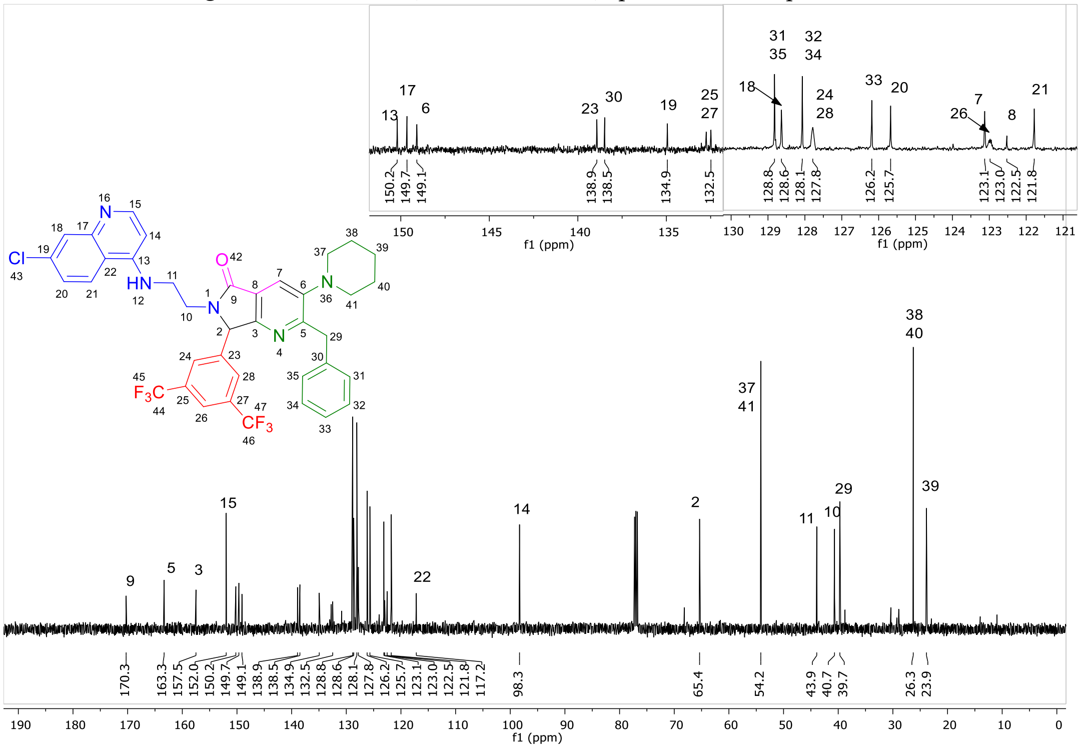

Figure S60: ^{13}C-NMR (125 MHz, $CDCl_3$) spectrum of compound **19k**

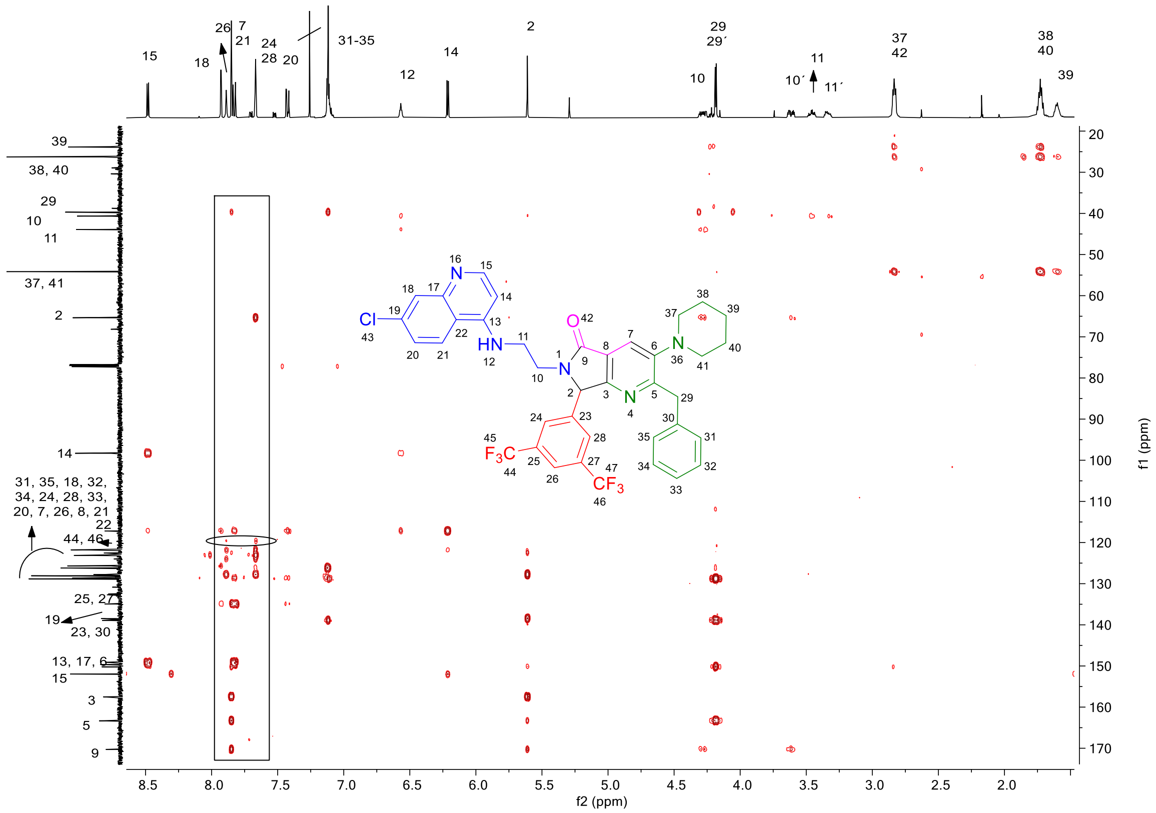

Figure S61: 2D-NMR (HMBC-part I) spectrum of compound **19k**

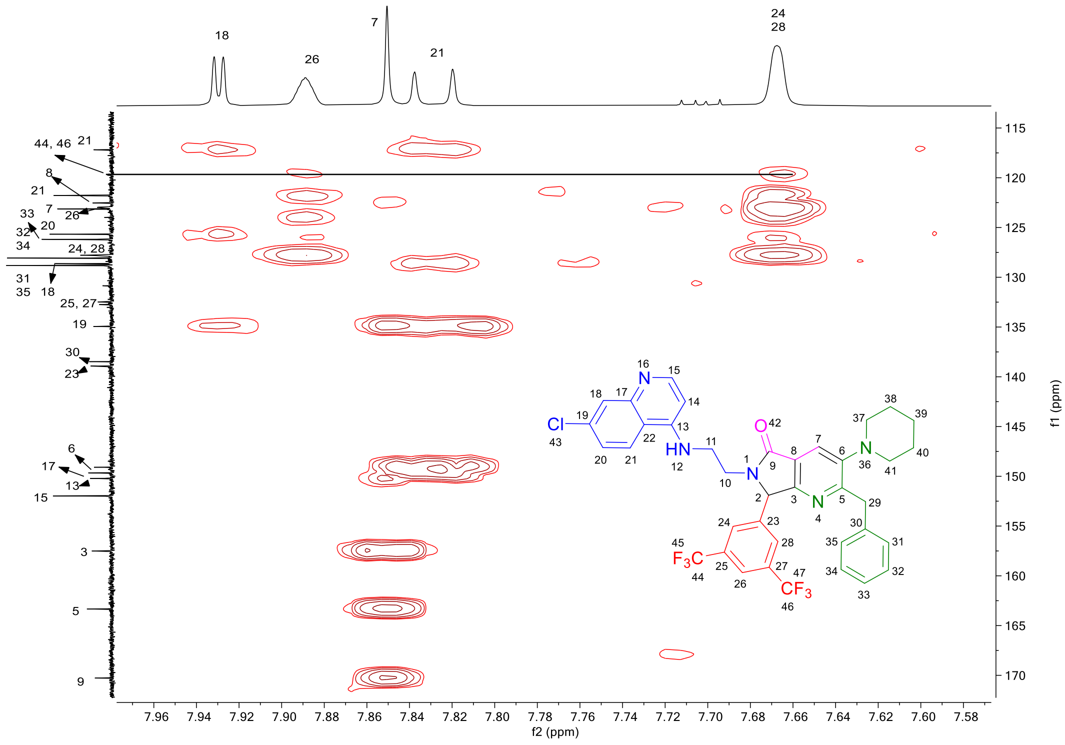

Figure S62: 2D-NMR (HMBC-part II) spectrum of compound **19k**

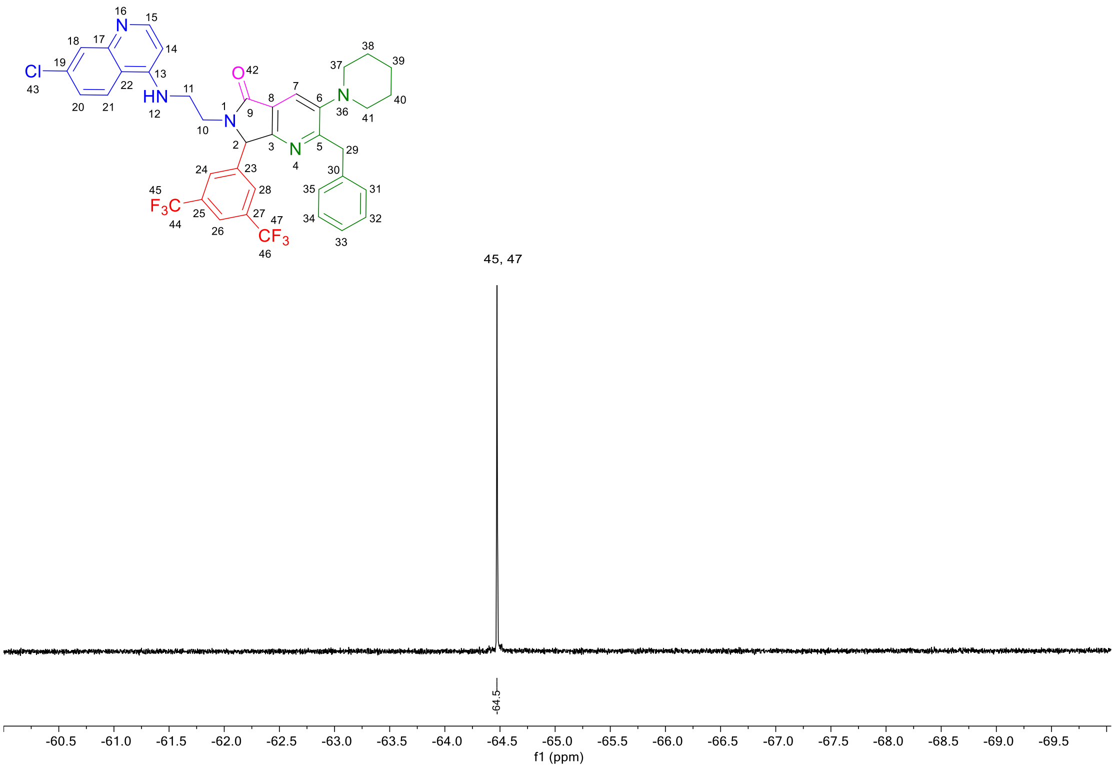

Figure S63: ^{19}F-NMR (500 MHz, $CDCl_3$) spectrum of compound **19k**

Mass Spectrum SmartFormula Report

Acquisition Parameter

Source Type	ESI	Ion Polarity	Positive	Set Nebulizer	0.3 Bar
Focus	Active			Set Dry Heater	180 °C
Scan Begin	50 m/z	Set Capillary	4500 V	Set Dry Gas	4.0 l/min
Scan End	3000 m/z	Set End Plate Offset	-500 V	Set Divert Valve	Waste

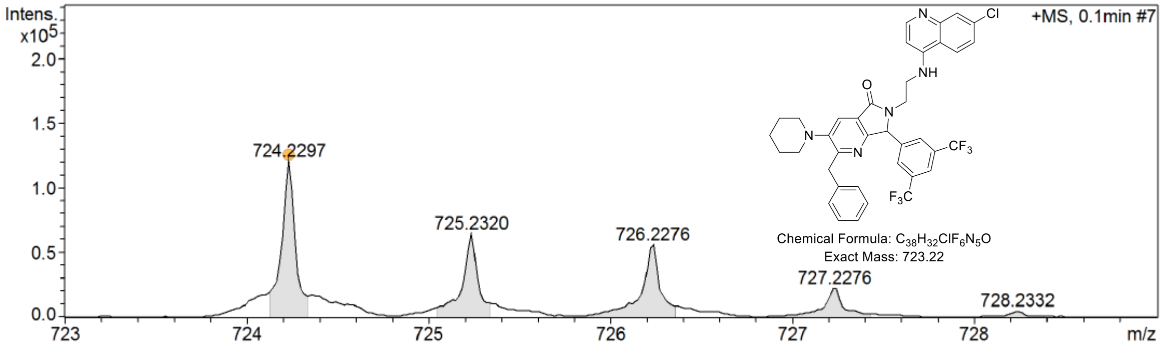

Meas. m/z	#	Ion Formula	Sum Formula	m/z	err [ppm]	mSigma	# Sigma
724.2297	1	C38H33ClF6N5O	C38 H32 Cl F6 N5 O	724.2272	3.4	48.4	3

Figure S64: HRMS (ESI$^+$-TOF) spectrum of compound **19k**

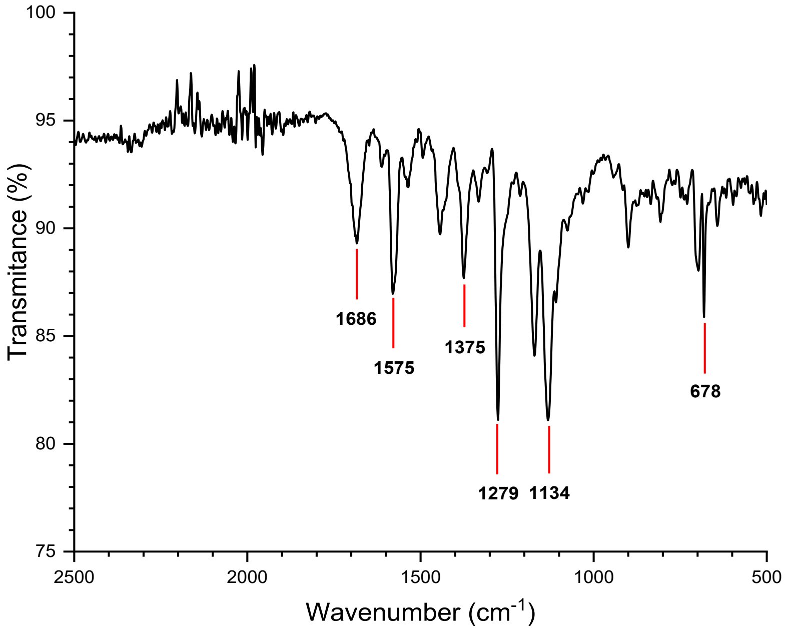

Figure S65: FT-IR (ATR) spectrum of compound **19k**

2-benzyl-6-(3-((7-chloroquinolin-4-yl)amino)propyl)-3-morpholino-7-(perfluorophenyl)-6,7-dihydro-5*H*-pyrrolo[3,4-*b*]pyridin-5-one **19l**

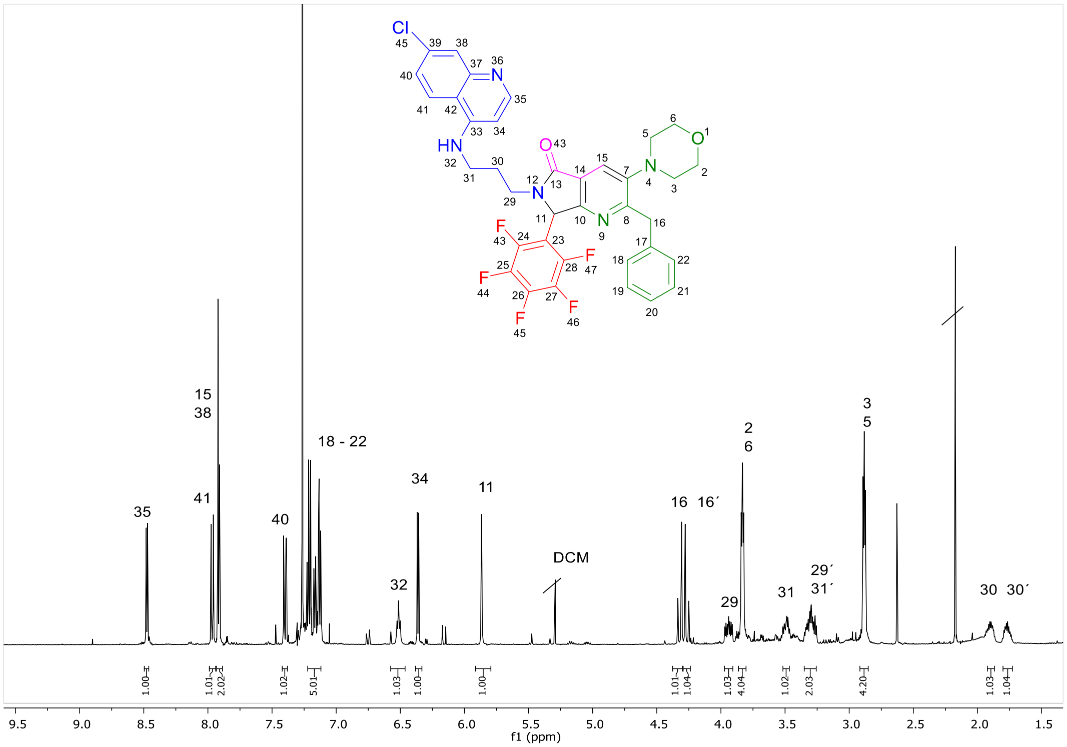

Figure S66: ¹H-NMR (500 MHz, $CDCl_3$) spectrum of compound **19l**

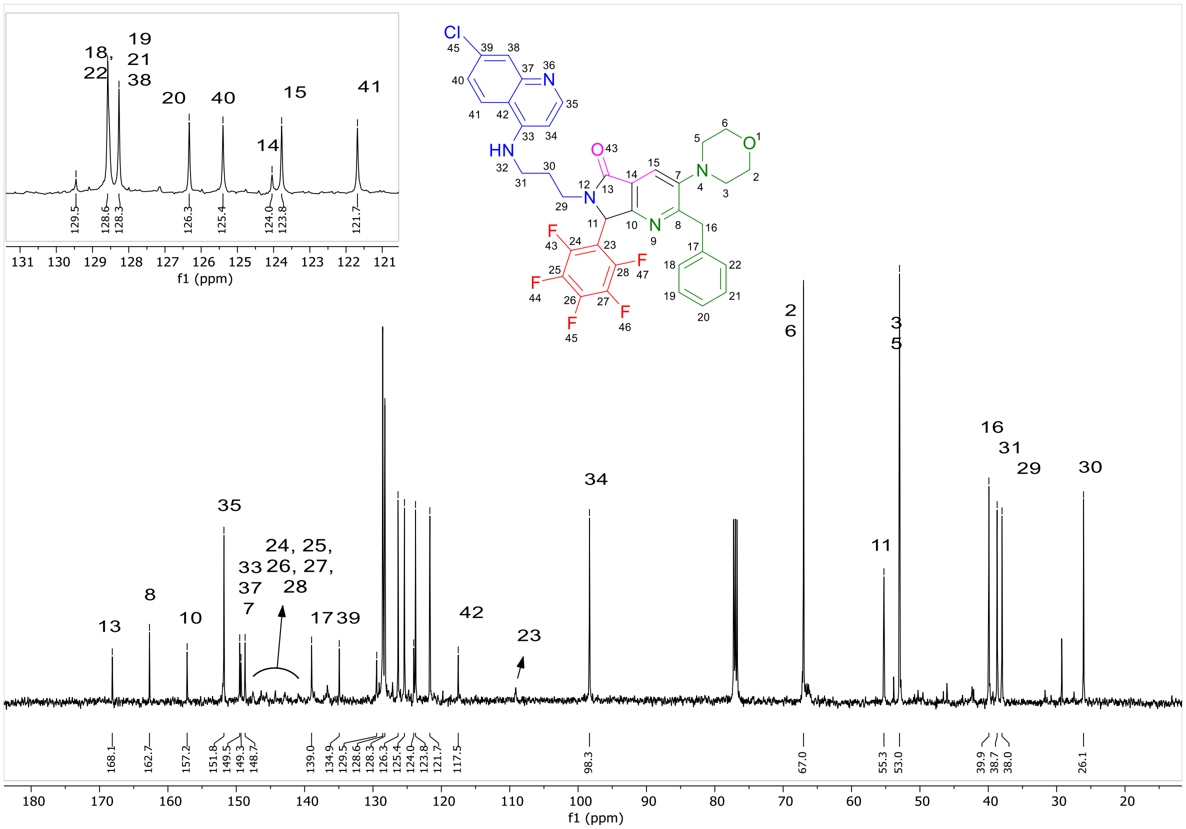

Figure S67: ^{13}C-NMR (125 MHz, $CDCl_3$) spectrum of compound **19l**

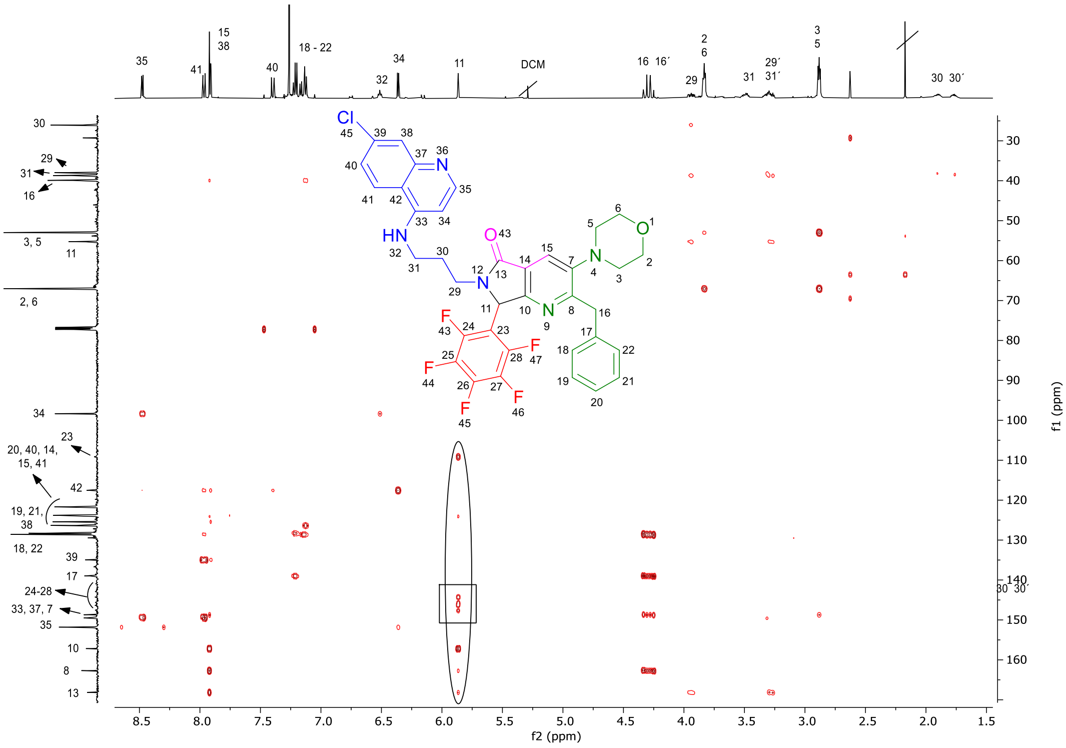

Figure S68: 2D-NMR (HMBC-part I) spectrum of compound **19l**

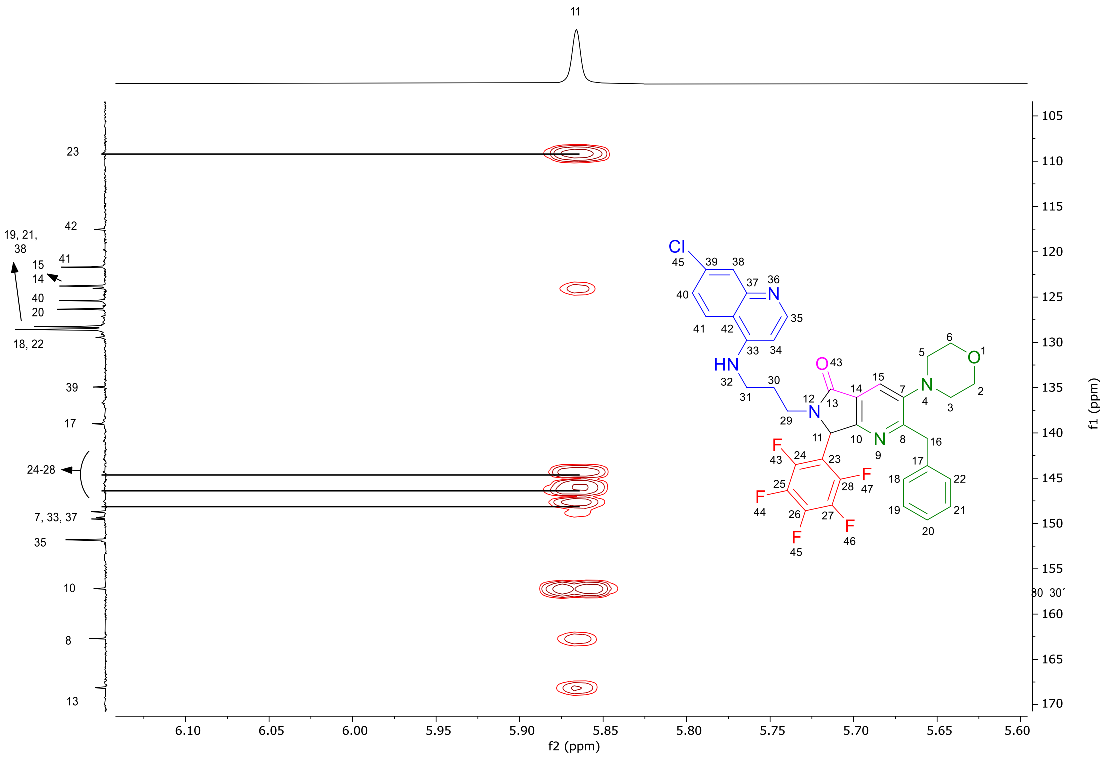

Figure S69: 2D-NMR (HMBC-part II) spectrum of compound **19l**

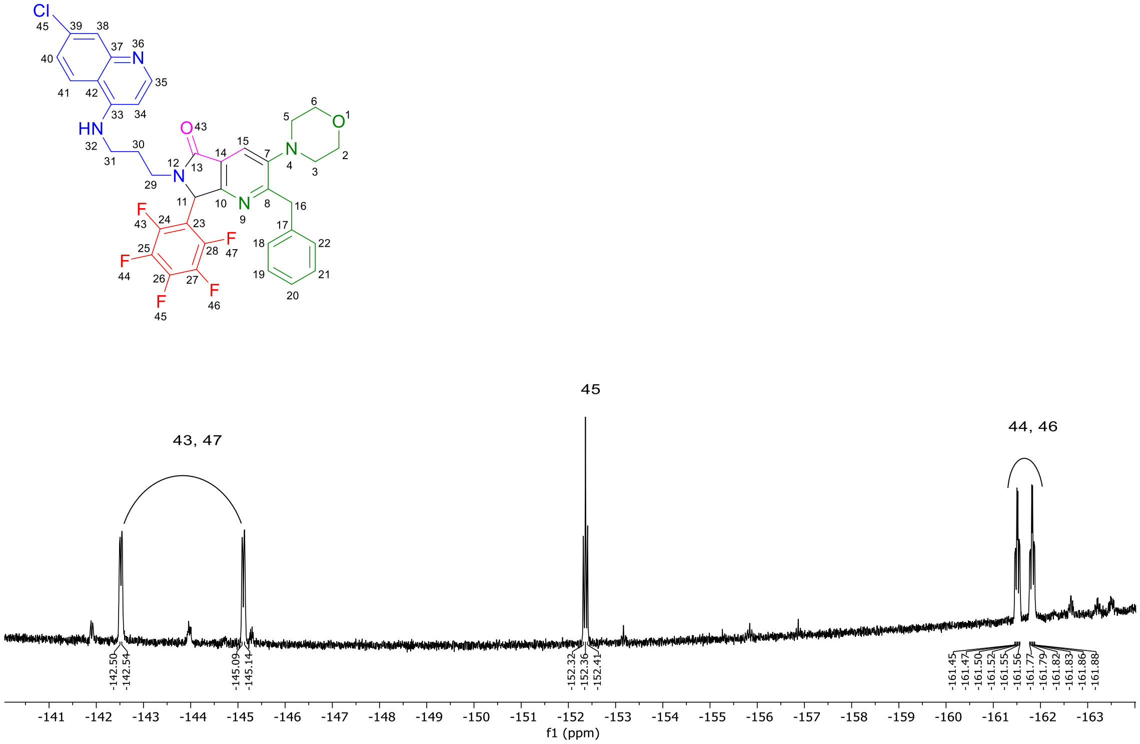

Figure S70: ^{19}F-NMR (500 MHz, $CDCl_3$) spectrum of compound **19l**

Mass Spectrum SmartFormula Report

Acquisition Parameter

Source Type	ESI	Ion Polarity	Positive	Set Nebulizer	0.5 Bar
Focus	Active	Set Capillary	4500 V	Set Dry Heater	150 °C
Scan Begin	50 m/z	Set End Plate Offset	-500 V	Set Dry Gas	4.0 l/min
Scan End	3000 m/z	Set Charging Voltage	0 V	Set Divert Valve	Waste
		Set Corona	0 nA	Set APCI Heater	0 °C

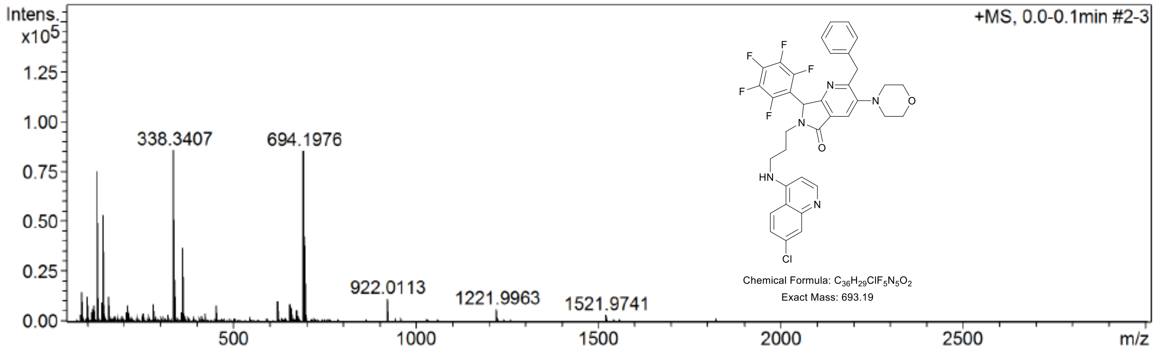

Meas. m/z	#	Ion Formula	Sum Formula	m/z	err [ppm]	mSigma	# Sigma
694.1976	1	C36H30ClF5N5O2	C36 H29 Cl F5 N5 O2	694.2003	-3.8	16.5	2

Figure S71: HRMS (ESI$^+$-TOF) spectrum of compound **19l**

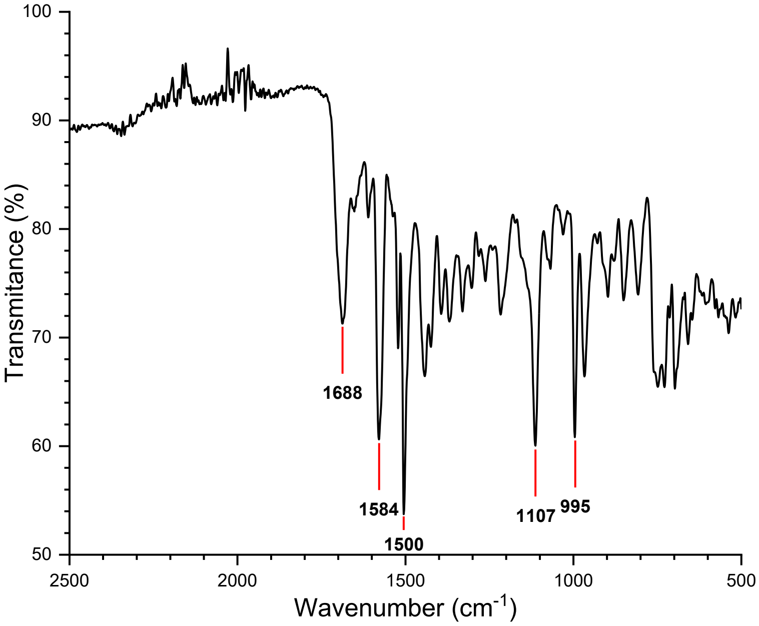

Figure S72: FT-IR (ATR) spectrum of compound **19l**

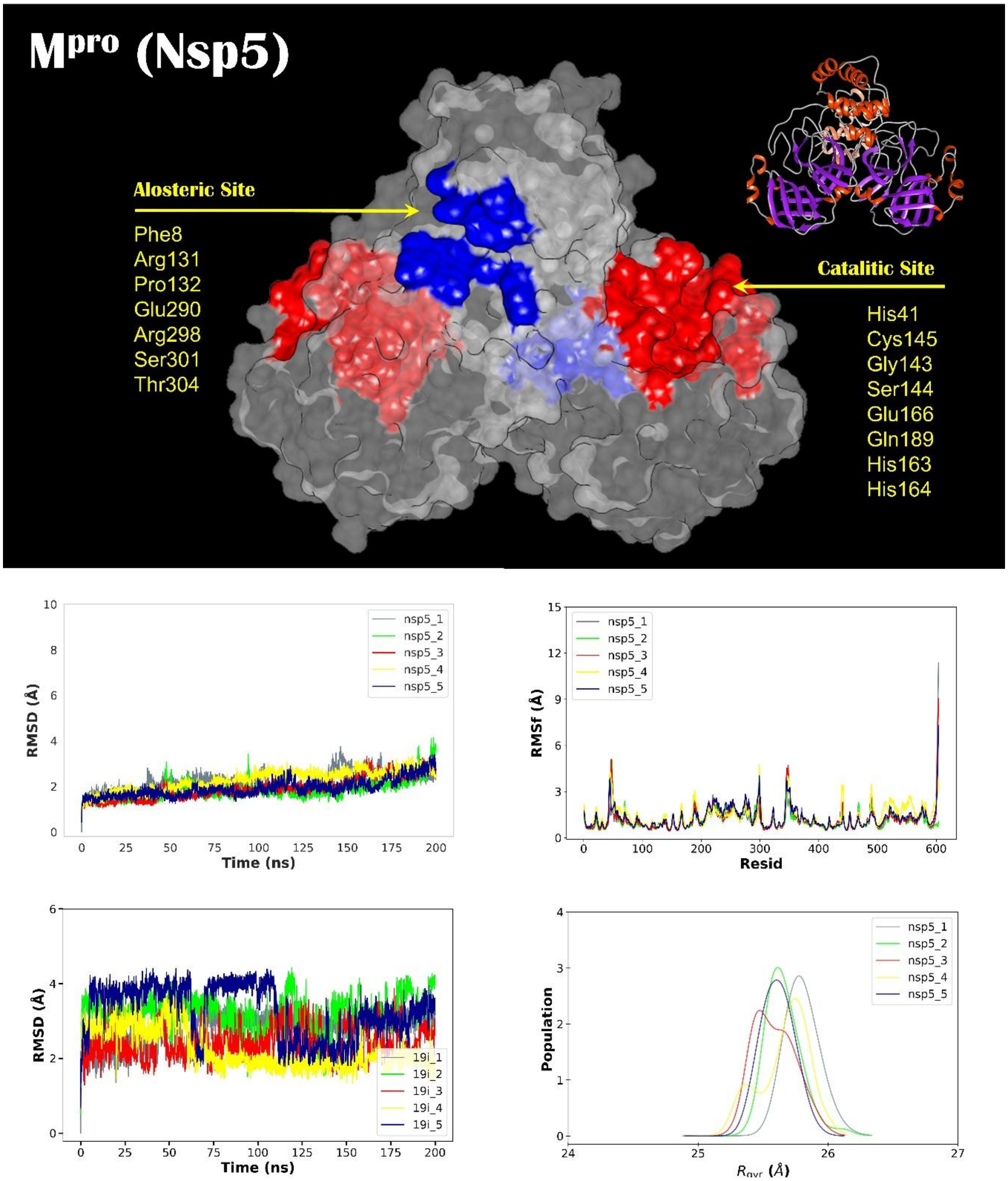

Figure S73: Structural Representation of the Main Protease of SARS-CoV-2 (M_{pro}/nsp5). The catalytic site (in red) and an allosteric site (in blue) are emphasized on the protein's surface. The results of 200-ns molecular dynamics simulations were shown in the lower panel. The RMSD versus time graph shows how stable each replica is overall, the RMSF versus residue graph shows how flexible each residue is, and the radius of gyration distribution shows how compact the protein is on average.

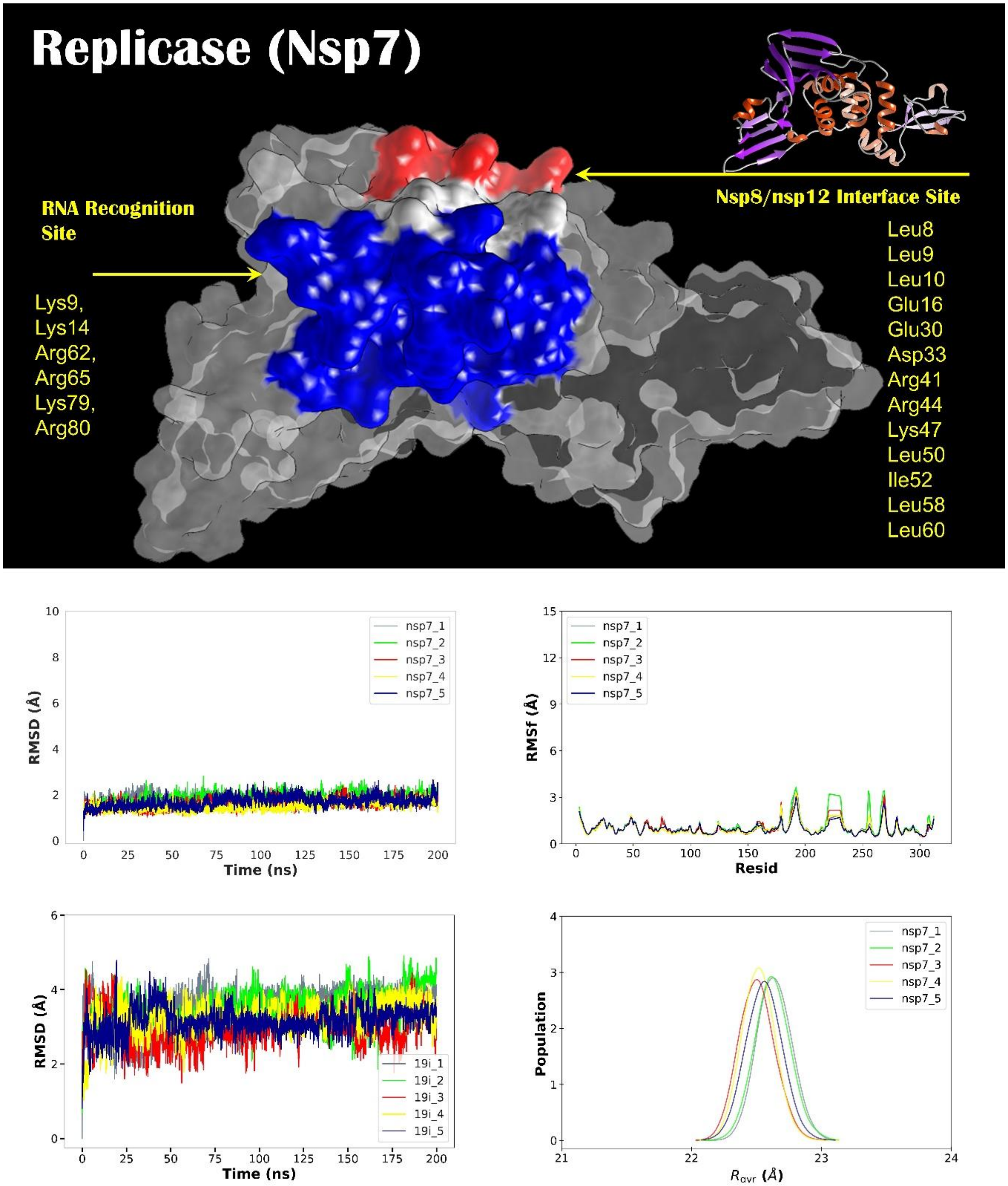

Figure S74: Structural Depiction of the SARS-CoV-2 Replicase (nsp7). The three-dimensional representation of the protein highlights the RNA recognition site (blue) and the nsp8/nsp12 interface (red). The results of the 200-ns molecular dynamics simulations (five replicas) are shown in the lower section. The RMSD over time graph shows how stable each replica is overall, the RMSF per residue graph shows how flexible each replica is locally, and the radius of gyration distribution graph shows how compact the protein is on average. The results show which parts are the most mobile and how they might be related to catalytic function. They also show how nsp7 interacts with other parts of the replicase–transcriptase complex.

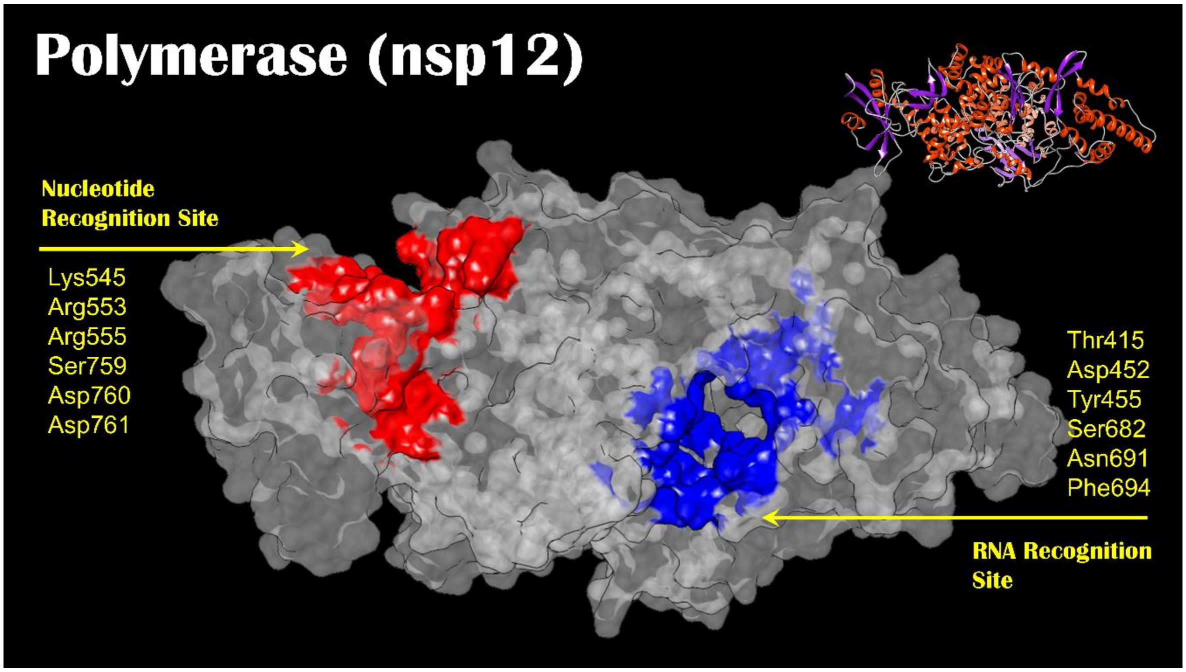

Figure S75: Structural Representation of the SARS-CoV-2 Polymerase (nsp12). The surface model shows the nucleotide recognition site (in red) and the RNA recognition site (in blue). It also shows important residues on the surface, such as LYS545, ARG553, SER759, THR415, ASP452, and more.

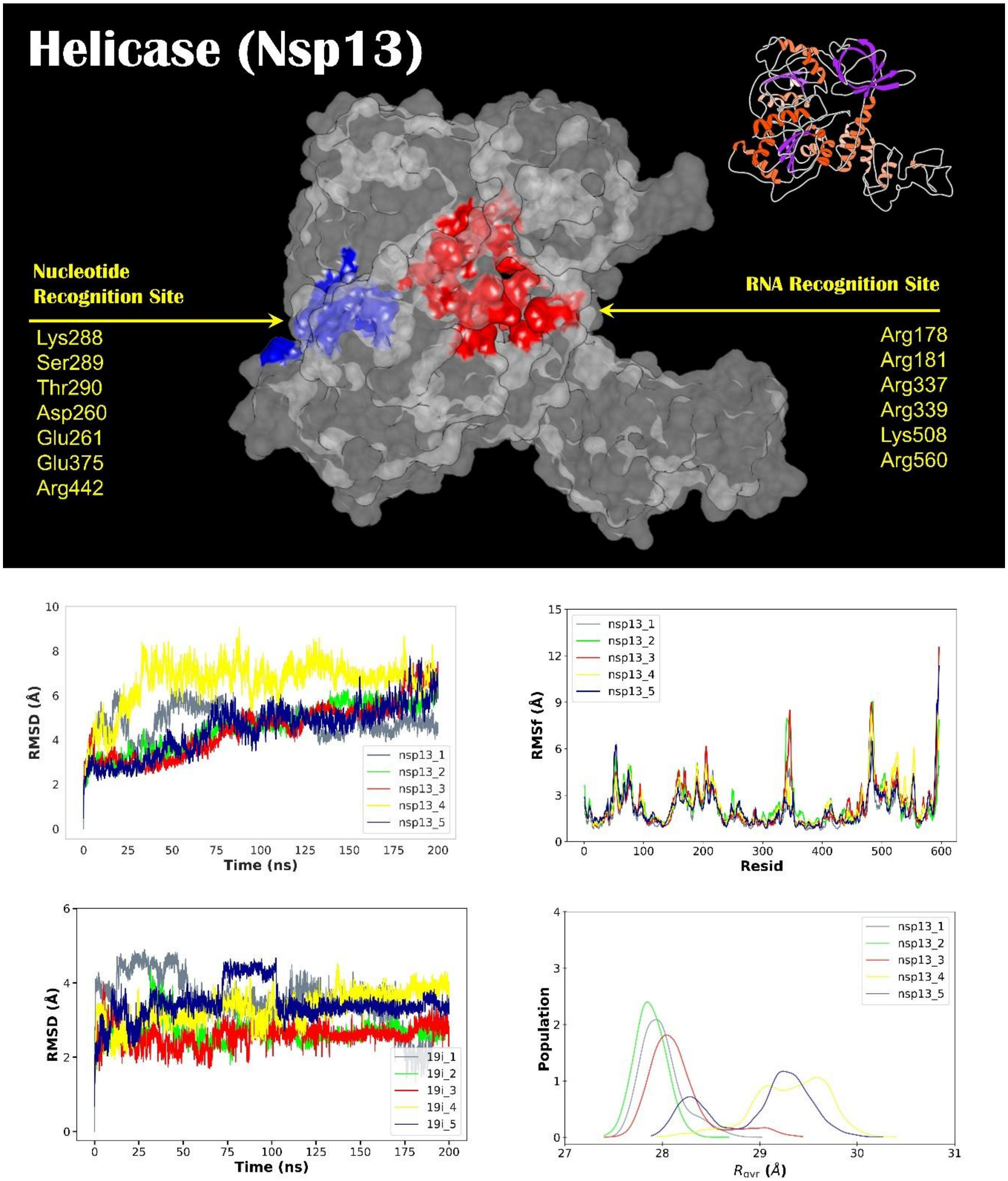

Figure S76: Structural Depiction of the SARS-CoV-2 Helicase (nsp13). The surface of the protein shows the nucleotide recognition site (in blue) and the RNA recognition site (in red). Important residues like LYS288, SER289, THR290, ARG178, ARG181, ARG337, ARG339, LYS508, and ARG560 are marked in the right places. The lower graphs show the results of 200-ns molecular dynamics simulations with five copies: the RMSD over time (left graph) shows how stable each copy is overall; the RMSF per residue (center graph) shows how flexible each copy is locally; and the radius of gyration distribution (right graph) shows how compact the helicase is on average. This enzyme is crucial for unwinding viral RNA, rendering it a pertinent target for reducing SARS-CoV-2 replication.

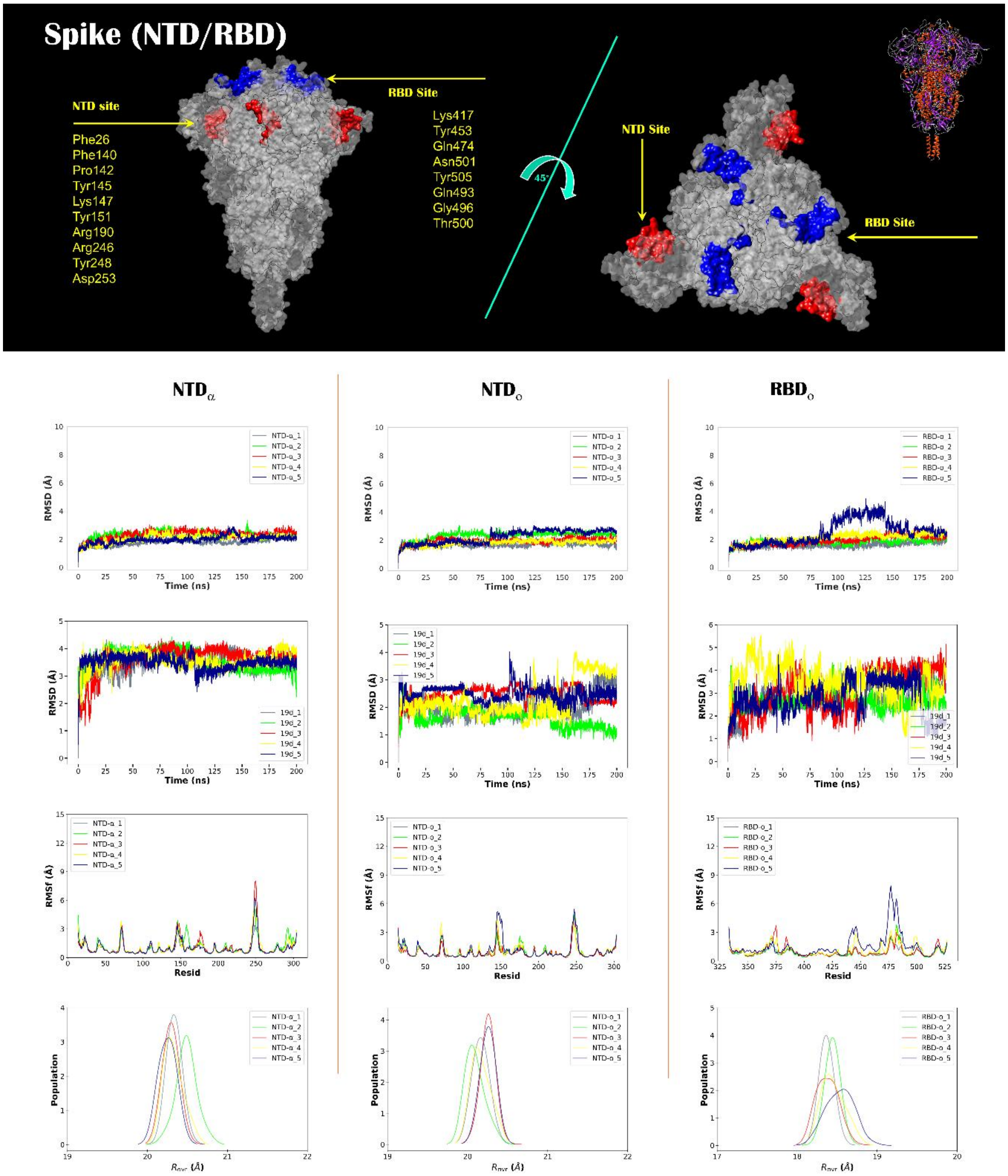

Figure S77: Illustrates the structure representation of the spike protein using its *N*-terminal domain and the receptor-binding domain of the SARS-CoV-2 virus. The top part shows the cell recognition sites (NTD) and the ACE2 binding site (RBD), showing how they are different in the Alpha and Omicron versions. The lower panels show the results of five 200-ns molecular dynamics simulations for each domain (NTD±, NTDo, and RBDo): the RMSD versus time graph (top) shows how stable the protein is overall, the RMSF versus residue graph (middle) shows how flexible the protein is locally, and the radius of gyration distribution (bottom) shows how dense the protein is on average. These methods make it easier to figure out how mutations change the structure and dynamics of Spike and what role they might play in virus entry and immune system evasion.

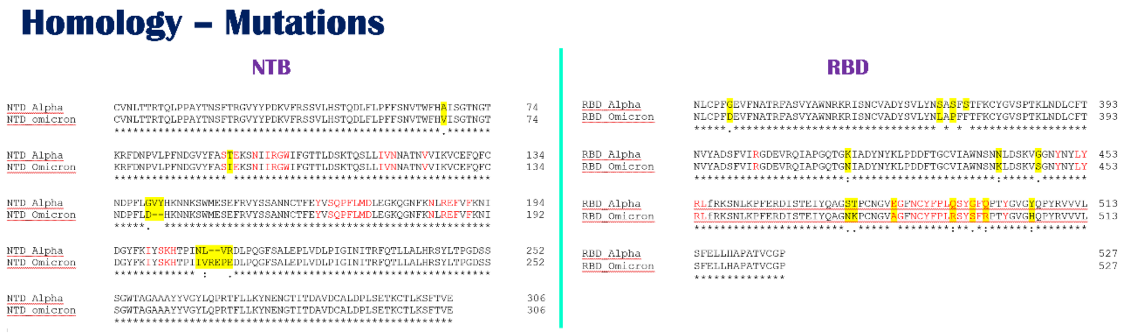

Figure S78: Analysis of Homology and Mutations in NTD and RBD Variants. The sequences line up the *N*-terminal domain (NTD) and receptor-binding domain (RBD) in the Alpha and Omicron versions, with yellow highlighting any residues that have been changed or removed. This mutation map shows specific changes that might affect structure, receptor binding, and immune evasion. It can be used as a guide to study molecular dynamics and the results of ligand–protein affinity.

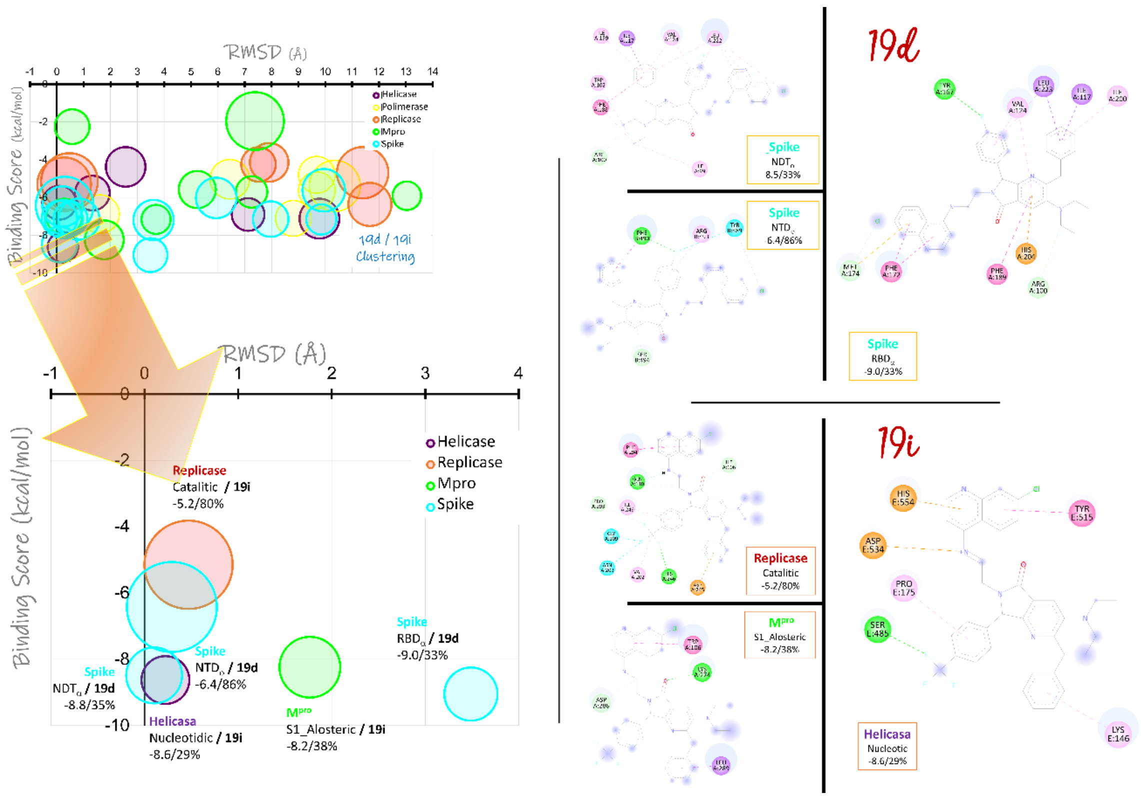

Figure S79: Bubble Plot of Binding Energy and Two-Dimensional Protein–Ligand Interaction Maps for Compounds **19d** and **19i**. In the left panel, each bubble shows a protein–ligand complex that is sorted by its RMSD (X-axis) and Binding Score (Y-axis). The size of the bubble shows how dense the population is, which is called "clustering." Systems comprising M_{pro}, replicase, helicase, and Spike (NTD and RBD) are incorporated, emphasizing the most stable and prevalent complexes. There are 2D interaction diagrams for the priority complexes on the right panel. These show hydrogen bonds, hydrophobic interactions, π–π interactions, and other noncovalent forces. This thorough method makes it easier to find a group of strong and stable interactions, which supports the idea that **19d** and **19i** could be used to stop different SARS-CoV-2 proteins.

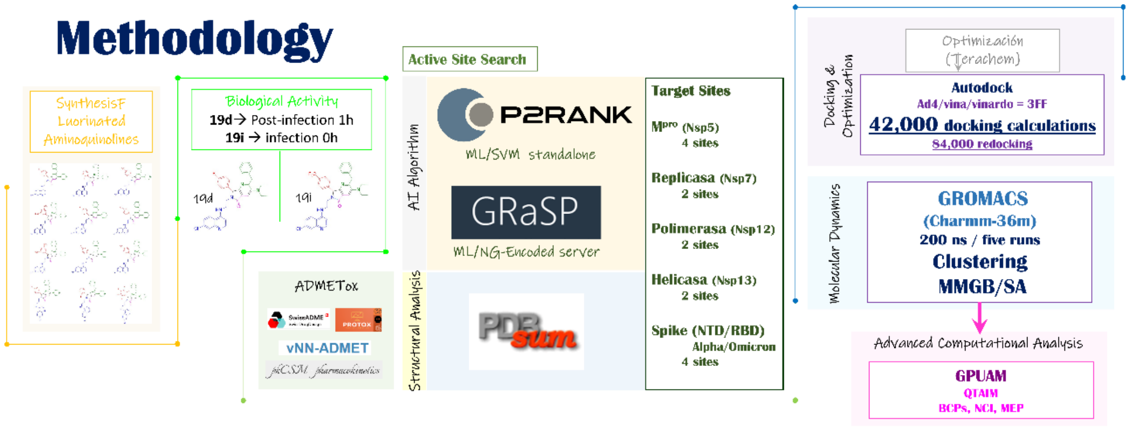

Figure S80: Comprehensive Methodology. A diagram showing how the protocol was put into action, starting with the creation and first biological testing of compounds (**19d** and **19i**), moving on to finding cavities using machine learning methods, and ending with docking procedures (including redocking and clustering) followed by molecular dynamics simulations. Throughout this approach, ADMETox studies and computational optimization techniques are employed to enhance the characterization of the compounds.

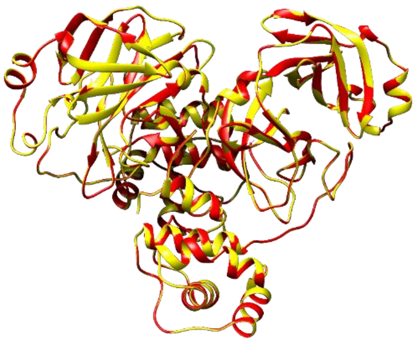

Figure S81: Superposition of the 8D35 (yellow) and 9DW6 (red) crystal structures, resolved at 1.90 Å. Both share experimental data and show an RMSD of 0.045 Å for 302 Cα, with differences less than the experimental error. The catalytic triad and ligands remain invariant, confirming their structural equivalence.

According to the PDB validation report, 8D35 and 9DW6 structures share the same resolution of 1.90 Å and they come from the same X-ray diffraction data. The additional refinement reduces the R-work/R-free from 0.182/0.224 to 0.180/0.218 without altering the model's topology.

The superposition of chains A (302 Cα atoms) yields an RMSD of 0.045 Å, which is less than the estimated coordinate error for this resolution (~0.20 Å). Per-residue analysis presents the most significant deviation (≤ 0.88 Å) in the region between amino acids 153–155, a loop located far from the active site, more than 18 Å from the catalytic center. The active site, catalytic triad, and internal ligands maintain indistinguishable positions (< 0.05 Å). Clearly, the differences fall within experimental noise.

We must recognize that the docking pocket preparation relies on the conserved geometry, and the intrinsic fluctuations in molecular dynamics exceed the initial variations. Therefore, repeating the full docking and MD pipeline would not alter the results or conclusions. Thus, we consider that the use of 8D35 does not compromise the validity of the results, and the study remains fully valid according to the current PDB version. In this regard, structural and statistical evidence shows that 9DW6 is functionally indistinguishable from 8D35 in geometric terms. The observed differences lie below experimental uncertainty and the scale of dynamic fluctuations.

Table S1: ADMETox Properties of compounds Chloroquine**, 19d** and **19i**.

Server	*Property/Parameter*	*19d*	*19i*	*Chloroquine*
SwissADME	LogP	7.8	8.2	4.2
	TPSA (Å²)	61.4	61.4	28.2
	Solubility	Moderately soluble	Moderately soluble	Moderately soluble
	GI Absorption	High	Low	High
	BBB Permeation	No	Yes	Yes
	P-glycoprotein Substrate	Yes	No	No
	Lipinski Rule Violations	0	1	0
vNN-ADMET	Hepatotoxicity	Inactive	Inactive	Inactive
	hERG Inhibition	No	No	No
	Mutagenicity	Inactive	Inactive	Yes
	CYP450 Inhibition (global)	No evidence of inhibition	No evidence of inhibition	No evidence of inhibition
	Permeability (Caco-2)	Moderate	High	---
Pred-Skin 3.0	DPRA – Prediction	---	Non-Sensitizer (–, 88.0% "Outside")	---
	KeratinoSens – Prediction	---	Non-Sensitizer (–, 49.9% "Outside")	---
	h-CLAT – Prediction	---	Non-Sensitizer (–, 59.5% "Inside")	---
	LLNA – Prediction	---	Non-Sensitizer (–, 99.9% "Outside")	---
	HRIPT/HMT – Prediction	---	Sensitizer (+, 70.8% "Outside")	---
	Bayesian Outcome	---	Sensitizer (+) – High confidence	---
ProTox-3.0	Predicted LD_{50}	500 mg/kg	500 mg/kg	750 mg/Kg
	Predicted Toxicity Class	4	4	4
	Molweight	594.12	644.13	319.87
	Octanol/Water Partition (logP)	7.53	8.41	4.88
	# H-bond Acceptors	6	6	3
	# H-bond Donors	1	1	1
	# Atoms	43	46	22
	# Bonds	48	51	23
	# Rotatable Bonds	10	11	8
	Molecular Refractivity	175.76	180.8	97.41
	Topological Polar Surface Area (TPSA)	61.36	61.36	28.16
	Hepatotoxicity	Inactive (0.69)	Inactive (0.69)	Inactive (0.90)
	Neurotoxicity	Active (0.97)	Active (0.95)	Active (0.76)
	Nephrotoxicity	Inactive (0.70)	Inactive (0.71)	Inactive (0.81)
	Respiratory	Active (0.91)	Active (0.90)	Active (0.91)
	Cardiotoxicity	Inactive (0.91)	Inactive (0.91)	Inactive (0.96)
	Carcinogenicity	Inactive (0.66)	Inactive (0.63)	Inactive (0.66)
	Immunotoxicity	Active (0.99)	Active (0.99)	Active (0.99)
	Mutagenicity	Active (0.54)	Inactive (0.50)	Active (0.94)
	Cytotoxicity	Inactive (0.63)	Inactive (0.64)	Inactive (0.93)
	BBB-barrier	Active (0.88)	Active (0.88)	Inactive (0.90)
	Ecotoxicity	Active (0.71)	Active (0.76)	Active (0.77)
	Clinical Toxicity	Active (0.75)	Active (0.76)	Active (0.56)
	Nutritional Toxicity	Inactive (0.71)	Inactive (0.64)	Inactive (0.62)
pkCSM	Molecular Weight	644.141	644.141	319.88
	LogP	8.3963	8.3963	4.8106
	# Rotatable Bonds	10	10	8
	# Acceptors	5	5	3
	# Donors	1	1	1
	Surface Area	270.398	270.398	138.230
	Water Solubility (log mol/L)	–4.337	–4.337	-4.704
	Caco2 Permeability (log Papp in 10^{-6} cm/s)	1.357	1.357	1.321
	Intestinal Absorption (% Absorbed)	88.729	88.729	90.735
	Skin Permeability (log Kp)	–2.735	–2.735	-2.586
	P-glycoprotein Substrate	Yes	Yes	Yes
	P-glycoprotein I Inhibitor	Yes	Yes	No
	P-glycoprotein II Inhibitor	Yes	Yes	No
	VDss (log L/kg)	–0.034	–0.034	1.017
	Fraction Unbound (Fu)	0.174	0.174	0.289
	BBB Permeability (log BB)	0.301	0.301	0.559
	CNS Permeability (log PS)	–1.466	–1.466	-2.471
	CYP2D6 Substrate	Yes	Yes	No
	CYP3A4 Substrate	Yes	Yes	No
	CYP1A2 Inhibitor	No	No	Yes
	CYP2C19 Inhibitor	Yes	Yes	No
	CYP2C9 Inhibitor	Yes	Yes	No
	CYP2D6 Inhibitor	No	No	No
	CYP3A4 Inhibitor	Yes	Yes	No
	Total Clearance (log ml/min/kg)	0.328	0.328	1.137
	Renal OCT2 Substrate	No	No	No
	AMES Toxicity	Yes	Yes	No
	Max. Tolerated Dose (log mg/kg/day)	0.642	0.642	0.942
	hERG I Inhibitor	No	No	No
	hERG II Inhibitor	Yes	Yes	Yes
	Oral Rat Acute Toxicity (LD_{50}, mol/kg)	3.031	3.031	3.028
	Oral Rat Chronic Toxicity (LOAEL, log mg/kg_bw/day)	0.503	0.503	0.602
	Hepatotoxicity	Yes	Yes	Yes
	Skin Sensitisation	No	No	No
	T. Pyriformis Toxicity (log ug/L)	0.285	0.285	2.441
	Minnow Toxicity (log mM)	–3.003	–3.003	0.494

Table S2: Active Site Match Using PrankWeb, GRaSP, and PDBsum.

Protein		PDB	Aminoacid of active site
M_{pro}		7P35	HIS41, MET49, PHE140, LEU141, ASN142, GLY143, SER144, CYS145, HIS163, HIS164, MET165, GLU166, PRO168, GLN189.
		8D35	LEU27, HIS41, GLY143, SER144, HIS163, HIS164, MET165, HIS172, ASP187.
		8HHU	HIS41, MET49, PHE140, LEU141, ASN142, GLY143, SER144, CYS145, HIS163, HIS164, GLU166, ASP187, GLN189.
Spike	*N*-ter-domain	7B62	ILE101, TRP104, ASN121, VAL126, MET177, ARG190, PHE192, HIS207, LEU226.
	RBD	6LZG	LYS417, TYR453, GLY496, GLY502, TYR505.
		6M0J	LYS417, TYR453, TYR505.
		6VW1	TYR453, GLY496, TYR505.
		7DF4	LEU455, GLY502, TYR505.
		7EDJ	LYS414, TYR450, PHE483, ASN484, TYR486, GLN490, TYR502.
		7KJ2	LYS417, TYR505.
		7T9K	TYR453, SER496, TYR501, HIS505.
		7U0N	TYR453, SER496, TYR501, GLY502, HIS505.
		8IUO	TYR453, HIS505.
Helicase		6XEZ_Lig	PRO283, GLY285, THR286, LYS288, ARG443, GLU540, ARG567.
		6XEZ_P-P	TYR253.
		6ZSL	No match.
		7CXN	PRO408, ASP534.
Polymerase		7B3B	LYS500, SER501, ARG569, LYS577, SER682, GLY683, ASP684, ALA685, TYR689, ASP760, SER814.
		7B3C	ASN496, LYS500, SER501, ARG569, LYS577, SER682, GLY683, ASP684, ALA685, THR687, TYR689, LEU758, SER759, ASP760, SER814.
		7BV2_Lig	ARG555, ASP623, SER682, THR687, ASN691, ASP760.
		7BV2_RNA	ASN496, LYS500, SER501, VAL557, LYS577, GLY590, SER682, GLY683, ASP684, ALA685, TYR689.
		7C2K	LYS500, SER501, LYS577, GLY590, SER682, ASP684, ALA685, SER814, ASP865.
		7CYQ_Lig	LYS50, LYS73, ARG116, ASN209, TYR217, ASP218.
		7CYQ_RNA	LYS500, SER501, ARG569, LYS577, GLY683, ASP684, ALA685, TYR689, CYS813.
		7ED5_Lig	VAL31, ARG33, PHE35, LYS50, CYS53, ARG55, VAL71, LYS73, ARG116, LEU119, LYS121, THR123, ASP208, TYR217, ASP218.
		7ED5_RNA	LYS500, SER501, ASN543, LYS577, SER682, GLY683, ASP684, ALA685, TYR689, CYS813.

Table S3: Cavity Size.

Protein			Chimera x	Chimera y	Chimera z	adjustment x	adjustment y	adjustment z	center x	center y	center z
**M_{pro}**	Allosteric Site 1		20.65	20.15	24.35	55.07	53.73	64.93	21.7	4.35	-26.75
	Allosteric Site 2		21.9	25	24.25	58.4	66.67	64.67	-15.4	-11.4	-3
**Helicase**	RNA site		16.15	18.35	24	43.07	48.93	64	136.35	207.05	135.25
	Nucleotide site		17.25	15.95	27.65	46	42.53	73.73	156.75	190.35	130.65
**Polymerase**	RNA site		25.35	24.15	21.7	67.6	64.4	57.87	187.75	200.8	255.8
	Nucleotide site		24.8	26	25.6	66.13	69.33	68.27	173.75	180.3	211.1
**Spike**	***alpha***	NTD	16.25	17.5	19.3	43.33	46.67	51.47	22.75	14.65	-17.15
		RBD	34.5	44.6	27.9	92	118.93	74.4	-33.6	31.35	4.55
	omicron	NTD	16.25	17.5	19.3	43.33	46.67	51.47	22.75	14.65	-17.15
		RBD	34.5	44.6	27.9	92	118.93	74.4	-33.6	31.35	4.55

Table S4: Better link energies with good population.

Protein-Ligand	E Average (Kcal/mol)	± ΔE (Kcal/mol)	RMSD Average	± ΔRMSD	Population	%
Spike NTDα **19d**	-9.052	0.115	3.488	0.018	451	33.407
Helicase Nucleotide Site **19i**	-8.628	0.050	0.223	0.124	366	28.706
Spike NTDo **19d**	-8.488	0.031	0.103	0.028	495	33.110
M_{pro} Allosteric Site 1 **19i**	-8.241	0.941	1.767	0.060	582	38.289
Spike NTDα **19i**	-7.193	0.183	9.855	0.052	361	24.180
Spike NTDα **19i**	-7.158	0.280	3.604	0.075	626	41.929
M_{pro} Allosteric Site S1 **19d**	-7.157	0.076	3.696	0.099	336	22.296
Spike RBDα **19i**	-7.152	0.630	0.257	0.161	562	37.145
Spike RBDo **19i**	-7.146	0.095	0.818	0.217	652	31.092
Spike NTDo **19i**	-7.144	0.021	7.969	0.005	492	33.020
Helicase Nucleotide Site **19d**	-7.119	0.271	9.782	0.081	612	45.133
Spike NTDo **19i**	-7.118	0.032	0.100	0.026	494	33.154
Spike NTDα **19d**	-7.109	0.045	0.514	0.039	446	33.037
Polymerase Nucleotide Site **19i**	-7.105	0.191	8.810	0.094	480	31.873
M_{pro} Allosteric Site 2 **19i**	-7.017	0.072	0.277	0.107	501	33.356
Helicase Nucleotide Site **19i**	-6.899	0.041	7.126	0.040	421	33.020
Polymerase RNA Site **19d**	-6.880	0.337	1.613	0.225	550	61.590
Spike RBDα **19d**	-6.479	0.282	0.215	0.084	629	41.850
Spike RBDo **19d**	-6.424	0.203	0.292	0.096	1251	85.568
Replicase Allosteric Site **19d**	-6.367	0.378	11.664	0.090	712	47.467
Helicase RNA Site **19d**	-6.278	0.282	0.091	0.127	448	38.754
Spike RBDo **19i**	-6.024	0.030	5.937	0.024	633	30.186
M_{pro} Allosteric Site 2 **19d**	-5.908	0.156	13.041	0.014	325	21.410
Helicase RNA Site **19d**	-5.763	0.287	1.323	0.148	471	40.744
M_{pro} Allosteric Site 2 **19d**	-5.707	0.645	7.257	0.130	392	26.133
Polymerase RNA Site **19i**	-5.620	0.247	9.685	0.085	505	33.577
Spike RBDo **19i**	-5.618	0.157	9.942	0.052	695	33.143
M_{pro} Allosteric Site 2 **19i**	-5.579	0.531	5.232	0.170	533	36.911
Polymerase RNA Site **19i**	-5.387	1.028	10.355	0.180	959	63.763
Replicase Catalytic Site **19d**	-5.303	0.264	0.275	0.161	1085	66.605
Replicase Catalytic Site **19i**	-5.151	0.271	0.470	0.096	1205	80.33
Polymerase Nucleotide Site **19d**	-5.032	0.574	6.445	0.080	578	45.512
Polymerase Nucleotide Site **19i**	-4.798	0.159	9.681	0.018	503	33.400
Replicase Allosteric Site **19i**	-4.689	0.839	11.391	0.205	1001	67.773
Helicase RNA Site **19i**	-4.379	0.927	2.563	0.370	607	39.364
Replicase Allosteric Site **19d**	-4.247	0.304	7.512	0.128	465	31.000
Replicase Catalytic Site **19i**	-4.138	0.118	7.910	0.027	576	31.806
M_{pro} Allosteric Site 1 **19d**	-2.249	0.194	0.571	0.256	471	33.812
M_{pro} Allosteric Site 1 **19i**	-1.953	0.371	7.385	0.206	1000	66.445

* The red mark presents the best scores in molecular docking.

Table S5: Comparative binding affinities of chloroquine, **19d**, and **19i** against selected SARS-CoV-2 targets.

Protein-Ligand	E Average (Kcal/mol)	± ΔE (Kcal/mol)	RMSD Average	± ΔRMSD	Population	%
Spike NTDα **19d**	-9.052	0.115	3.488	0.018	451	33.407
Spike NTDα **Chloroquine***	-5.518	0.64	3.602	0.204	67	22.333
Spike NTDo **19d**	-8.488	0.031	0.103	0.028	495	33.110
Spike NTDo **Chloroquine***	5.682	0.236	3.729	0.191	97	32.333
M_{pro} Allosteric Site 1 **19i**	-8.241	0.941	1.767	0.060	582	38.289
M_{pro} Allosteric Site 1 **Chloroquine***	3.882	0.694	4.893	0.130	117	39.000

* Binding affinities (kcal/mol) of chloroquine compared to compounds **19d** and **19i** against selected SARS-CoV-2 targets (M_{pro}, Spike NTD*α*, and Spike NTDo). Each value represents the best binding energy obtained from 100 molecular docking runs performed with three different scoring functions: (AD4), Vina, and Vinardo.

Table S6: Summary of Molecular Dynamics Properties.

Complex	RMSD Protein (Å)	RMSD Ligand (Å)	RG (Å)	RMSF (Peak) (Å)	Key Observations
M_{pro} – **19i**	1.5 – 3.5	2.0 – 4.5	~25.5	5 – 6	Stable ligand; moderate flexibility at the active site
nsp13 (Helicase) – **19i**	4.5 – 8.5	2.5 – 4.5	28.0 - 30.0	10 – 14	Domain reorganization; ligand remains bound
nsp7 – **19i**	1.5 – 2.5	2.0 – 4.5	~22.5	3 – 4	High overall stability; conserved coupling
Spike NTD (Omicron) – **19d**	1.5 – 3.0	2.0 – 3.5	~20.2	5 – 6	Stable binding in mobile loops; general structure conserved
Spike RBD (Alfa) – **19d**	2.0 – 3.5	2.5 – 4.5	~18.5	5 – 6 (470–490)	Persistent union; functional flexibility at the *binding ridge*
Spike RBD (Omicron) – **19d**	2.0 – 5.0	2.5 – 4.5	18.0 – 18.7	6 – 9 (470–490)	Adaptive ligand; increased flexibility in mutated regions

Table S7: NTDα MM/GBSA Decomposition.

Frames	VDWAALS	EEL	EGB	ESURF	GGAS	GSOLV	Total
**Average**	-55.37	-8.24	43.14	-7.21	-63.61	35.93	-27.68
**SD**	5.3	8.77	8.85	0.52	11.02	8.7	4.29
**SEM**	0.03	0.06	0.06	0	0.07	0.06	0.03

Table S8: NTDα Per-Amino Acid GB Δ_TDC.

Frames	Average	SD	SEM
**R:A:GLU:94**	1.19	0.45	0
**R:A:ARG:100**	-0.56	0.36	0
**R:A:GLY:101**	-0.56	0.22	0
**R:A:ILE:117**	-0.65	0.26	0
**R:A:ASN:119**	-0.94	0.54	0
**R:A:VAL:124**	-2.02	0.44	0
**R:A:PHE:171**	-2.36	0.52	0
**R:A:ARG:186**	-1.74	0.85	0.01
**R:A:PHE:188**	-0.75	0.43	0
**R:A:SER:201**	-1.73	1.47	0.01
**R:A:LYS:202**	-1.67	1.12	0.01
**R:A:HSD:203**	-0.91	0.68	0
**R:A:GLU:220**	0.75	0.84	0.01
**R:A:LEU:222**	-1.63	0.7	0
**L:B:LE9:304**	-16.02	2.68	0.02

Table S9: NTDo MM/GBSA Decomposition.

Frames	VDWAALS	EEL	EGB	ESURF	GGAS	GSOLV	Total
**Average**	-58.14	-8.72	43.04	-7.25	-66.86	35.79	-31.07
**SD**	4.66	7.11	7.03	0.46	8.79	6.91	5.13
**SEM**	0.03	0.05	0.04	0	0.06	0.04	0.03

Table S10: NTDo Per-Amino Acid GB Δ_TDC.

Frames	Average	SD	SEM
**R:A:GLU:94**	0.91	0.35	0
**R:A:GLY:101**	-0.62	0.25	0
**R:A:ILE:117**	-0.75	0.34	0
**R:A:ASN:119**	-0.91	0.42	0
**R:A:VAL:124**	-2.12	0.44	0
**R:A:PHE:172**	-2.32	0.64	0
**R:A:MET:174**	-0.58	0.54	0
**R:A:ARG:187**	-1.53	0.97	0.01
**R:A:PHE:189**	-0.6	0.35	0
**R:A:SER:202**	-0.53	0.4	0
**R:A:LEU:223**	-2.21	0.67	0
**L:B:LE9:305**	-14.25	2.53	0.02

Table S11: nsp5 MM/GBSA Decomposition.

Frames	VDWAALS	EEL	EGB	ESURF	GGAS	GSOLV	Total
**Average**	-35.71	2.5	17.73	-4.77	-33.21	12.97	-20.24
**SD**	7.97	7.75	7.7	1.09	11.32	7.25	6.03
**SEM**	0.05	0.05	0.05	0.01	0.07	0.05	0.04

Table S12: nsp5 Per-Amino Acid GB Δ_TDC.

Frames	Average	SD	SEM
**R:A:VAL:104**	-0.51	0.57	0
**R:A:GLN:107**	-1.06	1.17	0.01
**R:A:GLN:110**	-0.91	0.9	0.01
**R:A:ASP:153**	0.81	0.94	0.01
**R:A:ILE:249**	-1.79	1.21	0.01
**R:A:PHE:294**	-2.21	1.39	0.01
**L:C:LE3:306**	-10.89	3.06	0.02

Supplementary Movie 1: The spike protein's mechanical movements and the potential mechanisms by which **19d** blocks one of the spike's wings are both visible in the film. It tries to explain how the NTD domain moves in solutions and how it recognizes its substrate. Low conformational plasticity is concentrated in the active site region of the NTD variants alpha and omicron, as predicted by the analysis, and in fact may extend to the full conformation of one of the spike's wings. PHE172 is a key residue in this area that helps form hydrophobic subsites. These residues, together with VAL124 and LEU223, create a substrate recognition cavity that can hold various hydrophobic side chains.

https://drive.google.com/file/d/19WtSNAw1j76Nog10pCR6FwTQ4f7CvXU-/view?usp=sharing

Supplementary Movie 2: The film allows us to observe the mechanical motions, enabling us to characterize M_{pro} in solution and understand the **19i** substrate recognition. The study shows, as expected, the highest conformational plasticity concentrated in the active site region of M_{pro}, which comprises important residues engaged in the formation of the hydrophobic subsite, including PHE62. Together with ILE247, GL105 forms a space that helps the enzyme recognize and interact with hydrophobic side chains like leucine and valine.

https://drive.google.com/file/d/19X2jr4INCjL2jsnMxzSiWt1VjZI3nWNU/view?usp=sharing
